# Determinants of cognitive performance and decline in 20 diverse ethno-regional groups: A COSMIC collaboration cohort study

**DOI:** 10.1371/journal.pmed.1002853

**Published:** 2019-07-23

**Authors:** Darren M. Lipnicki, Steve R. Makkar, John D. Crawford, Anbupalam Thalamuthu, Nicole A. Kochan, Maria Fernanda Lima-Costa, Erico Castro-Costa, Cleusa Pinheiro Ferri, Carol Brayne, Blossom Stephan, Juan J. Llibre-Rodriguez, Jorge J. Llibre-Guerra, Adolfo J. Valhuerdi-Cepero, Richard B. Lipton, Mindy J. Katz, Carol A. Derby, Karen Ritchie, Marie-Laure Ancelin, Isabelle Carrière, Nikolaos Scarmeas, Mary Yannakoulia, Georgios M. Hadjigeorgiou, Linda Lam, Wai-chi Chan, Ada Fung, Antonio Guaita, Roberta Vaccaro, Annalisa Davin, Ki Woong Kim, Ji Won Han, Seung Wan Suh, Steffi G. Riedel-Heller, Susanne Roehr, Alexander Pabst, Martin van Boxtel, Sebastian Köhler, Kay Deckers, Mary Ganguli, Erin P. Jacobsen, Tiffany F. Hughes, Kaarin J. Anstey, Nicolas Cherbuin, Mary N. Haan, Allison E. Aiello, Kristina Dang, Shuzo Kumagai, Tao Chen, Kenji Narazaki, Tze Pin Ng, Qi Gao, Ma Shwe Zin Nyunt, Marcia Scazufca, Henry Brodaty, Katya Numbers, Julian N. Trollor, Kenichi Meguro, Satoshi Yamaguchi, Hiroshi Ishii, Antonio Lobo, Raul Lopez-Anton, Javier Santabárbara, Yvonne Leung, Jessica W. Lo, Gordana Popovic, Perminder S. Sachdev, for Cohort Studies of Memory in an International Consortium (COSMIC)

**Affiliations:** 1 Centre for Healthy Brain Ageing, University of New South Wales, Sydney, New South Wales, Australia; 2 Instituto René Rachou, Fundação Oswaldo Cruz, Rio de Janeiro, Brazil; 3 Departamento de Psiquiatria, Universidade Federal de São Paulo, São Paulo, Brazil; 4 Department of Public Health and Primary Care, Cambridge University, Cambridge, United Kingdom; 5 Institute of Health and Society, Newcastle University, Newcastle upon Tyne, United Kingdom; 6 Finlay-Albarrán Faculty of Medical Sciences, Medical University of Havana, Havana, Cuba; 7 Institute of Neurology and Neurosurgery, Havana, Cuba; 8 Memory and Aging Center, University of California, San Francisco, San Francisco, California, United States of America; 9 Medical University of Matanzas, Matanzas, Cuba; 10 Saul R. Korey Department of Neurology, Albert Einstein College of Medicine, Yeshiva University, New York, New York, United States of America; 11 Department of Epidemiology and Population Health, Albert Einstein College of Medicine, Yeshiva University, New York, New York, United States of America; 12 Department of Psychiatry and Behavioral Medicine, Albert Einstein College of Medicine, Yeshiva University, New York, New York, United States of America; 13 Inserm, U1061 Neuropsychiatry: Epidemiological and Clinical Research, La Colombière Hospital, Montpellier, France; 14 Université de Montpellier, Montpellier, France; 15 Centre for Clinical Brain Sciences, University of Edinburgh, Edinburgh, United Kingdom; 16 1st Department of Neurology, Aiginition Hospital, Medical School, National and Kapodistrian University of Athens, Athens, Greece; 17 Taub Institute for Research on Alzheimer’s Disease and the Aging Brain, Gertrude H. Sergievsky Center, Department of Neurology, Columbia University, New York, New York, United States of America; 18 Department of Nutrition and Dietetics, Harokopio University, Athens, Greece; 19 University of Thessaly, Larissa, Greece; 20 Department of Neurology, Medical School, University of Cyprus, Nicosia, Cyprus; 21 Department of Psychiatry, Chinese University of Hong Kong, Hong Kong SAR, China; 22 Department of Psychiatry, University of Hong Kong, Hong Kong SAR, China; 23 Department of Applied Social Sciences, Hong Kong Polytechnic University, Hong Kong SAR, China; 24 Golgi Cenci Foundation, Milan, Italy; 25 Department of Neuropsychiatry, Seoul National University Bundang Hospital, Seongnam, Korea; 26 Department of Psychiatry, College of Medicine, Seoul National University, Seoul, Korea; 27 Department of Brain and Cognitive Science, College of Natural Sciences, Seoul National University, Seoul, Korea; 28 Institute of Social Medicine, Occupational Health and Public Health, Medical Faculty, University of Leipzig, Leipzig, Germany; 29 Department of Psychiatry and Neuropsychology, School for Mental Health and Neuroscience, Maastricht University Medical Center, Maastricht, The Netherlands; 30 Department of Psychiatry, School of Medicine, University of Pittsburgh, Pittsburgh, Pennsylvania, United States of America; 31 Department of Neurology, School of Medicine, University of Pittsburgh, Pittsburgh, Pennsylvania, United States of America; 32 Department of Epidemiology, Graduate School of Public Health, University of Pittsburgh, Pittsburgh, Pennsylvania, United States of America; 33 Department of Sociology, Anthropology, and Gerontology, Youngstown State University, Youngstown, Ohio, United States of America; 34 School of Psychology, University of New South Wales, Sydney, New South Wales, Australia; 35 Neuroscience Research Australia, Sydney, New South Wales, Australia; 36 Centre for Research on Ageing, Health and Wellbeing, Australian National University, Canberra, Australian Capital Territory, Australia; 37 Department of Epidemiology and Biostatistics, School of Medicine, University of California, San Francisco, San Francisco, California, United States of America; 38 Department of Epidemiology, Gillings School of Global Public Health, University of North Carolina at Chapel Hill, Chapel Hill, North Carolina, United States of America; 39 Carolina Population Center, University of North Carolina at Chapel Hill, Chapel Hill, North Carolina, United States of America; 40 Center for Health Science and Counseling, Kyushu University, Kasuga, Japan; 41 Faculty of Socio-Environmental Studies, Fukuoka Institute of Technology, Fukuoka, Japan; 42 Gerontology Research Programme, Department of Psychological Medicine, Yong Loo Lin School of Medicine, National University of Singapore, Singapore; 43 Department of Psychological Medicine, Yong Loo Lin School of Medicine, National University of Singapore, Singapore; 44 Instituto de Psiquiatria e LIM-23, Hospital da Clínicas, Faculdade de Medicina, Universidade de São Paulo, São Paulo, Brazil; 45 Dementia Collaborative Research Centre, University of New South Wales, Sydney, New South Wales, Australia; 46 Department of Developmental Disability Neuropsychiatry, School of Psychiatry, University of New South Wales, Sydney, New South Wales, Australia; 47 Geriatric Behavioral Neurology, Tohoku University, Sendai, Japan; 48 Department of Medicine and Psychiatry, Universidad de Zaragoza, Zaragoza, Spain; 49 Instituto de Investigación Sanitaria Aragón, Zaragoza, Spain; 50 Centro de Investigación Biomédica en Red de Salud Mental, Ministry of Science and Innovation, Madrid, Spain; 51 Department of Psychology and Sociology, Universidad de Zaragoza, Zaragoza, Spain; 52 Department of Microbiology, Preventive Medicine and Public Health, Universidad de Zaragoza, Zaragoza, Spain; 53 School of Mathematics and Statistics, University of New South Wales, Sydney, New South Wales, Australia; Massachusetts General Hospital, UNITED STATES

## Abstract

**Background:**

With no effective treatments for cognitive decline or dementia, improving the evidence base for modifiable risk factors is a research priority. This study investigated associations between risk factors and late-life cognitive decline on a global scale, including comparisons between ethno-regional groups.

**Methods and findings:**

We harmonized longitudinal data from 20 population-based cohorts from 15 countries over 5 continents, including 48,522 individuals (58.4% women) aged 54–105 (mean = 72.7) years and without dementia at baseline. Studies had 2–15 years of follow-up. The risk factors investigated were age, sex, education, alcohol consumption, anxiety, apolipoprotein E ε4 allele (APOE*4) status, atrial fibrillation, blood pressure and pulse pressure, body mass index, cardiovascular disease, depression, diabetes, self-rated health, high cholesterol, hypertension, peripheral vascular disease, physical activity, smoking, and history of stroke. Associations with risk factors were determined for a global cognitive composite outcome (memory, language, processing speed, and executive functioning tests) and Mini-Mental State Examination score. Individual participant data meta-analyses of multivariable linear mixed model results pooled across cohorts revealed that for at least 1 cognitive outcome, age (*B* = −0.1, SE = 0.01), APOE*4 carriage (*B* = −0.31, SE = 0.11), depression (*B* = −0.11, SE = 0.06), diabetes (*B* = −0.23, SE = 0.10), current smoking (*B* = −0.20, SE = 0.08), and history of stroke (*B* = −0.22, SE = 0.09) were independently associated with poorer cognitive performance (*p* < 0.05 for all), and higher levels of education (*B* = 0.12, SE = 0.02) and vigorous physical activity (*B* = 0.17, SE = 0.06) were associated with better performance (*p* < 0.01 for both). Age (*B* = −0.07, SE = 0.01), APOE*4 carriage (*B* = −0.41, SE = 0.18), and diabetes (*B* = −0.18, SE = 0.10) were independently associated with faster cognitive decline (*p* < 0.05 for all). Different effects between Asian people and white people included stronger associations for Asian people between ever smoking and poorer cognition (group by risk factor interaction: *B* = −0.24, SE = 0.12), and between diabetes and cognitive decline (*B* = −0.66, SE = 0.27; *p* < 0.05 for both). Limitations of our study include a loss or distortion of risk factor data with harmonization, and not investigating factors at midlife.

**Conclusions:**

These results suggest that education, smoking, physical activity, diabetes, and stroke are all modifiable factors associated with cognitive decline. If these factors are determined to be causal, controlling them could minimize worldwide levels of cognitive decline. However, any global prevention strategy may need to consider ethno-regional differences.

## Abstract 2

Darren Lipnicki and the Sydney COSMIC team analysed cognitive performance and risk factor data from older people from 15 countries to assess associations between risk factors and late-life cognition

## Author summary

**Why was this study done?:**

- There is a growing global burden associated with cognitive decline and dementia.
- Evidence for modifiable risk factors that could be targeted to reduce this burden needs to be improved.
- Risk factors for cognitive decline and dementia might differ between ethno-regional groups.

**What did the researchers do and find?:**

- We analyzed cognitive performance and risk factor data from 48,522 older individuals, provided by 20 studies of aging representing 15 countries (Australia, Brazil, Cuba, France, Germany, Greece, Hong Kong, Italy, Japan, Singapore, Spain, South Korea, The Netherlands, the United Kingdom, and the United States). Cognitive performance was measured as Mini-Mental State Examination (MMSE) and global cognition scores.
- When controlling for confounding risk factors, the apolipoprotein E ε4 allele (APOE*4), depression, diabetes, current smoking, and history of stroke were associated with poorer cognitive performance, and higher levels of education and vigorous physical activity were associated with better performance. Age, APOE*4, and diabetes were associated with faster cognitive decline.
- Compared to white people, Asian people showed stronger associations between having ever smoked and cognitive performance, and between diabetes and cognitive decline.

**What do these findings mean?:**

- Modifiable risk factors associated with cognitive decline include education, smoking, physical activity, diabetes, and stroke.
- If these associations are determined to be causal, interventions targeting these factors may help reduce the global burden of cognitive decline and dementia.
- Interventions may require tailoring to particular ethno-regional groups.

## Introduction

Whether a normal aspect of the aging process or a consequence of neuropathological changes arising from many possible causes, cognitive decline is almost invariably associated with growing old [1,2]. The world’s population is aging rapidly, and with no effective therapies, the global financial and societal burdens of cognitive decline are set to rise. Modifiable risk factors for cognitive decline offer means to intervene and prevent or delay these rising global burdens. However, the evidence base for associations between modifiable risk factors and cognitive decline remains poor, and improving this is a research priority [1,3].

This study aims to improve the understanding of associations between risk factors and late-life cognitive performance and decline. The first of 3 distinctive features of this study is a comprehensive examination of risk factors, with a large range of demographic, medical, lifestyle, and physical and mental health factors investigated. Second is the global scale, with data obtained from 20 population-based cohorts from 15 countries across 5 continents. Third is the use of individual participant data (IPD), which has advantages over aggregate approaches, including the ability to standardize analyses across samples and to adjust for confounding factors [4]. Having previously identified faster rates of cognitive decline in Asian people than in white people [5], we also investigate whether the effects of risk factors differ between Asian people and white people in this analysis. This study was done to identify and further assess risk factors for poorer cognitive performance and decline in late life, and to determine whether associations between particular factors and cognition are global.

## Methods

### Contributing studies

All 20 contributing cohorts were population-based and members of the Cohort Studies of Memory in an International Consortium (COSMIC) collaboration [6], and are detailed in Table 1. Fourteen of these cohorts were included in our previous report on cognitive decline [5]. The additional 6 cohorts included in this study are CHAS, LEILA75+, MAAS, MoVIES, SALSA, and Tajiri, which expand the number of countries covered by 3 (Cuba, Germany, and The Netherlands) and increase the representation of Latin Americans and North American Hispanics. Full cohorts were generally not used, as we excluded individuals with dementia at baseline or who were missing age, sex, education, or dementia status data (typically less than 1%; S1 and S2 Tables). The cohorts had various assessment schedules (2–16 waves) and follow-up durations (2–15 years; S3 Table). This project was approved by the University of New South Wales Human Research Ethics Committee (HC 12446 and HC 17292). The contributing cohorts had prior ethics approval (S4 Table). This study is reported as per the Strengthening the Reporting of Observational Studies in Epidemiology (STROBE) guideline (S1 Checklist), and the prospective analysis plan is provided as S1 Text.

**Table 1:** Contributing studies (in alphabetical order).

Study	Abbreviation	Location	Main race/ethnicity	Years run^a^	Reference
Bambui Cohort Study of Ageing	Bambui	Bambui, Brazil	Brazilian	1997–2013	Lima-Costa et al. [7]
Cognitive Function & Ageing Study	CFAS	United Kingdom^b^	White	1989–	Brayne et al. [8]
Cuban Health and Alzheimer Study	CHAS	Havana and Matanzas, Cuba	White, black, mixed^c^	2003–	Llibre-Rodriguez et al. [9]
Einstein Aging Study	EAS	New York, US	White, black^d^	1993–	Katz et al. [10]
Etude Santé Psychologique Prévalence Risques et Traitement	ESPRIT	Montpellier, France	White	1999–	Ritchie et al. [11]
Hellenic Longitudinal Investigation of Aging and Diet	HELIAD	Larissa and Marousi, Greece	White	2010–	Dardiotis et al. [12]
Hong Kong Memory and Ageing Prospective Study	HK-MAPS	Hong Kong	Chinese	2005–	Wong et al. [13]
Invecchiamento Cerebrale in Abbiategrasso	Invece.Ab	Abbiategrasso, Italy	White	2010–2015	Guaita et al. [14]
Korean Longitudinal Study on Cognitive Aging and Dementia	KLOSCAD	South Korea (nationwide)	Korean	2009–2018	Han et al. [15]
Leipzig Longitudinal Study of the Aged	LEILA75+	Leipzig, Germany	White	1997–2014	Riedel-Heller et al. [16]
Maastricht Aging Study^e^	MAAS	South Limburg, The Netherlands	White	1993–2018	Jolles et al. [17]
Monongahela Valley Independent Elders Survey	MoVIES	Mid-Monongahela Valley, PA, US	White	1987–2002	Ganguli et al. [18]
Personality and Total Health Through Life Project	PATH	Canberra, Australia	White	2001–	Anstey et al. [19]
Sacramento Area Latino Study on Aging	SALSA	Sacramento area, CA, US	Hispanic, Mexican ancestry	1998–2008	Haan et al. [20]
São Paulo Ageing & Health Study	SPAH	São Paulo, Brazil	Brazilian	2003–2008	Scazufca et al. [21]
Sasaguri Genkimon Study	SGS	Sasaguri, Japan	Japanese	2011–	Narazaki et al. [22]
Singapore Longitudinal Ageing Studies (I)	SLASI	Singapore	Chinese	2003–	Feng et al. [23]
Sydney Memory and Ageing Study	Sydney MAS	Sydney, Australia	White	2005–	Sachdev et al. [24]
Tajiri Project	Tajiri	Tajiri, Japan	Japanese	1998–2005	Meguro et al. [25]
Zaragoza Dementia Depression Project	ZARADEMP	Zaragoza, Spain	White	1994–	Lobo et al. [26]

^a^Studies without an end date are ongoing.

^b^Five identical centers including Cambridgeshire, Gwynedd, Newcastle, Nottingham, and Oxford.

^c^White 72.4%, black 16.5%, mixed 11.0%.

^d^White 66.5%, black 27.6%.

^e^Only participants aged 55 years or more at baseline were included.

### Measures

All cohorts provided age and sex data. Educational attainment was provided as years in full-time education or converted to this from categorical data (S5 Table).

Our first cognitive outcome measure was a brief global cognitive or screening test: the Mini-Mental State Examination (MMSE) [27] in 16 cohorts (the 4 exceptions are detailed in S6 Table). The second measure was a global cognition composite score, calculated from 4 neuropsychological tests, each representing 1 of 4 cognitive domains. Cohorts varied in the neuropsychological tests administered, so for each domain we used a single common test or type: delayed word list recall for memory, semantic fluency for language, and Trail Making Test A and B for processing speed and executive functioning, respectively (S6 Table).

The baseline factors investigated were age, sex, education, current alcohol consumption (nil/minimal, 1 drink/week, 2+ drinks/week), anxiety, apolipoprotein E ε4 allele (APOE*4) carriage (having at least 1 ε4 allele), atrial fibrillation, blood pressure and pulse pressure, body mass index (BMI), cardiovascular disease, depression (both current and history of), diabetes, health (self-reported: very good, good, or poor), high cholesterol (either total cholesterol or triglycerides), hypertension, peripheral vascular disease, physical activity (minimal, or moderate or vigorous at least once a week), smoking (never, past, current), and history of stroke. For some of these factors, while particular levels may be considered as increasing risk, others may be considered as protective. For simplicity we refer to all of the factors investigated as risk factors. Most cohorts lacked data for some factors (S7 Table). The available data for some factors were comparable across the contributing studies as presented (APOE*4, BMI, blood pressure and pulse pressure, and smoking). Other factors required harmonization because the type and number of measures for these varied across the studies. For binary medical and mental health condition variables (anxiety, current depression, history of depression, hypertension, diabetes, high cholesterol, peripheral vascular disease, atrial fibrillation, cardiovascular disease, and stroke), we used all available information from a study relevant to diagnosing or classifying the condition. For example, in a study with limited information, hypertension may be classified only from a medical history record, while for another study, it may be indicated by any of self-reported history, use of relevant medication, or measured blood pressure exceeding values indicated by international guidelines. Some factors required further work to harmonize, including the transformation or collapsing of original responses into a standard format (alcohol consumption, health, and physical activity). This involved a more subjective approach: For example, the division of general health responses into 3 categories for some studies was informed by the distribution of individuals across categories in the majority of studies (i.e., smaller proportions endorsing each of the best and worst health levels than the middle level). Physical activity was the most difficult factor to harmonize, given the large variety of measures across the studies, from simple questions on general activity to more involved questionnaires that specified activities and the frequency and duration these were participated in. Our harmonization protocols for all of the factors, including the specific conditions included in factors like cardiovascular disease, are fully detailed in S8–S20 Tables.

### Statistical analysis

#### Standardization of outcome scores

First, within each study, raw MMSE and domain scores, pooled across all waves, were transformed to have a Gaussian (or normal) distribution, calculated so that the transformed value has the same percentile value as the original value in the original distribution (in SPSS such scores are described simply as normal scores, but are produced under the Rank Cases procedure). Transformed score outliers were then winsorized to values plus or minus 3 standard deviations (SDs) from the mean scores. These were then standardized by converting to *Z*-scores within each study, using estimated means and SDs of baseline scores within each study at common values of age, sex, and education. The common values were the average values at baseline from data pooled across all studies (common values: age = 72.7 years, education = 9.0 years, and sex = 0.42, indicating 42% males). SDs used for the calculation of the *Z*-scores were the estimated SDs of the residuals (i.e., the standard errors [SEs] of the estimates) obtained from the regression models for each study after adjustment for age, sex, and education. Our method of standardizing scores from multiple studies is essentially the same as that described by Griffith et al. [28] for obtaining standardized demographically based category-centered scores. However, instead of obtaining *Z-*scores using means and SDs from subsamples within each study with the same restricted ranges of demographic characteristics, we used regression models to obtain estimated means and SDs for specific common values of demographic variables. The global cognition composite score was computed by averaging the standardized domain scores, and then standardizing the average scores using the same process described above. A global cognition composite score was computed for individuals with data for at least 3 cognitive domains.

#### Associations of age at baseline, sex, and education with cognitive performance/decline

Our analyses used linear mixed models, which are widely used to analyze longitudinal data and are recommended to address missing data as well as to reduce non-random attrition bias. Linear mixed models were used to examine the associations of age at baseline, sex, and education with cognitive performance evaluated at a common value of “time in study” equal to the mean value for the combined sample, as well as cognitive decline (per decade) on each outcome, separately for each cohort. These models included fixed effect terms for age at baseline (centered at the mean of 72.7 years), age at baseline squared, sex (centered at the proportion of 42% males), education (centered at the mean of 9.0 years), time in study (centered at the mean across all studies of 3.1 years), time in study squared, interactions between age at baseline and each of education and sex, and interactions between time and each of age at baseline, sex, and education (the full model equation is displayed in S1 Text). This selection of model terms was based on preliminary variable selection analyses, such that terms explaining at least 0.1 percent of variance on at least 1 outcome measure were retained (see S1 Text for details). The variables were centered to reduce collinearity between the variables and quadratic or interaction terms. Note that since time in study was centered at 3.1 years, the effects of predictors on cognition were for performance at this time in study. The intercept and time terms were treated as random effects, with an unstructured covariance type. Using a method described by Singer and Willet [29], we investigated the possible influence of baseline performance on cognitive decline by examining the covariance between the random effects of intercept and slope. For these analyses, time was coded to have the value 0 at baseline, so that the covariance term quantifies the relationship between individuals’ performance at baseline and their rate of change. In the presence of an overall decline in performance over time, a positive (or negative) covariance term implies that a lower performance at baseline is associated with a faster (or slower) rate of decline.

#### Associations between putative risk factors and cognitive performance/decline: Partially adjusted multivariable models

Associations between each potential risk factor and performance/decline for each cognitive outcome were examined using linear mixed models. The models included the following fixed effect terms: age at baseline, age at baseline squared, education, sex, age at baseline × education, the factor being analyzed, time in study, time in study squared, and 2-way interactions between the factor and each of time in study, time in study squared, age at baseline, sex, and education (the full model equation is displayed in S1 Text). Preliminary analyses indicated quadratic associations between cognitive performance and both education and BMI, consistent with previous reports [30,31]. For this reason, in analyses where BMI or education were examined, quadratic terms for these were included, as well as their 2-way interactions with age, age squared, education, sex, time in study, and time in study squared. Separate linear mixed models were produced for each factor and outcome combination. The intercept and time terms were included as random terms, with an unstructured covariance type. While these models investigating risk factors 1 by 1 all included age, sex, and education, we refer to them as partially adjusted models to distinguish them from later models featuring multiple risk factors, which are referred to as fully adjusted models.

We used random effects IPD meta-analysis to pool the cohort-wise linear mixed model results to obtain pooled estimates of effect sizes for each of the model terms. Of particular interest were the associations between each factor (including age at baseline, sex, and education) and cognitive performance, the association between each factor and cognitive decline (i.e., the fixed effect of factor × time in study), and the association between each factor and change in the rate of cognitive decline (i.e., the fixed effect of factor × time in study squared). A negative pooled value for the factor term would indicate that the factor was associated with a lower level of cognitive performance at the average time in study of 3.1 years. A negative pooled value for the time in study term would indicate that cognition declined over time in the absence of the factor (or at its mean value, for continuous factors). Therefore, a negative pooled value for the factor × time interaction term would indicate that having the factor was associated with a faster rate of decline than not having the factor. A significant negative pooled value for the factor × time in study squared term would indicate that the presence of the factor is associated with a faster rate of decline with increasing time in study.

#### Associations between putative risk factors and cognitive performance/decline: Fully adjusted multivariable models

For each cognitive outcome, we fit multivariable models that featured multiple factors, and are thus termed fully adjusted, to determine independent associations with cognitive performance/decline. Not all the factors investigated above were included in these models as not all were available for every cohort, with the choice of data used involving a compromise between the number of factors included and the number of cohorts with data for all factors. The first model (fully adjusted model 1) used data from 11 cohorts (Bambui, EAS, ESPRIT, HELIAD, HK-MAPS, Invece.Ab, KLOSCAD, PATH, SALSA, SLASI, and Sydney MAS; *N =* 13,917) and included alcohol consumption, APOE*4 status, cardiovascular disease, diabetes, high cholesterol, hypertension, smoking, and history of stroke, as well as age, sex, and education. Also included were the interactions between each factor and age, age squared, education, sex, time in study, and time in study squared. Model terms from within each cohort were pooled using random effects IPD meta-analysis using the same process described above. This model was repeated with APOE*4 status replaced with either BMI (using the same cohorts except for SGS replacing HK-MAPS; *N =* 17,270) or current depression (using the same cohorts except for SGS replacing SLASI; *N =* 18,011). Separate multivariable models were fit because combining APOE*4, BMI, and current depression in 1 model (either together or in pairs) meant that the analyses would only include a maximum of 2 cohorts from Asian countries. We reasoned that a minimum of 3 cohorts from Asian countries was required to perform meaningful comparisons with cohorts of white people (see below). A second fully adjusted model (fully adjusted model 2) used data from only 9 cohorts (Bambui, EAS, ESPRIT, HELIAD, Invece.Ab, KLOSCAD, PATH, SALSA, and Sydney MAS; *N =* 11,897) but simultaneously investigated a larger set of factors: alcohol consumption, APOE*4 status, BMI, cardiovascular disease, depression, diabetes, high cholesterol, hypertension, physical activity, smoking, and history of stroke. Categorical factors with 3 levels were also examined as binary factors by collapsing the higher levels and comparing against the lowest (e.g., past and current smoking collapsed to ever smoking and compared against never smoked). This allowed for the inclusion of cohorts with only binary level data on these factors. We report significant results for both the binary and multi-level versions of each factor, to better understand both the overall and dose–response relationship of a factor’s effects. We also investigated whether baseline cognitive performance was related to rate of change in each of our fully adjusted models, using the same approach as for the partially adjusted models described above. Heterogeneity of effect sizes among samples was assessed using the *I*^2^ statistic, reflecting the proportion of variation due to variability between studies, rather than sampling error or chance. We report *I*^2^ values obtained from fixed effects models, which give appropriate indications of variation across studies. *I*^2^ values of <40%, 40%–60%, and >60% were taken to indicate low, moderate, and substantial heterogeneity, respectively, as per Cochrane Collaboration guidelines [32].

#### Ethno-regional comparisons

We examined whether relationships between factors and cognitive performance/decline differed between groups of white people and Asian people; included in the white group were all individuals self-reporting or classified as a white person from 11 cohorts of predominantly white people (CFAS, EAS, ESPRIT, HELIAD, Invece.Ab, LEILA75+, MAAS, MoVIES, PATH, Sydney MAS, and ZARADEMP), and included in the Asian group were all individuals from 5 cohorts in countries with majority Asian populations (HK-MAPS, KLOSCAD, SGS, Tajiri, and SLASI, with the last cohort comprising 95.6% Chinese, 1.8% Malay, 2.1% Indian, and 0.6% other). Individuals from the Latin American and North American Hispanic cohorts (Bambui, CHAS, SPAH, and SALSA) were not included in these groups. We performed the partially adjusted and fully adjusted model 1 (including repeats with BMI or current depression replacing APOE*4 status) linear mixed models described above using these groups, with the cohort-wise mixed model results pooled and differences between white people and Asian people tested using meta-regression. A binary, cohort-level variable (white person = 0, Asian person = 1) was included as a moderator term in the meta-regression. A significant moderator term would indicate a significant difference in the effect between white people and Asian people. Results for model terms were also pooled within the cohorts of white people or Asian people separately using random effects meta-analysis. The size, direction, and significance of model terms were examined within each group to help interpret significant moderator effects.

The Sydney COSMIC team created the pooled dataset and ran the linear mixed models using IBM SPSS Statistics 24. The meta-analyses were performed using the Metan and Metareg packages in Stata 13. Two-sided *p-*values were used, with an α = 0.05 threshold for statistical significance.

## Results

### Sample description

The overall sample comprised 48,522 eligible individuals contributed by the 20 cohorts. Their baseline age ranged from 54 to 105 years (mean = 72.7 ± SD 7.5), and 58.4% were women. The mean number of years of education was 9.0 (SD = 4.5) years. S21 Table shows cohort-specific demographic characteristics, and S22 and S23 Tables show the prevalence and mean values for the factors investigated.

### Associations between factors and cognitive performance

Across all 20 cohorts, significant associations were found using both partially and fully adjusted multivariable models. We focus here on the fully adjusted model results, though later summarize the partially adjusted model results (full details in S2 Text and S24–S26 Tables). As shown in Table 2, fully adjusted models identified increased age, APOE*4 carriage, depression, diabetes, history of stroke, and current smoking as independently associated with lower scores for 1 or both of the MMSE and global cognition composite. Heterogeneity of these effects was low (*I*^2^ values ranging between 0% and 26.4%), except for age (moderate heterogeneity in model 2: 56.2%) and APOE*4 carriage (moderate heterogeneity in both models 1 and 2: 48.2% and 40.2%, respectively). Conversely, cardiovascular disease, higher education, and vigorous physical activity were associated with better cognitive performance. Heterogeneity for associations with cardiovascular disease and vigorous physical activity was low across models (*I*^2^ < 40%). For education, however, heterogeneity was high in each model (except for MMSE performance in model 2). Models replacing APOE*4 status with BMI or current depression found no associations for these factors (S27 Table).

**Table 2:** Associations between putative risk factors and cognitive performance found with fully adjusted models^a^.

Factor	Model 1^b^				Model 2^c^			
	Global cognition		MMSE		Global cognition		MMSE	
	*B* (SE)	*I*^2^ (%)	*B* (SE)	*I*^2^ (%)	*B* (SE)	*I*^2^ (%)	*B* (SE)	*I*^2^ (%)
Age at baseline	−0.091 (0.012)***	56.2	−0.052 (0.007)***	35.5	−0.103 (0.013)***	35.2	−0.060 (0.009)***	26.7
Education	0.115 (0.021)***	85.7	0.095 (0.011)***	71.2	0.126 (0.021)***	72.2	0.106 (0.010)***	33.0
Sex, male	−0.014 (0.088)	59.7	−0.040 (0.091)	76.8	−0.087 (0.108)	55.5	−0.147 (0.127)	74.5
Alcohol								
1 drink/week^d^	0.077 (0.108)	0	0.160 (0.085)	0	0.080 (0.113)	0	0.148 (0.087)	0
2+ drinks/week^d^	0.081 (0.110)	54.5	0.069 (0.051)	4.6	0.064 (0.111)	52.6	0.063 (0.049)	0
APOE*4 carrier	−0.311 (0.107)**	48.2	−0.006 (0.055)	11.9	−0.251 (0.101)*	40.2	−0.078 (0.049)	0
Body mass index^e^					0.001 (0.007)	0	−0.002 (0.006)	23.1
Cardiovascular disease	0.030 (0.058)	0	0.095 (0.044)*	0	0.027 (0.062)	0	0.141 (0.047)**	0
Depression, current					−0.113 (0.057)*	0	−0.073 (0.069)	31.5
Diabetes	−0.230 (0.097)*	26.3	−0.119 (0.085)	45.8	−0.209 (0.116)	37.9	−0.136 (0.084)	34.5
High cholesterol	−0.034 (0.071)	23.8	−0.034 (0.036)	0	0.012 (0.051)	0	−0.050 (0.039)	0
Hypertension	−0.170 (0.115)	64.3	−0.007 (0.044)	7.0	−0.206 (0.122)	61.3	−0.022 (0.044)	0
Physical activity								
Moderate^f^					0.087 (0.093)	28.7	0.069 (0.078)	31.0
Vigorous^f^					0.160 (0.146)	56.9	0.168 (0.061)**	0
Smoking								
Past^g^	0.045 (0.069)	11.2	−0.025 (0.043)	0	0.074 (0.080)	23.0	−0.020 (0.043)	0
Current^g^	−0.144 (0.170)	34.7	−0.217 (0.082)**	0	−0.134 (0.167)	33.1	−0.202 (0.082)*	0
History of stroke	−0.252 (0.249)	65.9	−0.223 (0.089)*	4.5	−0.218 (0.270)	68.7	−0.199 (0.086)*	0

^a^Results are for the mean time in study (3.1 years) and controlled for age at baseline (mean = 73.1 years), education (mean = 9.15 years), and sex (40% male); negative *B* values indicate worse performance. Global cognition was calculated as a composite of 4 neuropsychological tests, each representing 1 of 4 cognitive domains: memory, language, processing speed, and executive functioning. MMSE denotes Mini-Mental State Examination. Empty cells are for factors not included in model 1.

*p < 0.05

**p < 0.01

***p < 0.001.

^b^Used data from 11 cohorts (*N* = 13,917) and included alcohol consumption, APOE*4 status, cardiovascular disease, diabetes, high cholesterol, hypertension, smoking, and history of stroke.

^c^Used data from 9 cohorts (*N* = 11,897) and included alcohol consumption, APOE*4 status, BMI, cardiovascular disease, depression, diabetes, high cholesterol, hypertension, physical activity, smoking, and history of stroke.

^d^Versus nil/minimal alcohol.

^e^Centered at mean = 25.2 kg/m^2^.

^f^One or more times/week versus minimal activity.

^g^Versus never smoked.

### Associations between factors and cognitive decline

Analyses with no risk factors in the model and pooled across cohorts revealed declining scores over time for the global cognition composite (*B* = −0.859, SE = 0.253, *p* < 0.01) and the MMSE (*B* = −0.452, SE = 0.168, *p* < 0.01; S25 Table). Quadratic effects show the rate of decline per decade becoming even greater as time in study progressed for the global cognition composite score (*B* = −0.049, SE = 0.023, *p* < 0.05).

As shown in Table 3, fully adjusted multivariable models found that higher age and APOE*4 carriage were both associated with more decline on both cognitive outcomes, and that diabetes was associated with more decline in MMSE score. Cardiovascular disease was associated with less cognitive decline. Heterogeneity of these associations was low (*I*^2^ values ranging between 0% and 11.1%), except for APOE*4 carriage, for which heterogeneity was moderate (*I*^2^ = 46.6%). Quadratic effects indicated that as time in study progressed, the slower rate of decline associated with high cholesterol became even slower, whereas the slower rate of decline associated with alcohol consumption weakened. The faster rate of decline associated with current smoking also diminished as time progressed (S28 Table). Heterogeneity of these associations was low (all *I*^2^ = 0). Models replacing APOE*4 status with BMI or current depression found no associations for these factors (S29 and S30 Tables).

**Table 3:** Effects of putative risk factors on the rate of cognitive decline found with fully adjusted models^a^.

Factor	Model 1^b^				Model 2^c^			
	Global cognition		MMSE		Global cognition		MMSE	
	*B* (SE)	*I*^2^ (%)	*B* (SE)	*I*^2^ (%)	*B* (SE)	*I*^2^ (%)	*B* (SE)	*I*^2^ (%)
Age at baseline	−0.065 (0.005)***	0	−0.046 (0.005)***	18.2	−0.067 (0.006)***	0	−0.051 (0.005)***	0
Education	−0.017 (0.011)	56.3	0.002 (0.008)	39.4	−0.024 (0.013)	62.9	0 (0.009)	39.7
Sex, male	0.016 (0.074)	37.7	0.025 (0.075)	50.0	0.003 (0.079)	38.0	−0.072 (0.048)	0
Alcohol								
1 drink/week^d^	0.254 (0.304)	57.8	0.121 (0.090)	0	0.137 (0.286)	49.8	0.155 (0.094)	0
2+ drinks/week^d^	0.120 (0.140)	43.1	0.009 (0.083)	28.6	0.055 (0.162)	49.9	−0.003 (0.09)	36.6
APOE*4 carrier	−0.339 (0.143)*	38.8	−0.103 (0.078)	24.3	−0.409 (0.177)*	49.6	−0.167 (0.077)*	17.5
Body mass index^e^					−0.014 (0.009)	0	0.003 (0.012)	52.8
Cardiovascular disease	0.140 (0.077)	0	0.156 (0.071)*	8.9	0.169 (0.083)*	0	0.182 (0.067)**	0
Depression, current					0.033 (0.220)	47.2	−0.03x (0.103)	30.8
Diabetes	−0.010 (0.100)	0	−0.128 (0.126)	45.7	−0.012 (0.108)	0	−0.185 (0.091)*	11.1
High cholesterol	0.074 (0.065)	0	−0.080 (0.052)	0	0.094 (0.070)	0	−0.048 (0.083)	30.8
Hypertension	−0.156 (0.177)	57.7	0.058 (0.051)	0	−0.114 (0.179)	46.9	0.065 (0.056)	0
Physical activity								
Moderate^f^					0.033 (0.096)	0	0.123 (0.124)	47.0
Vigorous^f^					0.039 (0.160)	26.6	0.058 (0.092)	13.7
Smoking								
Past^g^	−0.096 (0.073)	0	−0.007 (0.089)	40.1	−0.069 (0.085)	7.1	−0.009 (0.088)	39.5
Current^g^	−0.084 (0.220)	11.7	−0.090 (0.183)	46.4	−0.051 (0.244)	17.0	−0.090 (0.183)	46.4
History of stroke	−0.280 (0.177)	0	−0.136 (0.243)	62.2	−0.239 (0.217)	8.8	−0.278 (0.154)	20.4
Baseline score	−0.030 (0.050)	41.8	0.007 (0.046)	69.7	0.002 (0.036)	13.0	0.014 (0.039)	53.1

^a^Factor × time interactions for the mean time in study (3.1 y), controlled for age at baseline (mean = 73.1 y), education (mean = 9.15 y), and sex (40% female); negative *B* values indicate more decline. Global cognition was calculated as a composite of 4 neuropsychological tests, each representing 1 of 4 cognitive domains: memory, language, processing speed, and executive functioning. MMSE denotes Mini-Mental State Examination. Empty cells are for factors not included in model 1.

*p < 0.05

**p < 0.01

***p < 0.001.

^b^Used data from 11 cohorts (*N* = 13,917) and included alcohol consumption, APOE*4 status, cardiovascular disease, diabetes, high cholesterol, hypertension, smoking, and history of stroke.

^c^Used data from 9 cohorts (*N* = 11,897) and included alcohol consumption, APOE*4 status, BMI, cardiovascular disease, depression, diabetes, high cholesterol, hypertension, physical activity, smoking, and history of stroke.

^d^Versus nil/minimal alcohol.

^e^Centered at mean = 25.2 kg/m^2^.

^f^One or more times/week versus minimal activity.

^g^Versus never smoked.

Table 4 summarizes the significant linear associations between factors and cognitive performance and decline found with partially or fully adjusted multivariable models.

**Table 4:** Summary of factors significantly associated with worse or better cognitive performance and/or faster or slower cognitive decline^a^.

Factor	Performance	Decline
**Worse performance or faster decline**		
Age at baseline	**Global; MMSE**	**Global; MMSE**
Anxiety^b^	Global	
APOE*4 carriage	**Global**	**Global; MMSE**
Body mass index	Global	
Depression, current	**Global;** MMSE	
Diabetes	**Global;** MMSE	**MMSE**
Health, poor^b^^,^^c^	Global; MMSE	Global
Hypertension	Global	
Peripheral vascular disease	Global	
Smoking, current	**Global**	
Smoking, past		Global
Stroke	Global; **MMSE**	Global; MMSE
**Better performance or slower decline**		
Alcohol, 1 drink/week^d^	MMSE	
Alcohol, 2+ drinks/week^d^	MMSE	MMSE
Cardiovascular disease	**MMSE**	**Global; MMSE**
Education	**Global; MMSE**	
Physical activity, vigorous^e^	Global; **MMSE**	

^a^Significant effects for at least 1 fully adjusted multivariable model (independent effects) are shown in bold font, and significant effects for only partially adjusted multivariable models are in normal font. Empty cells indicate no significant results. Global cognition was calculated as a composite of 4 neuropsychological tests, each representing 1 of 4 cognitive domains: memory, language, processing speed, and executive functioning. MMSE denotes Mini-Mental State Examination.

^b^Not included in fully adjusted models.

^c^Versus very good.

^d^Versus nil/minimal alcohol.

^e^One or more times/week versus minimal activity.

### Comparisons of associations between white people and Asian people

Comparisons of associations between white people and Asian people were not performed for fully adjusted model 2 because this model retained only 1 Asian cohort. Significant results were found only with partially adjusted models.

**Fig 1:**
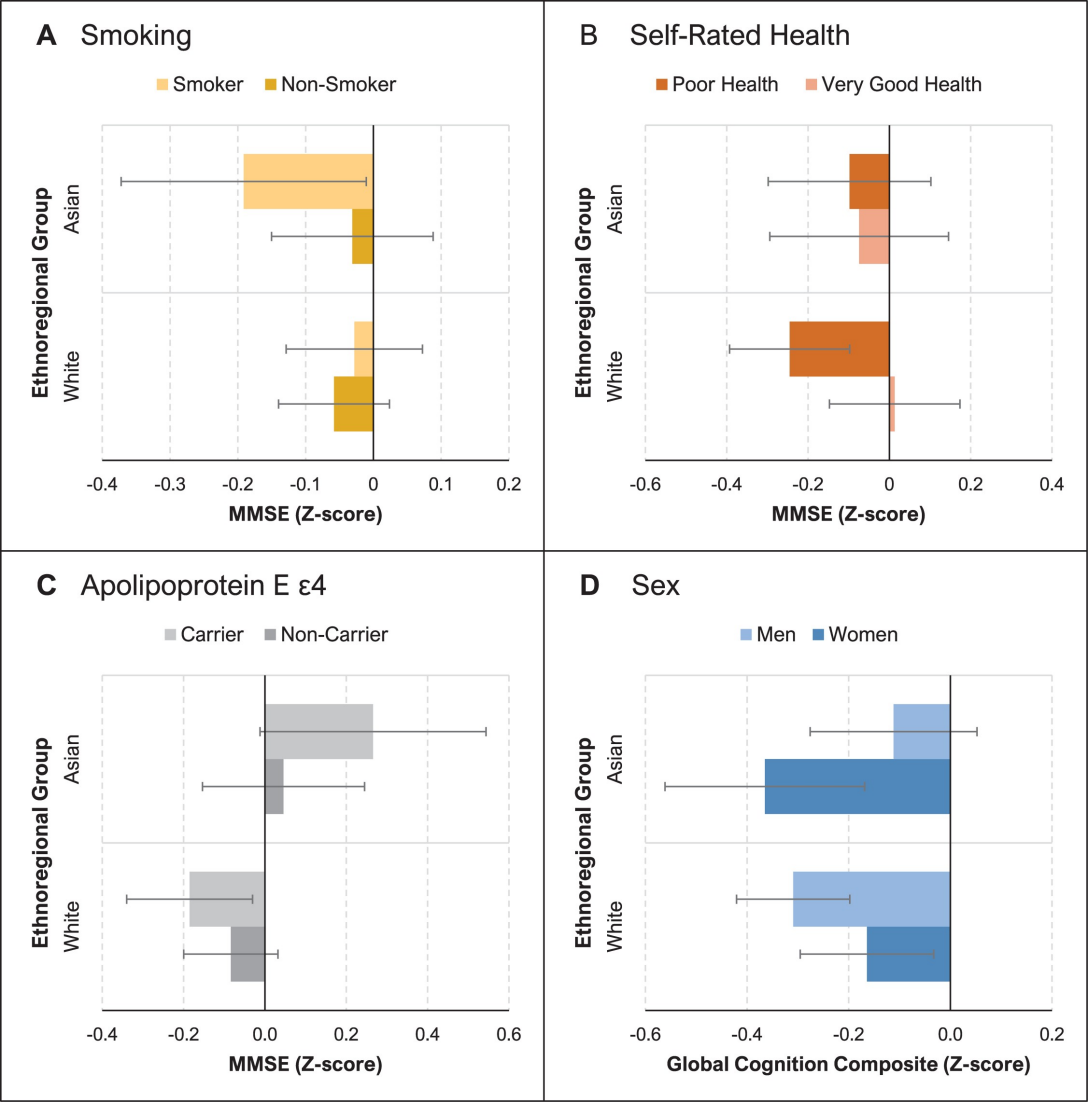
Risk factors for cognitive performance that differ between Asian people and white people. Bars show standardized scores at the mean time in study (3.1 years) for participants with and without the risk factor in both groups: Asian people and white people. Error bars indicate standard error of the mean. Mean Mini-Mental State Examination (MMSE) performance is compared for smokers and non-smokers (A), participants with poor versus very good self-rated health (B), and apolipoprotein E ε4 allele (APOE*4) carriers and non-carriers (C). (D) compares the mean performance for the global cognition composite (calculated from tests of 4 cognitive domains) in men and women.

Fig 1 shows significant differences in the association of risk factors with cognitive performance between white people and Asian people. Global cognition composite scores for men were lower than for women in white people but higher than for women in Asian people (group difference: *B* = 0.334, SE = 0.085, *p* < 0.001). Moderate heterogeneity for this effect (*I*^2^ = 54.9) suggests that the effect was not fully accounted for by ethno-regional differences. For the MMSE, APOE*4 carriers had higher scores than non-carriers in Asian people but not in white people (group difference: *B* = 0.343, SE = 0.146, *p* < 0.05). Poor self-rated health was associated with lower MMSE scores in white people but not in Asian people (group difference: *B* = 0.266, SE = 0.12, *p* < 0.05). Compared to white people, Asian people showed greater negative effects on MMSE scores associated with ever smoking (*B* = −0.240, SE = 0.118, *p* < 0.05). Heterogeneity for the effects of APOE*4 carriage, poor self-rated health, and smoking was low (*I*^2^ range = 0%–8.8%), suggesting that ethno-regional differences played a considerable role. For full results, including within-group effects, see S31 Table.

**Fig 2:**
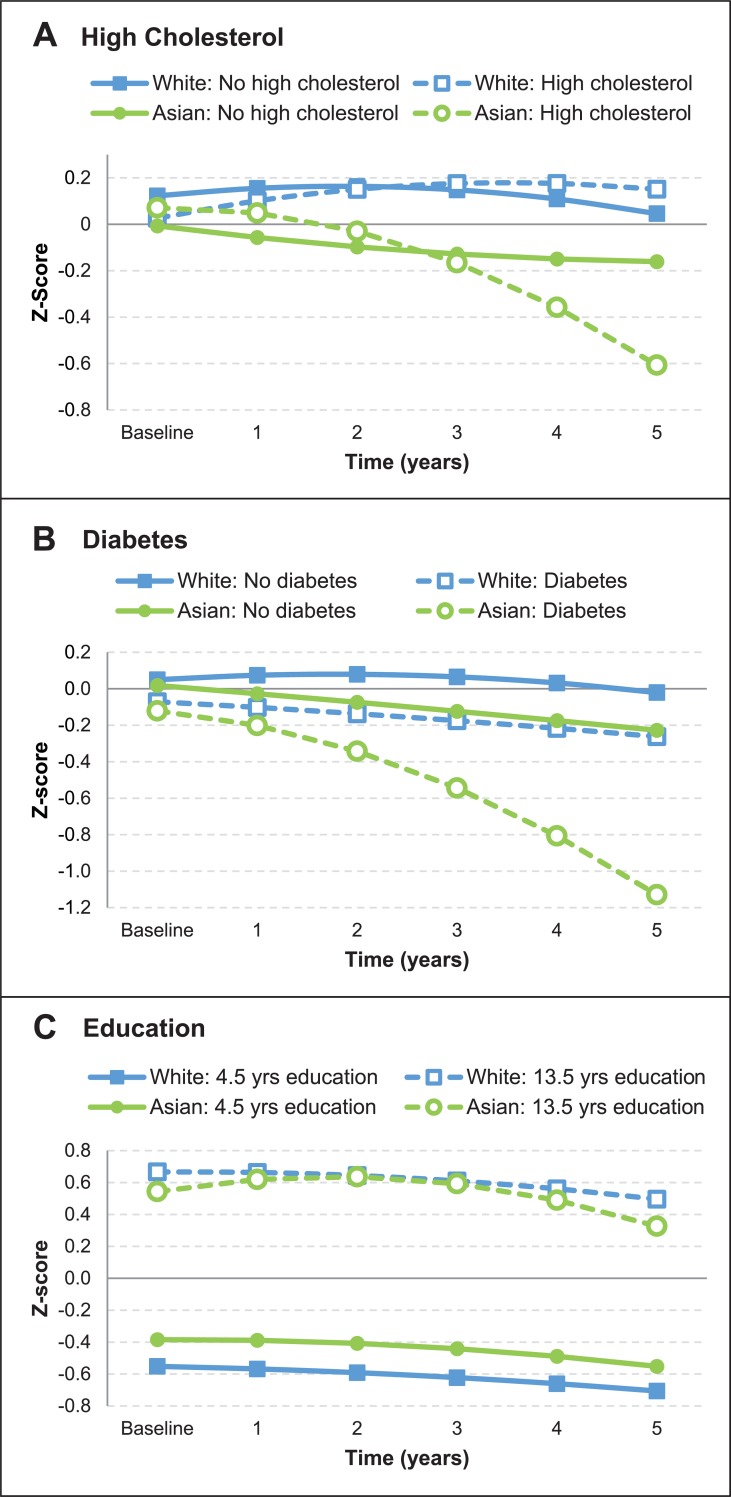
Risk factors for cognitive decline that differ between Asian people and white people. Fitted trajectories are for the first half decade since baseline, and show mean changes in standardized Mini-Mental State Examination (MMSE) scores for both groups: Asian people and white people. (A) compares participants with and without high cholesterol, and (B) compares participants with and without diabetes. (C) compares participants with high and low levels of education, determined as 1 standard deviation (4.5 years) above and below the mean of 9 years, respectively.

The overall rate of cognitive decline did not differ between white people and Asian people (see the results for models featuring no risk factors in S32 Table). However, the trajectories for MMSE scores associated with some factors differed significantly between white people and Asian people, as shown in Fig 2. There was greater decline associated with diabetes for Asian people than for white people (*B* = −0.658, SE = 0.27, *p* < 0.05; S32 Table). Further, quadratic effects showed that with increasing time in study, there was a growing rate of decline associated with each of diabetes (*B* = −0.296, SE = 0.137, *p* < 0.05), high cholesterol (*B* = −0.296, SE = 0.132, *p* < 0.05), and higher education (*B* = −0.024, SE = 0.011, *p* < 0.05) among Asian people compared to white people (S33 Table). Heterogeneity for each of these group differences was low (*I*^2^ < 10%), again suggesting a substantial influence of ethno-regional differences.

## Discussion

This large meta-analysis of over 48,000 individuals from 15 countries investigated associations between putative risk factors and late-life cognition. It has an additional 6 cohorts (including from 3 new countries) relative to our earlier study of cognitive decline in COSMIC cohorts [5], but more importantly includes a wide range of risk factors, beyond only those of age, sex, education, and APOE*4 carriage that we previously investigated. The current study found that higher age showed negative associations with performance and decline, in keeping with age being the strongest risk factor for dementia [33], and also for mild cognitive impairment [34]. Other factors independently associated with poorer performance were APOE*4 carriage, depression, diabetes, current smoking, and history of stroke. APOE*4 carriage and diabetes were also both independently associated with cognitive decline. Associations of cognitive performance and decline with APOE*4 reflect its being a risk factor for Alzheimer disease [35], and the corresponding moderate levels of heterogeneity are consistent with the relationship between APOE*4 and dementia being influenced by factors we did not control for in these analyses, such as race/ethnicity (which was controlled only in later analyses) and midlife vascular risk factors [36,37]. Evidence also links diabetes with poorer cognitive performance and greater decline [38], as well as increased risk of dementia [39]. The relationship between late-life depression and cognitive dysfunction is complex [40], but our finding of an association with performance but not decline might favor late-life depression being a psychological response to cognitive impairment or a prodrome of dementia, rather than a causal factor. Cognitive outcomes following stroke are varied [41], and our finding of stroke being independently associated with poorer performance but not decline suggests greater effects of stroke on initial impairment than on more gradual later changes in cognition. While implicated as a risk factor for cognitive decline and dementia [42], we found current smoking to be associated with a slowing of the rate of decline over time. This might reflect a protective effect of nicotine or smoking in late-life on cognition [43], or a selection bias arising from the death or dropout of smokers experiencing detrimental health or cognitive effects. Smokers had poorer cognitive performance than non-smokers, consistent with an overall negative cumulative impact of smoking on late-life cognition [44].

Education and vigorous physical activity were both independently associated with better cognitive performance. The association for education is well established [45], but our observation of high heterogeneity in the association between education and cognition across cohorts is consistent with a previously reported large degree of ethno-geographic variability in the association between education and dementia [46]. While existing evidence supports a positive effect of physical activity on cognition [47], we found that vigorous physical activity was associated with better cognition, but that moderate activity was not. However, we did not distinguish between moderate intensity activity types or consider duration or frequency beyond at least once a week. The association between physical activity and cognition was independent of potential mediating factors like BMI and cardiovascular disease, consistent with more direct neuroplastic effects [47]. Cardiovascular disease was associated with better cognitive performance and slower decline. This differs from reports of the opposite association [48] or of no association in individuals aged 65 years or more [49]. While this result remains unaccounted for, the true association may have been obscured by not controlling for the potentially protective effects of medications [50]. Our results for high cholesterol are consistent with this being associated with a lower risk of dementia [51] and less cognitive decline in late life [52].

Atrial fibrillation is often reported as a risk factor for cognitive decline [53], but this association was not observed in the current study. From another study, the risk of cognitive decline appears to be stronger with earlier age of onset and longer duration of atrial fibrillation [54], but we did not have age of onset data to gauge whether participants in our cohorts had atrial fibrillation long enough to elicit statistically significant effects. We also did not have the data needed to consider the use of anticoagulants, which a recent meta-analysis found significantly reduced the effects of atrial fibrillation on cognitive decline [55].

Global cognition composite scores for men were lower than for women among white people, but this was reversed in Asian people. Older women generally outperform older men on verbal memory tests, possibly because of biological mechanisms such as effects of estrogen [56] or sex-specific cognitive reserve [57], but also sociocultural factors [58]. Opposite effects have been attributed to gender disparities in educational opportunities and socioeconomic investment in some countries, including China [59]. Indeed, another study from China found better memory performance in women than men when levels of education were comparable [60]. Our observation of a moderate level of heterogeneity for ethno-regional differences in the association between sex and cognition supports the idea that factors like socioeconomics may be involved, but that these are not ethno-regionally specific. APOE*4 carriers had higher MMSE scores than non-carriers among Asian people, but not among white people. The low heterogeneity of this difference suggests that ethno-regional differences played a large role in accounting for the moderate level of between-study variability of this effect when investigated across all cohorts (Table 2). While some studies report better performance on particular cognitive tests among carriers [61], higher scores are not expected, given the association between APOE*4 and Alzheimer disease [35]. However, the prevalence of APOE*4 among individuals with Alzheimer disease is reportedly lower in Asian countries than in North America and Northern Europe [62]. This potentially suggests APOE*4 has less detrimental cognitive effects in Asian people, but does not explain better scores. APOE*4 is a strong predictor of mortality [63], and differences in this association between white people and Asian people that could generate different levels of survivor bias in these groups remain to be explored. Lower cognitive performance in white people with poor self-rated health is consistent with expectations [64]. However, Asian people with poor self-rated health did not show worse cognition, possibly because culturally based health perceptions led them to underestimate their true health status, as suggested by the finding that US residents of Asian ethnicity (~80% foreign-born) more often report poor or fair health than US-born white people, despite having objectively better health [65]. Ever smoking had larger negative effects on MMSE scores in Asian people than in white people. While this effect is potentially related to differences in the lifetime duration and intensity of smoking [44], we lack sufficient data to investigate this.

Compared to white people, there was both more cognitive decline and a growing rate of decline associated with diabetes in Asian people, who might develop diabetes younger and experience more complications than people of European descent [66]. We also found a growing rate of cognitive decline associated with high cholesterol in Asian people. A previous finding of faster cognitive decline in Chinese participants with high cholesterol was thought to potentially reflect ethnic/racial differences and/or a low use of statins, which may convey cognitive benefits [67]. Similar effects could help to explain our results, given a report of lower statin use among outpatients with atherothrombosis in Asian countries (including all that we investigated) than in North America, Western Europe, and Australia [68]. The cognitive reserve hypothesis suggests that education delays cognitive decline by offsetting the effects of accumulating neuropathology, but that decline is rapid once the level of neuropathology cannot be compensated for [69]. The growing rate of cognitive decline associated with increased education among Asian people compared to white people might be because white people had higher levels of education overall, and did not show an association between education and decline at the mean age of study because of greater cognitive reserve.

The results of this study show similarities with and differences from our earlier study using 14 COSMIC cohorts [5]. However, it is important to note that the focuses of the studies were slightly different, and the corresponding differences in the statistical approaches mean that the results from the 2 studies are not strictly comparable. Our earlier study focused on modeling the trajectory of variation of cognitive performance with age, combining both cross-sectional and longitudinal variation of cognition with age, whereas the current study made time in study the main predictor variable (and included age at baseline as a covariate). The latter approach distinguishes between the effects of cross-sectional and longitudinal variation of age, and is similar to that used by Piccinin et al. [70]. Faster decline with greater age, or with longer time in study, and APOE*4 carriage was found in both COSMIC studies, but only the earlier study found slightly slower decline on the MMSE with more education or being male, and slightly faster decline on the MMSE for Asian people than for white people. The addition of new cohorts and change in overall ethno-regional representation may have also contributed to these differences, as could have the slightly greater overall level of education of the participants in the current study (9.0 versus 8.8 years), given how education can affect decline via cognitive reserve as discussed above.

Our study has a number of limitations, including the broad classifications of people as white or Asian that overlook major ethnic and genetic diversity. We aim to expand the number of COSMIC members from particular countries to enable us to perform analyses with more refined groups, such as Chinese or Japanese rather than Asian people more broadly, in future investigations. Harmonization of data can involve some loss or distortion of information that may alter the nature of associations found. This may be particularly so for physical activity, where the nuances of questionnaires used by some studies were lost during harmonization to a simpler format that accommodated data from studies with less sophisticated measures. Other limitations include insufficient anxiety and general health data to investigate any independent associations of these factors with cognition. All of the factors we investigated have been previously linked to cognitive performance or decline, but so have others not included in the current study, including hearing loss and social isolation [71]. We intend to investigate these factors in the future, using different and smaller sets of COSMIC cohorts that have these data. Further, we did not specifically investigate factors at midlife, when some, such as high cholesterol, exert their strongest late-life cognitive effects [72]. The MMSE has been criticized as psychometrically unsound for assessing cognitive change in healthy older adults [73], and its having different properties from our global cognition composite may explain some of the different findings between these measures. Also, only using global measures of cognition means effects of factors associated with particular cognitive domains were not investigated. Finally, across all factors investigated, we found more to be associated with cognitive performance than with cognitive decline. This suggests that many of the associations may be due to common or confounding causes rather than the identified factors having causal effects on cognitive change in late life.

In this study, we identified factors with independent effects on late-life cognitive performance and decline. With data from so many diverse international cohorts, our results can be generalized on a global scale. Some of the factors we identified are modifiable, including education, smoking, physical activity, diabetes, and stroke. However, since this analysis cannot establish causality, the utility of targeting these factors in interventions to delay or minimize cognitive decline, including that leading to dementia, is unknown. It was recently reported that some dementia risk factors are more prevalent in low- and middle-income countries than in high-income countries, which contributes to them having greater population attributable fractions for dementia in those regions [74]. This, together with our finding that white people and Asian people showed different associations between some factors and cognition, suggests that interventions to reduce the risk and level of cognitive decline and dementia may require tailoring to particular ethno-regional groups. Further consideration of how interventions are implemented is likely to be particularly required in low- and middle-income countries, where resources may be limited and access to healthcare restricted [75].

## Supporting information

S1 Checklist STROBE statement checklist for cohort studies. (DOCX)

S1 Table Criteria used to exclude participants with dementia at baseline. (DOCX)

S2 Table Percentages of individuals missing age, sex, education, and dementia status data. (DOCX)

S3 Table Number of assessment waves, time since baseline, and number of individuals with MMSE scores for each wave. (DOCX)

S4 Table Ethics approvals for the individual contributing studies. (DOCX)

S5 Table Year values assigned to educational attainment categories. (DOCX)

S6 Table Global or screening test and neuropsychological tests used to represent the cognitive domains investigated. (DOCX)

S7 Table Risk factors analyzed for each study. (DOCX)

S8 Table Potential risk factors investigated: Definitions and numbers of studies contributing data. (DOCX)

S9 Table General health: Study-specific details and harmonization protocols. (DOCX)

S10 Table Anxiety: Study-specific details and harmonization protocols. (DOCX)

S11 Table Depression: Study-specific details and harmonization protocols. (DOCX)

S12 Table Hypertension: Study-specific details and harmonization protocols. (DOCX)

S13 Table Diabetes: Study-specific details and harmonization protocols. (DOCX)

S14 Table High cholesterol: Study-specific details and harmonization protocols. (DOCX)

S15 Table Peripheral vascular disease: Study-specific details and harmonization protocols. (DOCX)

S16 Table Atrial fibrillation: Study-specific details and harmonization protocols. (DOCX)

S17 Table Cardiovascular disease: Study-specific details and harmonization protocols. (DOCX)

S18 Table Stroke: Study-specific details and harmonization protocols. (DOCX)

S19 Table Alcohol use: Study-specific details and harmonization protocols. (DOCX)

S20 Table Physical activity: Study-specific details and harmonization protocols. (DOCX)

S21 Table Baseline demographic characteristics of the cohorts. (DOCX)

S22 Table Mean (SD) and number with data for continuous factors in each study. (DOCX)

S23 Table Prevalence of categorical factors in each study. (DOCX)

S24 Table Partially adjusted model results for effects of factors on cognitive performance and per decade cognitive decline. (DOCX)

S25 Table Coefficients for the time in study and time in study squared terms from partially and fully adjusted models. (DOCX)

S26 Table Quadratic effects: Coefficients from partially adjusted models showing association between each risk factor and the change in slope with each additional decade (time in study squared). (DOCX)

S27 Table Regression coefficients for association between each risk factor and cognitive performance from fully adjusted model 1 alternatives that replace APOE*4 status with body mass index or depression. (DOCX)

S28 Table Quadratic effects: Coefficients from fully adjusted models 1 and 2, showing association between each risk factor and the change in slope with each additional decade (time in study squared). (DOCX)

S29 Table Regression coefficients for association between each risk factor and change over time per decade from fully adjusted model 1 alternatives that replace APOE*4 status with body mass index or depression. (DOCX)

S30 Table Quadratic effects: Coefficients from the fully adjusted model 1 alternatives (that replace APOE*4 with either body mass index or current depression) showing associations between each risk factor and the change in slope with each additional decade (time in study squared). (DOCX)

S31 Table Comparisons of associations between factors and cognitive performance for white people and Asian people. (DOCX)

S32 Table Comparisons of associations between factors and per decade cognitive decline for white people and Asian people. (DOCX)

S33 Table Comparisons of associations between factors and per decade cognitive decline (quadratic effects) for white people and Asian people. (DOCX)

S1 Text Prospective analysis plan. (DOCX)

S2 Text Partially adjusted model results and discussion. (DOCX)

## Abbreviations

*APOE*4*: apolipoprotein E ε4 allele
BMI: body mass index
COSMIC: Cohort Studies of Memory in an International Consortium
IPD: individual participant data
MMSE: Mini-Mental State Examination
SD: standard deviation
SE: standard error
