# Supplementary material for: Determinants of cognitive performance and decline in 20 diverse ethno-regional groups: A COSMIC collaboration cohort study
Source: PLoS Med. 2019 Jul 23;16(7):e1002853. doi: 10.1371/journal.pmed.1002853 (PMC6650056; doi:10.1371/journal.pmed.1002853)
Supplement: S1 Checklist — (DOCX) [file pmed.1002853.s001.docx]

|  | **Item No** | **Recommendation** |
| --- | --- | --- |
| **Title and abstract** | 1 | (*a*) Indicate the study’s design with a commonly used term in the title or the abstract |
|  |  | Ttile |
|  |  | (*b*) Provide in the abstract an informative and balanced summary of what was done and what was found  Abstract |
| **Introduction** | | |
| Background/rationale | 2 | Explain the scientific background and rationale for the investigation being reported  Introduction: paragraph 1. |
| Objectives | 3 | State specific objectives, including any prespecified hypotheses  Introduction, paragraph 2. |
| **Methods** | | |
| Study design | 4 | Present key elements of study design early in the paper  Methods: Contributing studies section. |
| Setting | 5 | Describe the setting, locations, and relevant dates, including periods of recruitment, exposure, follow-up, and data collection  Methods: Table 1.  S3 Table. |
| Participants | 6 | (*a*) Give the eligibility criteria, and the sources and methods of selection of participants. Describe methods of follow-up  Methods: Contributing studies section.  Table 1.  S3 Table. |
|  |  | (*b*) For matched studies, give matching criteria and number of exposed and unexposed  NA |
| Variables | 7 | Clearly define all outcomes, exposures, predictors, potential confounders, and effect modifiers. Give diagnostic criteria, if applicable  Methods: Measures section.  S8-S20 Tables.  Methods: Statistical Analysis, Standardization of outcome scores section. |
| Data sources/ measurement | 8* | For each variable of interest, give sources of data and details of methods of assessment (measurement). Describe comparability of assessment methods if there is more than one group  Table 1.  Methods: Measures section.  S8-S20 Tables. |
| Bias | 9 | Describe any efforts to address potential sources of bias  Methods: Contributing studies section. |
| Study size | 10 | Explain how the study size was arrived at  Results: Sample description section. |
| Quantitative variables | 11 | Explain how quantitative variables were handled in the analyses. If applicable, describe which groupings were chosen and why  Methods: Statistical Analysis section. |
| Statistical methods | 12 | (*a*) Describe all statistical methods, including those used to control for confounding  Methods: Statistical Analysis section. |
|  |  | (*b*) Describe any methods used to examine subgroups and interactions  Methods: Statistical Analysis, Ethno-regional comparisons section. |
|  |  | (*c*) Explain how missing data were addressed  Methods: Statistical Analysis, Associations of age at baseline, sex and education with cognitive performance/decline section |
|  |  | (*d*) If applicable, explain how loss to follow-up was addressed  Methods: Statistical Analysis, Associations of age at baseline, sex and education with cognitive performance/decline section |
|  |  | (*e*) Describe any sensitivity analyses  NA |
| **Results** | | |
| Participants | 13* | (a) Report numbers of individuals at each stage of study—eg numbers potentially eligible, examined for eligibility, confirmed eligible, included in the study, completing follow-up, and analysed  S2 and S3 Tables. |
|  |  | (b) Give reasons for non-participation at each stage  With 20 different contributing studies and varying numbers of assessment waves, this level of information is not appropriate here, though can be found via the references for the studies listed in Table 1. |
|  |  | (c) Consider use of a flow diagram  Twenty different contributing studies and varying numbers of assessment waves preclude the use of a manageable flow diagram. |
| Descriptive data | 14* | (a) Give characteristics of study participants (eg demographic, clinical, social) and information on exposures and potential confounders  S21-S23 Tables. |
|  |  | (b) Indicate number of participants with missing data for each variable of interest  S2 and S20-S22 Tables. |
|  |  | (c) Summarise follow-up time (eg, average and total amount)  S3 Table. |
| Outcome data | 15* | Report numbers of outcome events or summary measures over time  S25 Table. |
| Main results | 16 | (*a*) Give unadjusted estimates and, if applicable, confounder-adjusted estimates and their precision (eg, 95% confidence interval). Make clear which confounders were adjusted for and why they were included  Methods: Statistical Analysis section. |
|  |  | (*b*) Report category boundaries when continuous variables were categorized  Methods: measures section.  S8-S20 Tables. |
|  |  | (*c*) If relevant, consider translating estimates of relative risk into absolute risk for a meaningful time period  NA |
| Other analyses | 17 | Report other analyses done—eg analyses of subgroups and interactions, and sensitivity analyses  Results: Ethno-regional comparisons section.  Figures 1 and 2.  S31-S33 Tables. |
| **Discussion** | | |
| Key results | 18 | Summarise key results with reference to study objectives  Table 4. |
| Limitations | 19 | Discuss limitations of the study, taking into account sources of potential bias or imprecision. Discuss both direction and magnitude of any potential bias  Discussion: second last paragraph. |
| Interpretation | 20 | Give a cautious overall interpretation of results considering objectives, limitations, multiplicity of analyses, results from similar studies, and other relevant evidence  Discussion: final paragraph. |
| Generalisability | 21 | Discuss the generalisability (external validity) of the study results  Discussion: final paragraph. |
| **Other information** | | |
| Funding | 22 | Give the source of funding and the role of the funders for the present study and, if applicable, for the original study on which the present article is based  Statement provided during web-based submission. |
