# Supplementary material for: Determinants of cognitive performance and decline in 20 diverse ethno-regional groups: A COSMIC collaboration cohort study
Source: PLoS Med. 2019 Jul 23;16(7):e1002853. doi: 10.1371/journal.pmed.1002853 (PMC6650056; doi:10.1371/journal.pmed.1002853)
Supplement: S1 Table — (DOCX) [file pmed.1002853.s002.docx]

| **Study** | **Criteria** |
| --- | --- |
| Bambui | MMSE score cut-off point 13/14 appropriate for Brazilian populations with low schooling^a^ |
| CFAS | AGECAT organicity level of O3 |
| CHAS | DSM-IV or education-adjusted 10/66 Lancet dementia diagnosis; those with CDR>=1 but not indicated as having a dementia diagnosis were also excluded |
| EAS | DSM-IV |
| ESPRIT | Standardized interview by a neurologist incorporating cognitive testing, with diagnoses validated by an independent panel of expert neurologists |
| HELIAD | DSM-IV |
| HK-MAPS | Clinical Dementia Rating ≥1 |
| Invece.Ab | DSM-IV |
| KLOSCAD | DSM-IV |
| LEILA75+ | DSM-IV |
| MAAS | MMSE score <24 |
| MoVIES | Clinical Dementia Rating ≥1 |
| PATH | DSM-IV |
| SALSA | California ADDTC criteria for vascular dementia and NINDS-ADRDA for Alzheimer’s disease |
| SGS | Self-reported medical history |
| SLASI | DSM-IV |
| SPAH | DSM-IV |
| Sydney MAS | DSM-IV |
| Tajiri | Clinical Dementia Rating ≥1, with DSM-IV follow-up |
| ZARADEMP | DSM-IV |

# ^a^ Castro-Costa E, Fuzikawa C, Uchoa E, Firmo JO, Lima-Costa MF. Norms for the mini-mental state examination: adjustment of the cut-off point in population-based studies (evidences from the Bambui health aging study). Arq Neuropsiquiatr 2008;66:524-8.
