# Supplementary material for: Determinants of cognitive performance and decline in 20 diverse ethno-regional groups: A COSMIC collaboration cohort study
Source: PLoS Med. 2019 Jul 23;16(7):e1002853. doi: 10.1371/journal.pmed.1002853 (PMC6650056; doi:10.1371/journal.pmed.1002853)
Supplement: S2 Table — (DOCX) [file pmed.1002853.s003.docx]

| **Study** | **Missing (%)** | **Original N** |
| --- | --- | --- |
| Bambui | 3.1 | 1606 |
| CFAS | 2.9 | 13004 |
| CHAS | 0.5 | 2944 |
| EAS | 2.9 | 2255 |
| ESPRIT | 0.1 | 2259 |
| HELIAD | 2.9 | 1258 |
| HK-MAPS | 0.6 | 790 |
| Invece.Ab | 1.1 | 1321 |
| KLOSCAD | 0.0 | 6833 |
| LEILA75+ | 2.3 | 1265 |
| MAAS | 0.1 | 806^a^ |
| MoVIES | 0.0 | 1681 |
| PATH | 0.2 | 2551 |
| SALSA | 0.6 | 1789 |
| SGS | 0.9 | 2206 |
| SLASI | 71.0^b^ | 2804 |
| SPAH | 5.6 | 2072 |
| Sydney MAS | 0.0 | 1037 |
| Tajiri | 0.0 | 100 |
| ZARADEMP | 1.2 | 4803 |

^a^ After excluding participants aged less than 55 years.

# ^b^ Primarily due to missing education data (69.5%).
