# Supplementary material for: Determinants of cognitive performance and decline in 20 diverse ethno-regional groups: A COSMIC collaboration cohort study
Source: PLoS Med. 2019 Jul 23;16(7):e1002853. doi: 10.1371/journal.pmed.1002853 (PMC6650056; doi:10.1371/journal.pmed.1002853)
Supplement: S3 Table — (DOCX) [file pmed.1002853.s004.docx]

| **Study** | **Waves**^a^ | **Baseline no.** | | **Wave 2** | | **Wave 3** | | | **Wave 4** | | **Wave 5** | | **Wave 6** | | **Wave 7** | |
| --- | --- | --- | --- | --- | --- | --- | --- | --- | --- | --- | --- | --- | --- | --- | --- | --- |
| Bambui | 16 | 1491 | | 1; 1372 | | 2; 1288 | | | 3; 1220 | | 4; 1146 | | 5; 1092 | | 6; 1013 | |
| CFAS^b^ | 3 | 12031 | | 2.1±0.2; 8275 | | 9.9±0.6; 3056 | | |  | |  | |  | |  | |
| CHAS | 2 | 2554 | | 4.6±0.9; 1792 | |  | | |  | |  | |  | |  | |
| EAS | 16 | 2062 | | 1.2±0.5; 1275 | | 2.4±0.7; 922 | | | 3.5±0.8; 696 | | 4.6±0.9; 544 | | 5.6±1.0; 432 | | 6.6±1.1; 324 | |
| ESPRIT | 4 | 2168 | | 1.7±0.2; 1896 | | 3.8±0.2; 1638 | | | 7.6±0.2; 1235 | |  | |  | |  | |
| HELIAD | 2 | 1052 | | 2.8±0.6; 461 | |  | | |  | |  | |  | |  | |
| HK-MAPS | 3 | 751 | | 1.9±0.4; 523 | | 5.4±0.3; 447 | | |  | |  | |  | |  | |
| Invece.Ab | 2 | 1170 | | 2.2±0.2; 1020 | |  | | |  | |  | |  | |  | |
| KLOSCAD | 2 | 6442 | | 2.0±0.3; 4922 | |  | | |  | |  | |  | |  | |
| LEILA75+ | 7 | 1040 | | 1.6±0.2; 785 | | 3.0±0.1; 655 | | | 4.5±0.1; 485 | | 6.1±0.2; 351 | | 7.1±0.3; 312 | | 14.9±0.5; 50 | |
| MAAS | 3 | 796 | | 6.2±0.2; 553 | | 12.5±0.3; 396 | | |  | |  | |  | |  | |
| MoVIES | 7 | 1610 | | 2.0±0.2; 1298 | | 4.2±0.4; 1135 | | | 6.6±0.5; 1001 | | 8.9±0.6; 826 | | 11.3±0.6; 651 | | 13.4±0.5; 299 | |
| PATH | 3 | 2542 | | 4.1±0.9; 2184 | | 8.1±0.3; 1917 | | |  | |  | |  | |  | |
| SALSA | 7 | 1710 | | 1.1±0.2; 1258 | | 2.2±0.2; 1152 | | | 3.4±0.4; 1106 | | 5.4±0.6; 961 | | 6.8±0.5; 849 | | 8.1±0.5; 765 | |
| SGS | 2 | 2087 | | 2.0±0.0; 854 | |  | | |  | |  | |  | |  | |
| SLASI | 3 | 792 | | 1.6±0.5; 538 | | 3.9±0.2; 301 | | |  | |  | |  | |  | |
| SPAH | 2 | 1824 | | 2.2±0.3; 1483 | |  | | |  | |  | |  | |  | |
| Sydney MAS | 4 | 1037 | | 1.9±0.1; 884 | | 4.0±0.2; 773 | | | 5.9±0.2; 672 | |  | |  | |  | |
| Tajiri | 2 | 100 | | 5; 98 | |  | | |  | |  | |  | |  | |
| ZARADEMP | 3 | 4542 | | 2.2±0.2; 3214 | | 4.7±0.4; 2390 | | |  | |  | |  | |  | |
| *Continuation of waves 8-16 for Bambui and EAS* | | | | | | | | | | | | | | | | |
| **Study** | **Wave** **8** | | **Wave 9** | | **Wave 10** | | **Wave 11** | **Wave 12** | | **Wave 13** | | **Wave 14** | | **Wave 15** | | **Wave 16** |
| Bambui | 7; 1032 | | 8; 875 | | 9; 809 | | 10; 716 | 11; 700 | | 12; 607 | | 13; 592 | | 14; 441 | | 15; 380 |
| EAS | 7.6±1.0; 238 | | 8.7±1.2; 150 | | 9.8±1.2; 104 | | 10.8±1.3; 77 | 11.6±0.8; 64 | | 12.7±0.8; 50 | | 13.6±1.0; 30 | | 14.5±0.4; 7 | | 15.1±0.0; 2 |

Note: Values are in mean±SD years. Numbers for Mini-Mental State Examination (MMSE) data are not always the same as the numbers for other cognitive test data.

^a^ Includes baseline.

^b^ The number and type of follow-up assessments differed among the participants (see http://www.cfas.ac.uk/cfas-i/cfasistudy-design/), and we used an abridged schedule comprising baseline and two follow-up waves that captured the majority of the sample (waves S0, C2/S2, CX).
