# Supplementary material for: Determinants of cognitive performance and decline in 20 diverse ethno-regional groups: A COSMIC collaboration cohort study
Source: PLoS Med. 2019 Jul 23;16(7):e1002853. doi: 10.1371/journal.pmed.1002853 (PMC6650056; doi:10.1371/journal.pmed.1002853)
Supplement: S4 Table — (DOCX) [file pmed.1002853.s005.docx]

| **Study** | **Institutional Review Board** |
| --- | --- |
| Bambui | Ethics Boards of the Fundac¸a˜o Oswaldo Cruz in Rio de Janeiro and the Instituto Rene´ Rachou of the Fundac¸a˜o Oswaldo Cruz in Belo Horizonte, Brazil (14/2007 - CEPSH-CpqRR) |
| CFAS | Anglia and Oxford Multi-centre Research Ethics Committee (MREC) - 99/5/22; Eastern MREC – 99/5/22; Eastern MREC – 05/MREO5/37; NRES Committee East of England – 05/MRE05/37 |
| CHAS | Medical University of Havana’s Ethics Committee – Approval 20/01/2003 |
| EAS | Albert Einstein College of Medicine Institutional Review Board (Approval#1996-175) |
| ESPRIT | Ethics committee (CCPPRB) of the Kremlin Bicetre hospital (n° registered 99-28) |
| HELIAD | Institutional Ethics Review Board of the University of Thessaly (ΒΕΥ846Ψ8Ν2-32Π) |
| HK-MAPS | Joint Chinese University of Hong Kong-New Territories East Cluster Clinical Research Ethics Committee (CRE-2011.101) |
| Invece.Ab | Ethics Committee of the University of Pavia (#3/2009) |
| KLOSCAD | Institutional Review Board of Seoul National University Bundang Hospital, Korea (IRB No. B-0912/089-010) |
| LEILA75+ | Ethics committee of the University of Leipzig (C7 79934700) |
| MAAS | Ethics committee of Maastricht University Medical Centre (MEC05-107) |
| MoVIES | University of Pittsburgh Institutional Review Board (IRB# 961263-0110) |
| PATH | Australian National University Human Research Ethics Committee (#M9807, #2002/189, #2006/314, # 2010/542, #2001/2, #2009/039) |
| SALSA | University of California, San Francisco Human Research Protection Program Institutional Review Board (IRB#10-00243) |
| SGS | Institutional Review Board of the Institute of Health Science, Kyushu University (IHS-2010-22) |
| SLASI | National University of Singapore Institutional Review Board (Reference Code: 04-140) |
| SPAH | Ethical Committee for the Analysis of Research Projects (CAPesq) - Hospital das Clínicas and Medical School - Project Registry Number: 257/2002; National Ethical Committee on Research (CONEP-Brazil) - Project Registry Number: 4355 |
| Sydney MAS | University of New South Wales Human Research Ethics Committee (approval #14327) |
| Tajiri | Ethical Committee of Tohoku University Graduate School of Medicine (#2012276, #2014160, #20141238, and #20141767) |
| ZARADEMP | Ethics committee of the Zaragoza University Hospital (CEICA # CP16/2012) |

Written consent was exclusively or predominantly obtained from participants in all studies (SPAH obtained oral consent from illiterate participants; CFAS obtained oral consent, countersigned by a witness, from participants with a physical/visual disability).

Further participant consent was not deemed necessary as only fully de-identified data were shared with the analysis team (e.g., as per the Privacy Rule proposed by the National Institute of Health, USA: http://privacyruleandresearch.nih.gov/research_repositories.asp).
