# Supplementary material for: Determinants of cognitive performance and decline in 20 diverse ethno-regional groups: A COSMIC collaboration cohort study
Source: PLoS Med. 2019 Jul 23;16(7):e1002853. doi: 10.1371/journal.pmed.1002853 (PMC6650056; doi:10.1371/journal.pmed.1002853)
Supplement: S5 Table — (DOCX) [file pmed.1002853.s006.docx]

| **Study** | **Category** | **Years** |
| --- | --- | --- |
| CHAS | None | 0 |
|  | Some, did not complete primary | 5 |
|  | Complete primary | 7 |
|  | Completed secondary | 10.5 |
|  | Tertiary college – University degree | 18 |
| ESPRIT | None | 0 |
|  | Primary | 5 |
|  | 1st to 4th year senior school/higher primary | 9 |
|  | Short technical or professional | 11 |
|  | 5th year to upper 6th form | 12 |
|  | Long technical or professional | 12 |
|  | Higher education including higher technical education | 15 |
| MAAS | Elementary education | 6 |
|  | Lower vocational education | 9 |
|  | Intermediate secondary education | 9 |
|  | Intermediate vocational education | 10 |
|  | Higher secondary education | 11 |
|  | Higher vocational education | 15 |
|  | University education | 16 |
|  | Scientific education | 17 |
| MoVIES | Graduate/professional | 17.17 |
|  | College graduate | 15.81 |
|  | Partial college | 13.62 |
|  | Trade/technical | 11.50 |
|  | High school graduate | 11.98 |
|  | Partial high school | 10.39 |
|  | 6^th^-9^th^ grade | 7.98 |
|  | < 6^th^ grade | 3.75 |

Year values for the categories provided by CHAS, ESPRIT and MAAS were based on local knowledge of the education system. MoVIES provided categorical education data for all participants, as well as year values for 79%, and these data were used to calculate a mean year value for each category that was assigned to individuals missing year data.
