# Supplementary material for: Determinants of cognitive performance and decline in 20 diverse ethno-regional groups: A COSMIC collaboration cohort study
Source: PLoS Med. 2019 Jul 23;16(7):e1002853. doi: 10.1371/journal.pmed.1002853 (PMC6650056; doi:10.1371/journal.pmed.1002853)
Supplement: S6 Table — (DOCX) [file pmed.1002853.s007.docx]

| Study | Global test | Memory | Language^a^ | Processing speed^b^ | Executive functioning^b^ |
| --- | --- | --- | --- | --- | --- |
| Bambui | MMSE | MMSE 3-word list recall | - | - | - |
| CFAS | MMSE | MMSE 3-word list recall | Animals | - | - |
| CHAS | CSI-D^c^ | CERAD 10-word list recall test | Animals | - | - |
| EAS | Blessed Information Memory Concentration test^d^ | Free and Cued Selective Reminding Test | Animals | TMTA | TMTB (=300s if TMTA≥0 and TMTB missing or >300) |
| ESPRIT | MMSE | MMSE 3-word list recall | Animals (30s) | TMTA | TMTB |
| HELIAD | MMSE | Greek Verbal Learning Test | Objects | TMTA | TMTB |
| HK-MAPS | MMSE | ADAS-Cog delayed recall item | Animals | TMTA (one set each of Arabic and Chinese numbers; 120s) | TMTB (alternating Arabic and Chinese numbers; 120s) |
| Invece.Ab | MMSE | RAVLT trial 7 (15 min delay) | Mean of Colours, Animals, Fruits, Cities (each 120s) | TMTA | TMTB |
| KLOSCAD | MMSE | CERAD 10-word list recall test | Animals | TMTA (360s) | TMTB (360s) |
| LEILA75+ | MMSE | MMSE 3-word list recall | - | - | - |
| MAAS | MMSE | RAVLT trial 7 | Animals | - | - |
| MoVIES | MMSE | CERAD 10-word list recall test | Animals | TMTA (300s) | TMTB (300s) |
| PATH | MMSE | California Verbal Learning Test (recall of first list) | - | TMTA (300s)^e^ | TMTB (300s)^e^ |
| SALSA | Modified MMSE^c^ | Spanish and English Verbal Learning Test | - | - | - |
| SGS | MMSE | MMSE 3-word list recall | - | - | - |
| SLASI | MMSE | RAVLT trial 7 | Animals | TMTA | TMTB |
| SPAH | CSI-D^c^ | CSI-D item #26 | Animals | - | - |
| Sydney MAS | MMSE | RAVLT trial 7 | Animals | TMTA | TMTB |
| Tajiri | MMSE | MMSE 3-word list recall^f^ | - | - | - |
| ZARADEMP | MMSE | MMSE 3-word list recall | - | - | - |

ADAS-Cog, Alzheimer’s Disease Assessment Scale – Cognitive; CERAD, Consortium to Establish a Registry for Alzheimer’s Disease neuropsychological assessment battery; CSI-D, Community Screening Instrument for Dementia; MMSE, Mini-Mental State Examination; RAVLT, Rey Auditory Verbal Learning Test

^a^ Semantic fluency test: category words generated in 60s unless otherwise indicated.

^b^ Time limits were not imposed except where indicated.

^c^ Converted to MMSE scores using a published co-calibration table: Crane PK, Narasimhalu K, Gibbons LE, et al. Item response theory facilitated cocalibrating cognitive tests and reduced bias in estimated rates of decline. J Clin Epidemiol 2008;61:1018-27 e9.

^d^ A validated formula was used to convert to MMSE scores: Thal LJ, Grundman M, Golden R. Alzheimer's disease: a correlational analysis of the Blessed Information-Memory-Concentration Test and the Mini-Mental State Exam. Neurology 1986;36:262-4.

^e^ Not administered at baseline.

^f^ Baseline only.
