# Supplementary material for: Determinants of cognitive performance and decline in 20 diverse ethno-regional groups: A COSMIC collaboration cohort study
Source: PLoS Med. 2019 Jul 23;16(7):e1002853. doi: 10.1371/journal.pmed.1002853 (PMC6650056; doi:10.1371/journal.pmed.1002853)
Supplement: S7 Table — (DOCX) [file pmed.1002853.s008.docx]

|  | Bambui | CFAS | CHAS | EAS | ESPRIT | HELIAD | HK-MAPS | Invece.Ab | KLOSCAD | LEILA | MAAS | MoVIES | PATH | SALSA | SGS | SLAS | SPAH | Sydney MAS | Tajiri^a^ | ZARADEMP |
| --- | --- | --- | --- | --- | --- | --- | --- | --- | --- | --- | --- | --- | --- | --- | --- | --- | --- | --- | --- | --- |
| *APOE*4* status |  |  |  |  |  |  |  |  |  |  |  |  |  |  |  |  |  |  |  |  |
| Body mass index |  |  |  |  |  |  |  |  |  |  |  |  |  |  |  |  |  |  |  |  |
| General health |  |  |  |  |  |  |  |  |  |  |  |  |  |  |  |  |  |  |  |  |
| Anxiety |  |  |  |  |  |  |  |  |  |  |  |  |  |  |  |  |  |  |  |  |
| Depression |  |  |  |  |  |  |  |  |  |  |  |  |  |  |  |  |  |  |  |  |
| History of depression |  |  |  |  |  |  |  |  |  |  |  |  |  |  |  |  |  |  |  |  |
| Hypertension |  |  |  |  |  |  |  |  |  |  |  |  |  |  |  |  |  |  |  |  |
| Blood pressure |  |  |  |  |  |  |  |  |  |  |  |  |  |  |  |  |  |  |  |  |
| Pulse pressure |  |  |  |  |  |  |  |  |  |  |  |  |  |  |  |  |  |  |  |  |
| Diabetes |  |  |  |  |  |  |  |  |  |  |  |  |  |  |  |  |  |  |  |  |
| High cholesterol |  |  |  |  |  |  |  |  |  |  |  |  |  |  |  |  |  |  |  |  |
| Peripheral vascular disease |  |  |  |  |  |  |  |  |  |  |  |  |  |  |  |  |  |  |  |  |
| Atrial fibrillation |  |  |  |  |  |  |  |  |  |  |  |  |  |  |  |  |  |  |  |  |
| Cardiovascular disease |  |  |  |  |  |  |  |  |  |  |  |  |  |  |  |  |  |  |  |  |
| Stroke |  |  |  |  |  |  |  |  |  |  |  |  |  |  |  |  |  |  |  |  |
| Smoking |  |  |  |  |  |  |  |  |  |  |  |  |  |  |  |  |  |  |  |  |
| Alcohol use |  |  |  |  |  |  |  |  |  |  |  |  |  |  |  |  |  |  |  |  |
| Physical activity |  |  |  |  |  |  |  |  |  |  |  |  |  |  |  |  |  |  |  |  |

Note: The risk factors analysed for each study are shown as shaded cells.

^a^ Data for atrial fibrillation, cardiovascular disease and stroke were also available for Tajiri, but models containing these did not converge.
