# Supplementary material for: Determinants of cognitive performance and decline in 20 diverse ethno-regional groups: A COSMIC collaboration cohort study
Source: PLoS Med. 2019 Jul 23;16(7):e1002853. doi: 10.1371/journal.pmed.1002853 (PMC6650056; doi:10.1371/journal.pmed.1002853)
Supplement: S8 Table — (DOCX) [file pmed.1002853.s009.docx]

| **Factor** | **Definition and/or typical derivation** | **Studies** | **Details** |
| --- | --- | --- | --- |
| *APOE*4* status | Carrier (1 or 2 e4 alleles) or non-carrier (no e4 alleles) | 16 | - |
| Body mass index | Calculated as weight/(height squared), with these typically measured (self-reported in ESPRIT and PATH) | 16 | - |
| General health | A self-response question like “In general, would you say your health is excellent, very good, good, fair, or poor?”, harmonized into three categories: Very good, Good, and Poor | 13 | S9 Table |
| Anxiety | Any of diagnosis, exceeding scale score cut-off, or taking medication for anxiety | 12 | S10 Table |
| Depression | Any of diagnosis, exceeding scale score cut-off, or taking medication for depression | 17 | S11 Table |
| History of depression | A previous diagnosis | 8 | S11 Table |
| Hypertension | Any of systolic blood pressure ≥140 mmHg, diastolic blood pressure ≥90 mmHg, taking medication for hypertension, or medical history | 20 | S12 Table |
| Blood pressure | Systolic and diastolic blood pressure (each an average of seated readings) | 14 | - |
| Pulse pressure | Calculated as systolic blood pressure minus diastolic blood pressure | 14 | - |
| Diabetes | Any of fasting blood glucose ≥126 mg/dL (>7 mmol/L), treatment for diabetes, or medical history | 20 | S13 Table |
| High cholesterol | Any of total cholesterol ≥240 mg/dL (>6.2 mmol/L), triglycerides ≥200 mg/dL (>2.3 mmol/L), treatment for high cholesterol, or medical history | 15 | S14 Table |
| Peripheral vascular disease | Intermittent claudication | 10 | S15 Table |
| Atrial fibrillation | Medical history (excludes studies with unspecified arrhythmia or conflating arrhythmia and atrial fibrillation) | 5 | S16 Table |
| Cardiovascular disease | History of any relevant condition (heart attack, angina, cardiomyopathy, valve disease, arrhythmia, atrial fibrillation, etc.) | 18 | S17 Table |
| Stroke | History of stroke or transient ischemic attack | 18 | S18 Table |
| Smoking | Past or current smoking (except for HK-MAPS and SPAH where only current smoking data were available) | 19 | - |
| Alcohol use | Standardised as number of alcoholic drinks/week, and categorised as Nil/minimal, At least one drink/week, or Two or more drinks/week | 13 | S19 Table |
| Physical activity | Activities were coded as light, moderate or vigorous according to published guidelines,^a^ with the activity of individuals categorised as Minimal (sedentary or only light activity), Moderate (at least once per week), Vigorous (at least once per week). | 10 | S20 Table |

# ^a^ Ainsworth BE, Haskell WL, Herrmann SD, et al. 2011 Compendium of Physical Activities: a second update of codes and MET values. Med Sci Sports Exerc 2011;43:1575-81; Smith L, Gardner B, Fisher A, Hamer M. Patterns and correlates of physical activity behaviour over 10 years in older adults: prospective analyses from the English Longitudinal Study of Ageing. BMJ Open 2015;5:e00742.
