# Supplementary material for: Determinants of cognitive performance and decline in 20 diverse ethno-regional groups: A COSMIC collaboration cohort study
Source: PLoS Med. 2019 Jul 23;16(7):e1002853. doi: 10.1371/journal.pmed.1002853 (PMC6650056; doi:10.1371/journal.pmed.1002853)
Supplement: S9 Table — (DOCX) [file pmed.1002853.s010.docx]

| **Study** | **Coding of original response options to Very good = 1, Good = 2, Poor = 3** |
| --- | --- |
| Bambui | Very Good, Good = 1; Reasonable = 2; Fair = 3 |
| CFAS | Excellent = 1; Good = 2; Fair, Poor = 3 |
| EAS | Excellent, Very Good = 1; Good = 2; Fair, Poor = 3 |
| HK-MAPS | CIRS sum of various organ system severity ratings: 0,1=1; 2-4=2; 5-13=3 |
| Invece.Ab | Visual analogue scale: 0-6 = 1; 7-8 = 2; 9-10 = 1 |
| KLOSCAD | Excellent, Good = 1; Fair = 2; Poor = 3 |
| LEILA75+ | Very Good/Excellent = 1; Good = 1; Fair = 2; Poor = 3; Very Poor = 3 |
| MoVIES | Excellent = 1; Good = 2; Fair, Poor = 3 |
| PATH | Excellent, Very Good = 1; Good = 2; Fair, Poor = 3 |
| SALSA | Excellent, Very Good = 1; Good = 2; Fair, Poor = 3 |
| SGS | Very Good = 1; Good = 2; Fair, Poor = 3 |
| SLASI | Excellent, Very Good = 1; Good = 2; Fair, Poor = 3 |
| Sydney MAS | Excellent, Very Good = 1; Good = 2; Fair, Poor = 3 |

CIRS, Cumulative Illness Rating Scale.
