# Supplementary material for: Determinants of cognitive performance and decline in 20 diverse ethno-regional groups: A COSMIC collaboration cohort study
Source: PLoS Med. 2019 Jul 23;16(7):e1002853. doi: 10.1371/journal.pmed.1002853 (PMC6650056; doi:10.1371/journal.pmed.1002853)
Supplement: S10 Table — (DOCX) [file pmed.1002853.s011.docx]

| **Study** | **Criteria (meeting any is sufficient)** |
| --- | --- |
| CFAS | GMS-AGECAT confidence/severity rating 3+ |
| CHAS | GMS/AGECAT confidence/severity rating 3+ |
| EAS | Beck Anxiety Scale score 11+ |
| ESPRIT | MINI neuropsychiatric exam diagnosis of current anxiety or generalized anxiety disorder |
| HELIAD | Hospital Anxiety & Depression Scale anxiety score 8+ |
| HK-MAPS | 1. NPI part E score 1+  2. CIRS severity rating 1+ |
| Invece.Ab | Use of anxiolytics |
| KLOSCAD | 1. Diagnosis with status as “following-up” or “under treatment”  2. NPI part E (scale score = frequency x severity) |
| PATH | 1. Goldberg Anxiety and Depression Scale anxiety score 5+  2. Anxiety medications taken |
| SGS | Kessler Psychological Distress Scale score 5+ |
| Sydney MAS | 1. Goldberg Anxiety Scale score 5+  2. Use of medication |
| ZARADEMP | GMS-AGECAT diagnosis |

CIRS, Cumulative Illness Rating Scale. GMS-AGECAT, Geriatric Mental State-Automated Geriatric Examination for Computer Assisted Taxonomy, MINI, Mini International Neuropsychiatric Interview, NPI, Neuropsychiatric Inventory.
