# Supplementary material for: Determinants of cognitive performance and decline in 20 diverse ethno-regional groups: A COSMIC collaboration cohort study
Source: PLoS Med. 2019 Jul 23;16(7):e1002853. doi: 10.1371/journal.pmed.1002853 (PMC6650056; doi:10.1371/journal.pmed.1002853)
Supplement: S11 Table — (DOCX) [file pmed.1002853.s012.docx]

| **Study** | **Current (meeting any is sufficient)** | **History** |
| --- | --- | --- |
| Bambui | General Health Questionnaire-12 score 5+ | - |
| CFAS | GMS-AGECAT rating of subcase or clinical case | Diagnosis of depression or manic depression |
| CHAS | DSM-IV major depressive episode, with ICD-10 severity rating of mild (1), moderate (2), or severe (3) | - |
| EAS | GDS score 6+ | - |
| ESPRIT | 1. Current major depressive episode (MINI neuropsychiatric exam)  2. CES-D score 16+ | History of major depressive episode (MINI) |
| HELIAD | Geriatric Depression Scale score 6+ | - |
| HK-MAPS | 1. NPI part D score 1+  2. CIRS severity rating 1+  3. Cornell Scale for Depression in Dementia score 8+ | - |
| Invece.Ab | 1. Use of anti-depressants  2. GDS score 6+  3. Criteria-based diagnosis by physician/ psychologist (including medication, GDS score and CES-D items) | History of depression |
| KLOSCAD | 1. Diagnosis with status as “following-up” or “under treatment”  2. NPI part D  3. Clinical diagnosis of major, minor or sub-syndromal depressive disorder  4. Korean GDS score 16+ | 1. History of depression (including “uncertain” when clarified by status variable), or  2. Clinical diagnosis of major, minor or sub-syndromal depressive disorder |
| LEILA75+ | 1. DSM-IV criteria based on structured clinical interview  2. CES-D score 16+ | Self-reported history |
| PATH | 1. Goldberg Anxiety and Depression Scale depression score 6+  2. Anti-depressants taken | Self-reported history and doctor seen |
| SALSA | 1. CES-D score 16+  2. Anti-depressants taken | - |
| SGS | Kessler Psychological Distress Scale score 5+ | Self-reported history of diagnosis |
| SPAH | ICD-10 rating based on GMS and NPI items covering the past month: mild (1), moderate (2), or severe (3) major depression (vs 0) | - |
| Sydney MAS | 1. GDS score 6+  2. Use of medication | Ever diagnosed |
| Tajiri | GDS 15-item short form score 5+ | - |
| ZARADEMP | GMS-AGECAT rating of subcase or clinical case | - |

CES-D, Centre for Epidemiological Studies depression scale. CIRS, Cumulative Illness Rating Scale. DSM-IV, Diagnostic and Statistical Manual of Mental Disorders (4th edition). GDS, Geriatric Depression Scale. GMS-AGECAT, Geriatric Mental State-Automated Geriatric Examination for Computer Assisted Taxonomy. ICD-10, International Classification of Diseases (10th revision) MINI, Mini International Neuropsychiatric Interview, NPI, Neuropsychiatric Inventory.
