# Supplementary material for: Determinants of cognitive performance and decline in 20 diverse ethno-regional groups: A COSMIC collaboration cohort study
Source: PLoS Med. 2019 Jul 23;16(7):e1002853. doi: 10.1371/journal.pmed.1002853 (PMC6650056; doi:10.1371/journal.pmed.1002853)
Supplement: S12 Table — (DOCX) [file pmed.1002853.s013.docx]

| **Study** | **Criteria (meeting any is sufficient)^a^** |
| --- | --- |
| Bambui | 1. Blood pressure (mean of 2^nd^ and 3^rd^), 2. Medication |
| CFAS | History |
| CHAS | 1. Blood pressure (average), 2. History indicated by diagnosis or treatment |
| EAS | 1. Blood pressure (mean of 2), 2. History |
| ESPRIT | 1. Blood pressure (mean of 2), 2. Medication |
| HELIAD | History |
| HK-MAPS | Cumulative Illness Rating Scale severity rating 1+ |
| Invece.Ab | 1. Medication, 2. Supine blood pressure 170-180 mmHg and history, 3. Supine blood pressure >180 mmHg |
| KLOSCAD | 1. History (also having follow-up current status data or age first diagnosed/began medication), 2. Self-reported current , 3. Blood pressure (mean of 3) |
| LEILA75+ | 1.Blood pressure |
| MAAS | 1. Blood pressure (mean of 5), 2. Medication |
| MoVIES | 1. Blood pressure (right or left: n=338; averaged over both: n=67), 2. History |
| PATH | 1. Blood pressure (mean of 2), 2. Medication |
| SALSA | 1. Blood pressure (mean of 2), 2. Self-reported, 3. Medication |
| SGS | Self-reported history of diagnosis |
| SLASI | 1. Blood pressure (1 reading), 2. Medication, 3. History |
| SPAH | 1. Blood pressure (mean of 3 readings), 2. Medication |
| Sydney MAS | 1. Blood pressure (mean of 2), 2. Medication, 3. History |
| Tajiri | 1. Blood pressure (mean of 2), 2. Medication |
| ZARADEMP | Diagnosis using EURODEM Risk Factor Questionnaire and medical records |

^a^ Blood pressure criteria are seated systolic blood pressure ≥140 mmHg or diastolic blood pressure ≥90 mmHg, unless otherwise indicated.
