# Supplementary material for: Determinants of cognitive performance and decline in 20 diverse ethno-regional groups: A COSMIC collaboration cohort study
Source: PLoS Med. 2019 Jul 23;16(7):e1002853. doi: 10.1371/journal.pmed.1002853 (PMC6650056; doi:10.1371/journal.pmed.1002853)
Supplement: S13 Table — (DOCX) [file pmed.1002853.s014.docx]

| **Study** | **Criteria (meeting any is sufficient)^a^** |
| --- | --- |
| Bambui | 1. Fasting blood glucose, 2. Treatment |
| CFAS | History |
| CHAS | 1. Told had diabetes, 2. Had treatment , 3. Fasting blood glucose |
| EAS | History |
| ESPRIT | 1. Treatment, 2. Fasting blood glucose |
| HELIAD | History |
| HK-MAPS | Cumulative Illness Rating Scale severity rating 1+ |
| Invece.Ab | 1. Treatment, 2. History |
| KLOSCAD | 1. History (also having follow-up current status data or age first diagnosed/began medication), 2. Self-reported current, 3. Fasting blood glucose, 4. Non-fasting blood glucose ≥200mg/dL |
| LEILA75+ | Self-reported |
| MAAS | Self-reported diagnosis or starting medication age 40+ |
| MoVIES | History (includes reported presence >1 month ago at wave 2) |
| PATH | 1. History, 2. Treatment |
| SALSA | 1. Self-report, 2. Fasting blood glucose, 3. Medication |
| SGS | Self-reported history of diagnosis |
| SLASI | 1. Fasting blood glucose, 2. Treatment, 3. History |
| SPAH | 1. Fasting blood glucose, 2. Treatment |
| Sydney MAS | 1. Fasting blood glucose, 2. Treatment, 3. History |
| Tajiri | 1. Fasting blood glucose, 2. Treatment (diet) |
| ZARADEMP | Diagnosis using EURODEM Risk Factor Questionnaire and medical records |

^a^ Fasting blood glucose criteria are ≥126mg/dL or >7mmol/L.
