# Supplementary material for: Determinants of cognitive performance and decline in 20 diverse ethno-regional groups: A COSMIC collaboration cohort study
Source: PLoS Med. 2019 Jul 23;16(7):e1002853. doi: 10.1371/journal.pmed.1002853 (PMC6650056; doi:10.1371/journal.pmed.1002853)
Supplement: S14 Table — (DOCX) [file pmed.1002853.s015.docx]

| **Study** | **Criteria (meeting any is sufficient)^a^** |
| --- | --- |
| Bambui | 1. Cholesterol, 2. Triglycerides |
| CHAS | 1. Cholesterol, 2. Triglycerides |
| EAS | 1. Cholesterol, 2. Triglycerides |
| ESPRIT | 1. Treatment, 2. Cholesterol, 3. Triglycerides |
| HELIAD | History |
| HK-MAPS | Cumulative Illness Rating Scale severity rating 1+ |
| Invece.Ab | 1. Treatment, 2. History |
| KLOSCAD | 1. History (also having follow-up current status data or age first diagnosed/began medication), 2. Self-reported current, 3. Cholesterol, 4. Triglycerides |
| PATH | 1. Treatment |
| SALSA | 1. Medication, 2. Cholesterol, 3. Triglycerides |
| SGS | Self-reported history of diagnosis |
| SLASI | 1. Treatment, 2. History, 3. Triglycerides |
| SPAH | 1. Cholesterol, 2. Triglycerides^b^ |
| Sydney MAS | 1. Treatment, 2. History, 3. Cholesterol, 4. Triglycerides |
| Tajiri | 1. Cholesterol, 2. Triglycerides, 3. Treatment |

^a^ Criteria for cholesterol are ≥240mg/dL or >6.2mmol/L, and triglycerides ≥200mg/dL or >2.3mmol/L.

^b^Original data for 725 participants, with values for another 1227 participants calculated using the Friedewald formula rearranged for measures in mg/dL to TG = 5 x (TC – LDL – HDL).
