# Supplementary material for: Determinants of cognitive performance and decline in 20 diverse ethno-regional groups: A COSMIC collaboration cohort study
Source: PLoS Med. 2019 Jul 23;16(7):e1002853. doi: 10.1371/journal.pmed.1002853 (PMC6650056; doi:10.1371/journal.pmed.1002853)
Supplement: S15 Table — (DOCX) [file pmed.1002853.s016.docx]

| **Study** | **Criteria** |
| --- | --- |
| Bambui | Intermittent claudication based on medical interview |
| CHAS | Intermittent claudication |
| CFAS | Ever diagnosed with intermittent claudication |
| ESPRIT | Currently being followed or treated for lower limb arteritis |
| HELIAD | Self-reported history |
| Invece.Ab | Classified on basis of medical history, medications (anti-aggregants, anti-coagulants, vasodilators) and medical exam |
| KLOSCAD | History of any of atherosclerosis, vascular bypass surgery, other vascular disease (also having follow-up current status data for these) |
| MAAS | Peripheral arterial disease |
| SALSA | Intermittent claudication, deep vein thrombosis, or ankle-arm index <0.90 |
| Sydney MAS | Ever diagnosed with claudication |
