# Supplementary material for: Determinants of cognitive performance and decline in 20 diverse ethno-regional groups: A COSMIC collaboration cohort study
Source: PLoS Med. 2019 Jul 23;16(7):e1002853. doi: 10.1371/journal.pmed.1002853 (PMC6650056; doi:10.1371/journal.pmed.1002853)
Supplement: S16 Table — (DOCX) [file pmed.1002853.s017.docx]

| **Study** | **Criteria** |
| --- | --- |
| Bambui | Diagnosed from ECG |
| Invece.Ab | Medical history, confirmed with ECG if necessary |
| SALSA | Self-reported history of diagnosis |
| SLASI | Self-reported history or medication |
| Sydney MAS | Ever diagnosed |
| Tajiri | Medical history |
