# Supplementary material for: Determinants of cognitive performance and decline in 20 diverse ethno-regional groups: A COSMIC collaboration cohort study
Source: PLoS Med. 2019 Jul 23;16(7):e1002853. doi: 10.1371/journal.pmed.1002853 (PMC6650056; doi:10.1371/journal.pmed.1002853)
Supplement: S17 Table — (DOCX) [file pmed.1002853.s018.docx]

| **Study** | **Criteria (meeting any is sufficient)** |
| --- | --- |
| Bambui | Myocardial infarction or angina |
| CFAS | Angina or heart attack |
| CHAS | Doctor diagnosed any of heart attack, angina, heart failure, valve disease, or other (such as atrial fibrillation or ventricular arrhythmia or cardiomyopathy) |
| EAS | Myocardial infarction, coronary artery bypass, angina, heart failure, angioplasty, or arrhythmia |
| ESPRIT | Ischemic heart disease (defined as any of current angina, history of angioplasty, heart operation or myocardial infarction) or heartbeat disorders (arrhythmia or auricular fibrillation) |
| HELIAD | Coronary disease, myocardial infarction, congestive heart failure, arrhythmia, or any other heart disease |
| HK-MAPS | Cumulative Illness Rating Scale severity rating 1+ for either heart disease (ischemic heart disease or heart failure) or arrhythmia/ atrial fibrillation |
| Invece.Ab | 1. Cardiovascular disease defined by study as any of myocardial infarction, heart failure, angina, arrhythmia, coronary artery bypass graft, or other  2. Medication  3. Atrial fibrillation |
| KLOSCAD | 1. History of any of myocardial infarction, angina, congestive heart failure, arrhythmia, cardiac operation, or other (also having follow-up current status data or age first diagnosed/began medication)  2. Self-reported current cardiac disease |
| LEILA75+ | Self-reported myocardial infarction |
| MAAS | Self-reported myocardial infarction, angina, heart insufficiency, heart valve disease, bypass surgery, or open-heart surgery |
| MoVIES | History of any of myocardial infarction, angina, pacemaker, palpitations, heart murmur, or other (includes reported presence >1 month ago at wave 2) |
| PATH | “Do you have heart trouble?” |
| SALSA | Myocardial infarction, angina, congestive heart failure, atrial fibrillation, or heart/coronary catheterization |
| SGS | Self-reported history of diagnosis |
| SLASI | 1. Heart attack, heart failure, or atrial fibrillation  2. Medication for heart attack, heart failure, or atrial fibrillation |
| Sydney MAS | Heart attack, angina, cardiomyopathy, valve disease, arrhythmia, atrial fibrillation |
| Tajiri | Ischemic heart disease, or atrial fibrillation |
| ZARADEMP | Diagnosis of myocardial infarction or angina using EURODEM Risk Factor Questionnaire and medical records |
