# Supplementary material for: Determinants of cognitive performance and decline in 20 diverse ethno-regional groups: A COSMIC collaboration cohort study
Source: PLoS Med. 2019 Jul 23;16(7):e1002853. doi: 10.1371/journal.pmed.1002853 (PMC6650056; doi:10.1371/journal.pmed.1002853)
Supplement: S18 Table — (DOCX) [file pmed.1002853.s019.docx]

| **Study** | **Criteria** |
| --- | --- |
| Bambui | History of stroke |
| CFAS | History of stroke |
| CHAS | Self-report of a clinical diagnosis |
| EAS | Medical history of stroke |
| ESPRIT | Have you had one or more cerebrovascular attacks (strokes, seizures)? |
| HELIAD | Medical history of stroke or TIA |
| HK-MAPS | Cumulative Illness Rating Scale severity rating 1+ for cerebrovascular disease (CVA, TIA) |
| Invece.Ab | History of stroke or TIA |
| KLOSCAD | History of stroke (sometimes indicated only by having data for a follow-up current status), cerebral infarction, cerebral haemorrhage, TIA, cerebral ischaemia, or “something like stroke”. |
| LEILA75+ | Self-reported history of stroke |
| MoVIES | History of stroke (includes participants assessed at wave 2 indicating presence >1 month ago) |
| PATH | “Have you ever suffered a stroke?” |
| SALSA | Self-report |
| SGS | Self-reported history of diagnosis |
| SLASI | History of stroke or regular medication for stroke |
| SPAH | Diagnosis of stroke or TIA |
| Sydney MAS | Diagnosis of stroke or TIA |
| Tajiri | Medical history |
| ZARADEMP | History of stroke or TIA |

CVA, cerebrovascular accident. TIA, transient ischaemic attack.
