# Supplementary material for: Determinants of cognitive performance and decline in 20 diverse ethno-regional groups: A COSMIC collaboration cohort study
Source: PLoS Med. 2019 Jul 23;16(7):e1002853. doi: 10.1371/journal.pmed.1002853 (PMC6650056; doi:10.1371/journal.pmed.1002853)
Supplement: S19 Table — (DOCX) [file pmed.1002853.s020.docx]

| **Study** | **Nil/minimal=0; At least 1 drink per week = 1; 2+ drinks per week = 2** |
| --- | --- |
| CFAS | “Most drunk per week on a regular basis”: Never had a drink = 0; 1 or more = 1; 2 or more = 2^a^ |
| EAS | Drinks per week calculated from use during past year for each of beer, wine, liquor |
| ESPRIT | Drinks per week calculated from consumption in grams/day using 10 g = 1 drink |
| HELIAD | Drinks per week calculated from frequency and glasses consumed per occasion for each of tsipouro, beer, wine, whisky |
| HK-MAPS | Non-drinker or ex-drinker = 0, current irregular drinker (weekly or less than weekly) = 1, current regular drinker (more than weekly) = 2 |
| KLOSCAD | Number of drinks per week |
| LEILA75+ | Number of drinks per week |
| MoVIES^b^ | Drinks per week calculated using frequency (less than once a month or not in past year = 0; less than weekly but more than once a month = 0.625; once a week = 1; less than daily but more than weekly = 4; daily = 7) and amount typical for a drinking day (in drinks per day) |
| PATH | Drinks per week calculated using frequency (never, not in last year, monthly or less = 0; 2-4 per month = 0.75; 2-3 per week = 2.5; 4+ per week = 6.34^c^) and amount typical for a drinking day (1 or 2 = 1.5; 3 or 4 = 3.5; 5 or 6 = 5.5; 7 to 9 = 8; 10+ = 10). Coded as monthly or less = 0, 2-4 times a month = 1, 2-3 times a week or more = 2 |
| SALSA | Number of drinks per week |
| SGS | Never (0-1 days/week) = 0, Rarely (1-2 days/week) = 1, Sometimes (3-5 days/week) = 2, Almost every day (5+ days/week) = 2 |
| SLASI | Current use: never or <1 drink/week = 0, >1 drink/week = 1, 1-2 drinks/day = 2 |
| Sydney MAS | Drinks per week calculated using frequency (no or monthly or less = 0; 2-4 per month = 0.75; 2-3 per week = 2.5; 4-6 per week = 5; daily = 7) and amount typical for a drinking day (1 = 1; 2 or 3 = 2.5; 4 or 5 = 4.5; 6 or 7 = 6.5; 8+ = 8). Coded as monthly or less = 0, 2-4 times a month = 1, 2-3 times a week or more = 2 |

**^a^** Note: missing data for most participants indicating having ever had a drink mean the proportion of non-drinkers will not be representative of the whole sample.

^b^ Data are from wave 2.

**^c^** Determined from Sydney MAS data showing proportion of 4-6 per week = 33% and daily = 67%.
