# Supplementary material for: Determinants of cognitive performance and decline in 20 diverse ethno-regional groups: A COSMIC collaboration cohort study
Source: PLoS Med. 2019 Jul 23;16(7):e1002853. doi: 10.1371/journal.pmed.1002853 (PMC6650056; doi:10.1371/journal.pmed.1002853)
Supplement: S20 Table — (DOCX) [file pmed.1002853.s021.docx]

| **Study** | **Minimally active = 0; Moderate activity at least once per week = 1; Vigorous activity at least once per week = 2.** |
| --- | --- |
| Bambui | Participation in exercise lasting 20-30 mins during leisure time at least 3 times a week = 2  Note this is the only category in the original data, with no data for other participants. |
| CHAS | “Taking into account both work and leisure, would you say that you are” very physically active (= 2), fairly (= 1), not very (= 0), not at all (= 0) |
| EAS | Responses in terms of days per week  Light activities (walking, gardening, dancing, calisthenics, golf, bowling, horse riding) at least 1 day a week = 1, Medium (hiking, tennis, cycling, swimming) or heavy (jogging, aerobics, hand or racquet ball) activities at least 1 day a week = 2, 0 (or less) days for all categories = 0 |
| ESPRIT | Either of gardening or walking “a little” or “a lot” = 1 vs “very little” = 0, Sports “regularly” or “often” = 2, gardening or walking “very little” and sports “never” or “from time to time” = 0 |
| HELIAD | Response options are “once a year or rarer”, “many times a year (< once a month)”, “many times a month (1 to 5 times a month)”, “may times a week (3 to 4 times a week)”, every day or almost every day (5 to 7 days a week)”  Walking for pleasure or exercise “many times a month (1 to 5 times a month)” or more = 1, Physical exercises (eg, gym, swim) “many times a month (1 to 5 times a month)” or more = 2, both of these variables less frequently = 0 |
| Invece.Ab | Response options are “never”, “1 x week”, “2 x week”, “3+ week”. Any of walking > 30 mins, dancing, “others”, group exercise “1 x week” or more = 1. Any of cycling, swimming, running-jogging, tennis, aerobics “1 x week” or more = 2.  None of these or only walking < 30 mins = 0. |
| KLOSCAD | 1+ day/week performing light exercise (such as stepper in-house, slow social dance, golf on the cart, bowling, walking at a speed of 3 to 5 km/hr, free gymnastics or calisthenics; setting-up exercises; rhythmic gymnastics) = 1, and either moderate (biking at speeds of over 16 km/hr, club-dragging golf, slow swimming, fast walking at a speed of 6 km/hr, doubles tennis, fast ballroom dancing) or vigorous exercise (skating, climbing, running, singles tennis, skiing, intense aerobics) = 2 (regardless of time spent). None of these or only light activities (writing, typing, walking slowly at speeds of less than 3 km/hr) = 0. |
| PATH | Response options are “never/hardly ever”, “about 1-3 times a month”, “once or twice a week”, “3 times a week or more”.  Moderate activity (scrubbing, polishing car, dancing, golf, cycling, decorating, lawn mowing, leisurely swimming) “once or twice a week” or “3 times a week or more” = 1. Vigorous activity (running, hard swimming, tennis, squash, digging, cycle racing) “once or twice a week” “3 times a week or more” = 2. Less frequent participation in these, only mild activity (walking, woodwork, gardening, bike repairs, playing pool, general housework), or less = 0. |
| SALSA | List of activities responded to in hours per week. 1 hour or more of swim or work out = 2; doing yard work, taking walks, doing heavy housework, dancing, hunting or camping or boating, golf or other moderate exercise, walking around your neighbourhood, or climbing at least 5^a^ flights of stairs per day = 1; doing house repairs, baking, doing light housework, cook meals, standing or walking at work or home, sitting at work or home, driving a car |
| SPAH | Taking into account both work and leisure, would you say that you are (1) very physically active; (2) reasonably physically active; (3) not very physically active; (4) not at all physically active. |
| Sydney MAS | Responses given as times (but not always clear what is intended). Any time indicated for separate activities bowls, golf, dancing, walking, other (Pilates, yoga, tai chi, weights) = 1. Any time indicated for tennis, swimming, jogging, bicycling, aerobics, other relevant = 2. Cases with no time indicated or where the time is clearly less than once a week = 0. Note caveat that swimming may often refer to for leisure rather than for exercise (sometimes noted) and the coding as vigorous may include those for whom swimming is really only moderate or less. |

**^a^** In total energy expenditure, 5 flights of stairs per day was calculated as approximately equal to one 30-min walk a week using values from Lee & Paffenbarger (2000): walking 1 block (0.13 km) daily = 235 kJ/week; climbing up and down 1 flight of stairs daily = 118 kJ/week; and assuming a moderate walking pace of 4.8 km/h. Lee IM, Paffenbarger RS, Jr. Associations of light, moderate, and vigorous intensity physical activity with longevity. The Harvard Alumni Health Study. Am J Epidemiol 2000;151:293-9.
