# Supplementary material for: Determinants of cognitive performance and decline in 20 diverse ethno-regional groups: A COSMIC collaboration cohort study
Source: PLoS Med. 2019 Jul 23;16(7):e1002853. doi: 10.1371/journal.pmed.1002853 (PMC6650056; doi:10.1371/journal.pmed.1002853)
Supplement: S21 Table — (DOCX) [file pmed.1002853.s022.docx]

| **Study** | **N^a^** | **Age, yrs** | | **Sex, no. (%)** | | **Education, yrs** | |
| --- | --- | --- | --- | --- | --- | --- | --- |
|  |  | **Range** | **Mean (SD)** | **Female** | **Male** | **Range** | **Mean (SD)** |
| Bambui | 1491 | 60-95 | 68.9 (7.1) | 909 (61.0) | 582 (39.0) | 0-14 | 2.8 (3.0) |
| CFAS | 12256 | 64-105 | 75.0 (6.8) | 7316 (59.7) | 4940 (40.3) | 0-34 | 10.0 (2.3) |
| CHAS | 2574 | 65-100 | 74.3 (6.5) | 1653 (64.2) | 921 (35.8) | 0-18 | 9.3 (4.6) |
| EAS | 2063 | 63-100 | 78.1 (5.3) | 1266 (61.4) | 797 (38.6) | 0-25 | 13.2 (3.6) |
| ESPRIT | 2187 | 65-96 | 73.1 (5.5) | 1277 (58.4) | 910 (41.6) | 0-15 | 10.2 (3.8) |
| HELIAD | 1174 | 54-94 | 73.0 (5.6) | 684 (58.3) | 490 (41.7) | 0-21 | 6.5 (4.1) |
| HK-MAPS | 785 | 60-96 | 72.3 (7.2) | 421 (53.6) | 364 (46.4) | 0-20 | 4.8 (4.7) |
| Invece.Ab | 1267 | 70-75 | 72.2 (1.3) | 684 (54.0) | 583 (46.0) | 0-20 | 6.8 (3.3) |
| KLOSCAD | 6513 | 58-98 | 70.0 (6.7) | 3702 (56.8) | 2811 (43.2) | 0-26 | 8.0 (5.3) |
| LEILA75+ | 1040 | 75-99 | 81.5 (4.8) | 779 (74.9) | 261 (25.1) | 9-16 | 11.9 (1.7) |
| MAAS | 796 | 55-82 | 67.3 (7.3) | 388 (48.7) | 408 (51.3) | 6-17 | 9.4 (2.8) |
| MoVIES | 1613 | 64-105 | 72.7 (5.8) | 935 (58.0) | 678 (42.0) | 3-21 | 11.1 (2.6) |
| PATH | 2545 | 60-66 | 62.5 (1.5) | 1232 (48.4) | 1313 (51.6) | 4-18 | 13.7 (2.8) |
| SALSA | 1710 | 58-98 | 70.3 (6.8) | 999 (58.4) | 711 (41.6) | 0-32 | 7.3 (5.3) |
| SPAH | 1858 | 65-101 | 72.0 (6.1) | 1121 (60.3) | 737 (39.7) | 0-19 | 2.5 (3.0) |
| SGS | 2178 | 65-96 | 73.6 (6.2) | 1252 (57.5) | 926 (42.5) | 0-23 | 11.0 (2.5) |
| SLASI | 793 | 55-88 | 64.6 (6.8) | 484 (61.0) | 309 (39.0) | 0-22 | 7.0 (4.5) |
| Sydney MAS | 1037 | 70-90 | 78.8 (4.8) | 572 (55.2) | 465 (44.8) | 3-24 | 11.6 (3.5) |
| Tajiri | 100 | 65-80 | 71.2 (4.0) | 58 (58.0) | 42 (42.0) | 2-13 | 8.0 (1.8) |
| ZARADEMP | 4542 | 58-102 | 73.4 (9.5) | 2586 (56.9) | 1956 (43.1) | 1-18 | 7.1 (3.9) |

^a^ Size of the analyzed sample, with individuals excluded from the original cohort if classified with dementia at baseline, or missing age, sex, education or dementia status data; individuals from MAAS aged less than 55 years were also not included.
