# Supplementary material for: Determinants of cognitive performance and decline in 20 diverse ethno-regional groups: A COSMIC collaboration cohort study
Source: PLoS Med. 2019 Jul 23;16(7):e1002853. doi: 10.1371/journal.pmed.1002853 (PMC6650056; doi:10.1371/journal.pmed.1002853)
Supplement: S22 Table — (DOCX) [file pmed.1002853.s023.docx]

| **Study** | **Body mass index (kg/m^2^)** | **Systolic blood pressure (mm Hg)** | **Diastolic blood pressure (mm Hg)** | **Pulse pressure (mm Hg)** |
| --- | --- | --- | --- | --- |
| Bambui | 25.1 (4.8); 1381 | 137.2 (22.0); 1405 | 83.4 (12.4); 1405 | 53.7 (15.8); 1405 |
| CFAS | - | - | - | - |
| CHAS | 24.8 (5.1); 1150 | 146.4 (25.4); 2570 | 82.1 (12.9); 2570 | 64.2 (19.0); 2570 |
| EAS | 28.3 (5.3); 716 | 134.1 (15.9); 716 | 77.2 (8.5); 716 | 56.8 (15.7); 716 |
| ESPRIT | 25.1 (3.6); 2171 | 140.9 (17.3); 2182 | 79.6 (9.8 2182 | 61.2 (13.5); 2182 |
| HELIAD | 29.3 (4.4); 1148 | - | - | - |
| HK-MAPS | - | - | - | - |
| Invece.Ab | 27.2 (4.6); 1201 | - | - | - |
| KLOSCAD | 24.0 (3.0); 5839 | 126.1 (14.4); 5948 | 77.8 (9.0); 5948 | 48.2 (10.5); 5948 |
| LEILA75+ | - | 158.6 (24.1); 967 | 85.9 (14.8); 967 | 72.2 (20.8); 967 |
| MAAS | 27.7 (3.9); 794 | 144.2 (20.2); 781 | 76.9 (11.9); 781 | 67.3 (15.4); 781 |
| MoVIES | 26.2 (5.0); 266 | 141.7 (19.1); 373 | 78.5 (9.9); 373 | 63.1 (14.5); 373 |
| PATH | 26.7 (4.6); 2316 | 139.8 (19.4); 2483 | 83.0 (10.6); 2483 | 56.7 (14.1); 2483 |
| SALSA | 29.7 (5.4); 1565 | 138.3 (18.9); 1579 | 75.9 (10.4); 1577 | 62.4 (17.3); 1577 |
| SGS | 23.2 (3.1); 1917 | - | - | - |
| SLASI | 23.7 (3.6); 793 | 132.5 (16.4); 793 | 81.4 (9.1); 793 | 51.2 (12.8); 793 |
| SPAH | 25.8 (4.7); 1774 | 145.6 (25.6); 1775 | 86.0 (13.4); 1775 | 59.6 (18.2); 1775 |
| Sydney MAS | 27.1 (4.4); 1010 | 144.6 (20.7); 1015 | 81.8 (10.8); 1015 | 62.8 (15.3); 1015 |
| Tajiri | - | 141.3 (19.5); 100 | 82.3 (12.0); 100 | 59.0 (14.4); 100 |
| ZARADEMP | 26.9 (4.9); 4536 | - | - | - |
|  |  |  |  |  |
| Total | 25.9 (4.6); 29370 | 137.8 (21.3); 23480 | 80.6 (11.3); 23478 | 57.2 (16.5); 23478 |

Note: Values are presented as mean (SD). Outlying values were Winsorized to ±3 SD within each study.
