# Supplementary material for: Determinants of cognitive performance and decline in 20 diverse ethno-regional groups: A COSMIC collaboration cohort study
Source: PLoS Med. 2019 Jul 23;16(7):e1002853. doi: 10.1371/journal.pmed.1002853 (PMC6650056; doi:10.1371/journal.pmed.1002853)
Supplement: S23 Table — (DOCX) [file pmed.1002853.s024.docx]

| **Study** | ***APOE*4*** | **Health: very good, good, poor** | **Anxiety** | **Depression** | **History of Depression** | **Hypertension** | **Diabetes** | **High cholesterol** |
| --- | --- | --- | --- | --- | --- | --- | --- | --- |
| Bambui | 25.1 (1326) | 35.4, 45.3, 19.2 (1491) | - | 37.8 (1474) | - | 69.2 (1444) | 15.1 (1393) | 50.0 (1407) |
| CFAS | 23.8 (981) | 19.9, 48.4, 31.7 (12194) | 3.3 (2016) | 34.6 (2016) | 9.0 (12182) | 32.8 (12205) | 5.9 (12213) | - |
| CHAS | 16.1 (2199) | - | 4.2 (2574) | 1.2 (2574) | - | 74.6 (2570) | 31.9 (2103) | 30.0 (2015) |
| EAS | 22.4 (917) | 31.6, 42.1, 26.3 (1673) | 9.7 (370) | 11.7 (1701) | - | 63.4 (2018) | 18.1 (2027) | 15.7 (623) |
| ESPRIT | 19.3 (2142) | - | 13.5 (1974) | 30.7 (2176) | 26.2 (1962) | 71.8 (2187) | 9.3 (2187) | 56.5 (2187) |
| HELIAD | 16.2 (969) | - | 15.1 (1173) | 26.9 (1172) | - | 69.0 (1172) | 18.6 (1173) | 41.9 (1173) |
| HK-MAPS | 14.1 (263) | 33.2, 47.5, 19.3 (758) | 11.7 (780) | 27.6 (783) | - | 50.0 (758) | 18.5 (758) | 14.0 (758) |
| Invece.Ab | 17.9 (1265) | 24.3, 55.9, 19.8 (1253) | 17.5 (1267) | 16.1 (1267) | 22.7 (1218) | 60.6 (1267) | 17.6 (1267) | 33.2 (1267) |
| KLOSCAD | 24.6 (3709) | 37.4, 47.2, 15.4 (6188) | 1.8 (6308) | 23.8 (6439) | 10.9 (6300) | 60.6 (6422) | 28.7 (5413) | 47.5 (5373) |
| LEILA75+ | 15.9 (245) | 30.2, 50.4, 19.4 (1018) | - | 33.9 (1040) | 8.8 (1031) | 80.6 (967) | 22.4 (1039) | - |
| MAAS | 25.0 (440) | - | - | - | - | 65.6 (796) | 7.9 (796) | - |
| MoVIES | 20.8 (896) | 24.5, 53.0, 22.4 (1301) | - | - | - | 70.7 (376) | 15.4 (584) | - |
| PATH | 27.0 (2374) | 58.7, 28.8, 12.5 (2543) | 20.6 (2544) | 9.6 (2544) | 26.8 (2535) | 66.2 (2543) | 7.6 (2543) | 22.9 (2544) |
| SALSA | 13.8 (1550) | 19.6, 70.4, 10.0 (1685) | - | 29.8 (1690) | - | 67.5 (1710) | 32.5 (1710) | 51.3 (1567) |
| SGS | - | 8.6, 70.4, 21.0 (2163) | 29.6 (2080) | 29.6 (2080) | 0.8 (2178) | 38.5 (2178) | 13.1 (2178) | 10.2 (2178) |
| SLASI | 16.0 (789) | 20.1, 49.2, 30.7 (792) | - | - | - | 61.3 (793) | 14.5 (793) | 47.2 (793) |
| SPAH | - | - | - | 4.7 (1844) | - | 80.5 (1818) | 22.6 (1760) | 32.0 (1746) |
| Sydney MAS | 22.6 (964) | 41.3, 42.2, 16.5 (1034) | 7.2 (1037) | 15.0 (1037) | 16.3 (1002) | 82.9 (1037) | 15.6 (1037) | 68.3 (1037) |
| Tajiri | - | - | - | 26.0 (100) | - | 72.0 (100) | 10.0 (100) | 16.0 (100) |
| ZARADEMP | - | - | 4.6 (4542) | 19.5 (4123) | - | 68.2 (4541) | 12.6 (4501) | - |
|  |  |  |  |  |  |  |  |  |
| Total | 20.8 (21818) | 27.9, 49.1, 23.0 (34885) | 9.4 (26665) | 21.2 (34060) | 12.4 (28408) | 57.3 (47695) | 15.3 (46368) | 38.5 (25561) |

Note: Data are presented as % (valid denominator).

# (continued)

| **Study** | **Peripheral vascular disease** | **Atrial fibrillation** | **Cardiovascular disease** | **Stroke** | **Smoking: never, past, current** | **Alcohol use: nil, 1 drink/week, 2+ drinks/week** | **Physical activity: minimal, moderate, vigorous** |
| --- | --- | --- | --- | --- | --- | --- | --- |
| Bambui | 2.4 (1399) | 3.2 (1393) | 15.5 (1408) | 3.4 (1405) | - | - | - |
| CFAS | 2.1 (12195) | - | 18.0 (12233) | 6.7 (12217) | 34.2, 47.0, 18.8 (12181) | 63.4, 2.5, 34.1 (2890) | - |
| CHAS | 4.5 (201) | - | 30.7 (2568) | 6.4 (2573) | 53.8, 26.6, 19.6 (2573) | - | 27.3, 45.6, 27.1 (2568) |
| EAS | - | - | 34.5 (2036) | 9.6 (1977) | 45.2, 47.8, 7.1 (2035) | 52.2, 10.3, 37.4 (1459) | 39.6, 35.5, 24.8 (813) |
| ESPRIT | 3.6 (2159) | - | 20.4 (2187) | 3.3 (2165) | 57.9, 35.5, 6.7 (2186) | 24.5, 5.6, 69.9 (2146) | 16.2, 46.5, 37.4 (1943) |
| HELIAD | 2.7 (1172) | - | 24.5 (1172) | 8.4 (1173) | 64.6, 26.3, 9.1 (1172) | 58.8, 7.1, 34.1 (1162) | 51.5, 41.9, 6.6 (1173) |
| HK-MAPS | - | - | 16.6 (758) | 7.5 (758) | 84.0^a^, 16.0 (781) | 94.1, 5.9, 0.0 (781) | - |
| Invece.Ab | 3.7 (1267) | 2.1 (1267) | 27.1 (1267) | 8.1 (1264) | 57.5, 32.9, 9.6 (1267) | - | 32.5, 30.6, 37.0 (1266) |
| KLOSCAD | 1.2 (6297) | - | 12.6 (6270) | 9.2 (6418) | 69.9, 18.5, 11.5 (6341) | 72.8, 3.8, 23.4 (6357) | 34.5, 36.4, 29.1 (6358) |
| LEILA75+ | - | - | 8.5 (1037) | 6.2 (1040) | 67.7, 25.7, 6.6 (1031) | 13.1, 0.0, 86.9 (1030) | - |
| MAAS | 4.6 (796) | - | 17.5 (796) | - | 35.3, 43.0, 21.7 (793) | - | - |
| MoVIES | - | - | 41.8 (584) | 9.5 (590) | 52.9, 36.6, 10.4 (1302) | 62.3, 7.4, 30.3 (700) | - |
| PATH | - | - | 15.2 (2539) | 4.5 (2543) | 51.9, 37.3, 10.8 (2543) | 30.4, 15.0, 54.6 (2542) | 32.7, 47.8, 19.5 (2533) |
| SALSA | 26.2 (1533) | 6.2 (1685) | 22.4 (1691) | 8.5 (1710) | 45.9, 42.7, 11.4 (1709) | 77.4, 4.3, 18.3 (1706) | 12.2, 64.6, 23.9 (1710) |
| SGS | - | - | 13.4 (2178) | 3.9 (2178) | 65.4, 26.2, 8.4 (2163) | 32.1, 27.0, 40.8 (2169) | - |
| SLASI | - | 2.5 (791) | 10.0 (793) | 2.5 (793) | 84.5, 9.1, 6.4 (792) | 94.8, 3.0, 2.1 (792) | - |
| SPAH | - | - | - | 10.8 (1857) | 75.3^a^, 24.7 (1026) | - | 21.3, 51.0, 27.7 (1853) |
| Sydney MAS | 21.8 (1021) | 6.7 (1023) | 30.1 (1037) | 4.0 (1026) | 46.0, 50.8, 3.2 (1035) | 38.7, 5.7, 55.6 (1037) | 18.9, 48.8, 32.3 (1037) |
| Tajiri | - | 1.0 (100)^b^ | 2.0 (100)^b^ | 0.0 (100)^b^ | 71.1, 11.3, 17.5 (97) | - | - |
| ZARADEMP | - | - | 7.0 (4533) | 5.8 (4503) | 65.3, 21.4, 13.3 (4536) | - | - |
|  |  |  |  |  |  |  |  |
| Total | 4.2 (28040) | 4.3 (6159) | 18.0 (45087) | 6.8 (46190) | 53.6, 32.8, 13.5 (45563) | 55.2, 7.6, 37.2 (24771) | 29.0, 43.8, 27.1 (21254) |

Note: Data are presented as % (valid denominator).

^a^ Value for “not current smoker” and does not distinguish between past and never.

^b^ Models containing these data did not converge and the numbers do not contribute to the totals.
