# Supplementary material for: Determinants of cognitive performance and decline in 20 diverse ethno-regional groups: A COSMIC collaboration cohort study
Source: PLoS Med. 2019 Jul 23;16(7):e1002853. doi: 10.1371/journal.pmed.1002853 (PMC6650056; doi:10.1371/journal.pmed.1002853)
Supplement: S24 Table — (DOCX) [file pmed.1002853.s025.docx]

| **Factor** | **Performance** | | | | **Decline^a^** | | | |
| --- | --- | --- | --- | --- | --- | --- | --- | --- |
|  | **Global cognition** | | **MMSE** | | **Global cognition** | | **MMSE** | |
|  | **B (SE)** | **I^2^ (%)** | **B (SE)** | **I^2^ (%)** | **B (SE)** | **I^2^ (%)** | **B (SE)** | **I^2^ (%)** |
| Age at baseline^b^ | -0.093 (0.01)*** | 92.1 | -0.052 (0.004)*** | 91.9 | -0.068 (0.005)*** | 36.8 | -0.048 (0.006)*** | 85.4 |
| Education^b^ | 0.129 (0.012)*** | 84.9 | 0.098 (0.01)*** | 92.7 | -0.002 (0.01) | 37.8 | -0.014 (0.009) | 64.2 |
| Education-squared^b^ | -0.002 (0.001) | 72.7 | 0 (0.005) | 99.6 | 0.001 (0.001) | 6.5 | 0 (0.001) | 56.9 |
| Sex, male^c^ | -0.024 (0.064) | 86.0 | 0.029 (0.044) | 92.1 | -0.06 (0.035) | 0.8 | 0.002 (0.04) | 61.5 |
| Alcohol *1 drink/week*^c^ | 0.118 (0.083) | 0 | 0.183 (0.073)* | 11.3 | 0.1 (0.099) | 0.2 | 0.082 (0.077) | 0 |
| *2+ drinks/week*^c^ | 0.072 (0.047) | 0 | 0.126 (0.055)* | 46.6 | 0.106 (0.073) | 19.9 | 0.122 (0.046)** | 0 |
| Anxiety | -0.15 (0.073)* | 0 | -0.105 (0.055) | 0 | 0.28 (0.173) | 30.4 | 0.112 (0.068) | 0 |
| *APOE*4* carrier | -0.197 (0.062)** | 18.9 | -0.037 (0.034) | 1.9 | -0.282 (0.081)*** | 24.6 | -0.085 (0.045) | 8.6 |
| Atrial fibrillation | -1.862 (1.229) | 74.0 | 0.042 (0.161) | 32.9 | -22.16 (16.722) | 73.5 | -0.07 (0.157) | 0 |
| Blood pressure, diastolic | 0.005 (0.003) | 9.3 | 0 (0.001) | 0 | 0.004 (0.003) | 0 | 0.003 (0.002) | 9.5 |
| Blood pressure, systolic | 0.001 (0.002) | 33.3 | -0.001 (0.001) | 0 | 0.001 (0.002) | 0 | 0.001 (0.001) | 0 |
| Body mass index^d^ | -0.002 (0.001)* | 0 | -0.001 (<0.001) | 0 | 0 (0.001) | 27.6 | <0.001 (<0.001) | 0 |
| Body mass index-squared | -0.003 (0.005) | 1.6 | 0.003 (0.004) | 21.9 | -0.004 (0.008) | 3.7 | 0.001 (0.007) | 51.2 |
| Cardiovascular disease | -0.05 (0.071) | 49.1 | 0.03 (0.03) | 18.6 | 0.084 (0.057) | 0 | 0.104 (0.038)** | 1.7 |
| Depression, current | -0.275 (0.099)** | 67.1 | -0.146 (0.037)*** | 22.6 | -0.099 (0.069) | 0.1 | -0.064 (0.057) | 25.1 |
| Depression, history | -0.035 (0.062) | 0 | 0.008 (0.067) | 33.1 | 0.053 (0.08) | 0 | -0.02 (0.052) | 0.2 |
| Diabetes | -0.294 (0.052)*** | 0 | -0.152 (0.028)*** | 0 | -0.127 (0.111) | 32.0 | -0.083 (0.067) | 36.4 |
| Health *Good*^e^ | -0.155 (0.103) | 61.4 | 0.019 (0.049) | 45.0 | -0.084 (0.107) | 42.0 | 0.07 (0.045) | 20.7 |
| *Poor*^e^ | -0.558 (0.097)*** | 40.3 | -0.255 (0.065)*** | 47.5 | -0.238 (0.11)* | 19.4 | -0.038 (0.064) | 26.3 |
| High cholesterol | -0.031 (0.044) | 0.01 | -0.013 (0.031) | 0 | 0.042 (0.062) | 0 | -0.054 (0.04) | 0 |
| Hypertension | -0.109 (0.05)* | 22.4 | -0.02 (0.019) | 0 | -0.103 (0.07) | 17.0 | 0.037 (0.031) | 0.1 |
| Peripheral vascular disease | -0.298 (0.131)* | 10.7 | -0.044 (0.052) | 0 | -0.217 (0.27) | 42.6 | -0.092 (0.086) | 0 |
| Physical activity *Moderate*^f^ | 0.093 (0.079) | 33.8 | 0.115 (0.063) | 37.1 | 0.008 (0.088) | 0 | 0.045 (0.098) | 33.5 |
| *Vigorous*^f^ | 0.211 (0.066)** | 0 | 0.205 (0.051)*** | 0 | -0.062 (0.179) | 45.2 | 0.06 (0.112) | 37.6 |
| Pulse pressure | -0.001 (0.002) | 8.4 | -0.002 (0.001) | 0 | 0 (0.002) | 0 | 0 (0.001) | 0 |
| Smoking *Past*^g^ | 0.108 (0.085) | 64.5 | 0.011 (0.024) | 5.6 | -0.1 (0.05)* | 0 | -0.005 (0.035) | 0 |
| *Current*^g^ | -0.18 (0.125) | 57.3 | -0.119 (0.056)* | 45.6 | -0.084 (0.104) | 9.7 | -0.079 (0.089) | 44.7 |
| Stroke history | -0.344 (0.113)** | 37.2 | -0.337 (0.058)*** | 31.4 | -0.377 (0.181)* | 35.7 | -0.234 (0.096)* | 30.7 |
| Baseline score | NA |  | NA |  | 0.035 (0.03) | 80.6 | -0.044 (0.022)* | 11.2 |

Note: Results are B (SE) at the mean time in study (3.1 y) and controlled for age at baseline (mean = 73.1 y), education (mean = 9.0 y) and sex (40% female); -B indicates worse performance or more decline. Global cognition was calculated as a composite of four neuropsychological tests, each representing one of four cognitive domains: memory, language, processing speed, and executive functioning. MMSE denotes Mini-Mental State Examination. *P < .05, **P < .01, ***P < .001.

^a^ Factor x time interactions.

^b^ Partially adjusted model values for age, education and sex are from the simple model with no other factors included.

^c^ vs. nil/minimal alcohol.

^d^ Centred at mean = 25.2 kg/m2.

^e^ vs. very good^.^

^f^ 1+ times/week vs. minimal activity.

^g^ vs. never smoked.
