# Supplementary material for: Determinants of cognitive performance and decline in 20 diverse ethno-regional groups: A COSMIC collaboration cohort study
Source: PLoS Med. 2019 Jul 23;16(7):e1002853. doi: 10.1371/journal.pmed.1002853 (PMC6650056; doi:10.1371/journal.pmed.1002853)
Supplement: S25 Table — (DOCX) [file pmed.1002853.s026.docx]

|  | **Global cognition** | | | | **MMSE** | | | |
| --- | --- | --- | --- | --- | --- | --- | --- | --- |
|  | **TIS** | | **TIS^2^** | | **TIS** | | **TIS^2^** | |
|  | **B (SE)** | **I^2^ (%)** | **B (SE)** | **I^2^ (%)** | **B (SE)** | **I^2^ (%)** | **B (SE)** | **I^2^ (%)** |
| No Risk Factors | -0.859 (0.253)*** | 97.4 | -0.049 (0.023)* | 89.7 | -0.452 (0.168)** | 98.2 | -0.072 (0.042) | 99.3 |
| Alcohol, 3 categories^a^ | -0.648 (0.193)*** | 90.2 | -0.047 (0.028) | 84.1 | -0.505 (0.248)* | 96.9 | -0.059 (0.042) | 96.2 |
| Alcohol, any | -0.687 (0.201)*** | 90.0 | -0.049 (0.028) | 82.6 | -0.541 (0.197)** | 96.7 | -0.079 (0.038)* | 97.7 |
| Anxiety | -0.745 (0.241)** | 91.8 | -0.04 (0.031) | 66.1 | -0.39 (0.203) | 95.3 | -0.106 (0.066) | 97.5 |
| *APOE*4* | -0.584 (0.216)** | 94.9 | -0.055 (0.022)* | 83.9 | -0.436 (0.23) | 98.2 | -0.054 (0.041) | 98.8 |
| Atrial fibrillation | -3.204 (1.627)* | 80.9 | -0.659 (0.412) | 80.4 | -0.225 (0.369) | 97.5 | -0.155 (0.054)** | 94.6 |
| Body mass index | -1.21 (0.335)*** | 96.4 | -0.083 (0.032)** | 84.5 | -0.464 (0.251) | 98.3 | -0.086 (0.052) | 98.9 |
| Body mass index, 6 categories^b^ | -0.733 (0.377) | 87.8 | -0.09 (0.052) | 62.6 | -0.428 (0.188)* | 81.7 | -0.057 (0.047) | 83.8 |
| Cholesterol, high | -0.929 (0.269)*** | 96.4 | -0.05 (0.026)* | 85.4 | -0.473 (0.204)* | 98.5 | -0.071 (0.04) | 99.0 |
| Cardiovascular disease | -0.569 (0.197)** | 80.9 | -0.071 (0.04) | 68.3 | -0.288 (0.184) | 92.4 | -0.06 (0.052) | 96.6 |
| Diastolic blood pressure | -0.804 (0.335)* | 96.8 | -0.047 (0.031) | 80.8 | -0.334 (0.136)* | 93.5 | -0.09 (0.042)* | 98.2 |
| Depression | -0.809 (0.347)* | 92.8 | -0.045 (0.046) | 72.0 | -0.2 (0.191) | 93.8 | -0.071 (0.055) | 97.8 |
| Depression, history | -0.831 (0.25)*** | 96.1 | -0.046 (0.026) | 87.6 | -0.441 (0.164)** | 97.7 | -0.076 (0.037)* | 98.9 |
| Diabetes | -0.801 (0.246)** | 94.4 | -0.072 (0.03)* | 87.2 | -0.256 (0.17) | 96.4 | -0.041 (0.043) | 98.9 |
| Education | -0.928 (0.311)** | 97.7 | -0.036 (0.036) | 92.4 | -0.45 (0.145)** | 96.6 | -0.08 (0.041) | 98.6 |
| Health, 3 categories^c^ | -0.544 (0.19)** | 79.1 | -0.091 (0.02)*** | 55.4 | -0.496 (0.162)** | 91.6 | -0.083 (0.039)* | 97.3 |
| Hypertension | -0.482 (0.183)** | 82.4 | -0.024 (0.036) | 82.2 | -0.447 (0.127)*** | 91.5 | -0.077 (0.035)* | 97.1 |
| Physical Activity, any | -0.542 (0.303) | 79.2 | -0.062 (0.052) | 53.2 | -0.213 (0.245) | 83.2 | -0.094 (0.087) | 92.2 |
| Physical Activity, 3 categories^d^ | -0.548 (0.305) | 81.7 | -0.062 (0.051) | 55.4 | -0.297 (0.217) | 78.5 | -0.114 (0.098) | 93.9 |
| Pulse pressure | -0.81 (0.251)** | 94.7 | -0.074 (0.03)* | 87.4 | -0.244 (0.167) | 96.3 | -0.039 (0.043) | 98.9 |
| Peripheral vascular disease | -1.311 (0.646)* | 98.5 | -0.054 (0.042) | 66.2 | -0.639 (0.324)* | 99.2 | -0.143 (0.071)* | 99.5 |
| Systolic blood pressure | -0.706 (0.217)** | 94.3 | -0.044 (0.021)* | 77.8 | -0.388 (0.205) | 97.3 | -0.082 (0.043) | 97.9 |
| Smoking^e^ | -0.699 (0.215)** | 94.2 | -0.044 (0.021)* | 77.7 | -0.403 (0.187)* | 96.8 | -0.082 (0.043) | 97.9 |
| Smoke, ever | -0.88 (0.267)*** | 96.9 | -0.045 (0.023) | 85.3 | -0.478 (0.205)* | 98.5 | -0.077 (0.046) | 99.3 |
| Stroke | -0.812 (0.25)** | 94.6 | -0.073 (0.03)* | 86.7 | -0.248 (0.17) | 96.5 | -0.04 (0.043) | 98.9 |
| Fully adjusted model 2 | -0.339 (0.352) | 60.2 | -0.023 (0.059) | 16.3 | -0.383 (0.275) | 69.3 | -0.078 (0.086) | 80.5 |
| Fully adjusted model 1 (APOE) | -0.342 (0.189) | 48.1 | -0.047 (0.04) | 14.9 | -0.184 (0.21) | 77.3 | -0.094 (0.058) | 86.2 |
| Fully adjusted model 1 (BMI) | -0.248 (0.26) | 82.9 | 0.01 (0.041) | 70.0 | -0.23 (0.227) | 76.5 | -0.112 (0.06) | 86.3 |
| Fully adjusted model 1 (Depression) | -0.443 (0.264) | 87.4 | -0.014 (0.034) | 60.9 | -0.355 (0.215) | 79.2 | -0.1 (0.055) | 85.5 |

*P < .05, **P < .01, ***P < .001.

^a^ Included comparisons of both 1 drink/week and 2+ drinks/week vs nil/minimal alcohol use.

^b^ Included comparisons of 5 categories (Underweight, <18.5 kg/m^2^; Upper Normal, 23 to <25; Overweight, 25 to <30; Obese 1A, 30 to <32.5; Obese 1B, 32.5+) vs Lower Normal (18.5 to < 23).

^c^ Included comparisons of both poor and good vs very good.

^d^ Included comparisons of both moderate and vigourous vs nil/minimal.

^e^ Included comparisons of both past and present vs never.
