# Supplementary material for: Determinants of cognitive performance and decline in 20 diverse ethno-regional groups: A COSMIC collaboration cohort study
Source: PLoS Med. 2019 Jul 23;16(7):e1002853. doi: 10.1371/journal.pmed.1002853 (PMC6650056; doi:10.1371/journal.pmed.1002853)
Supplement: S26 Table — (DOCX) [file pmed.1002853.s027.docx]

|  | **Global cognition** | | **MMSE** | |
| --- | --- | --- | --- | --- |
|  | **B (SE)** | **I^2^ (%)** | **B (SE)** | **I^2^ (%)** |
| Alcohol, 1 drink/week | 0.029 (0.048) | 61.4 | 0.012 (0.022) | 13.7 |
| Alcohol, 2+ drinks/week | -0.013 (0.011) | 0 | -0.01 (0.011) | 10.0 |
| Alcohol, any | -0.012 (0.012) | 6.1 | -0.011 (0.009) | 12.3 |
| Anxiety | -0.058 (0.065) | 29.3 | -0.017 (0.023) | 0 |
| *APOE*4* | -0.012 (0.01) | 0 | -0.023 (0.007)** | 0 |
| Atrial fibrillation | -5.124 (4.081) | 71.8 | -0.005 (0.052) | 24.5 |
| Body mass index | 0.001 (0.001)** | 0 | <0.001 (0.001) | 0 |
| Cholesterol, high | 0.051 (0.022)* | 0 | -0.002 (0.007) | 0 |
| Cardiovascular disease | 0.016 (0.025) | 48.2 | -0.017 (0.013) | 42.1 |
| Diastolic blood pressure | -0.001 (0.001) | 9.6 | <0.001 (<0.001) | 0 |
| Depression | 0.006 (0.024) | 2.3 | 0.002 (0.009) | 4.3 |
| Depression, history | 0.003 (0.061) | 36.4 | 0.002 (0.029) | 54.7 |
| Diabetes | 0.034 (0.016)* | 1.5 | 0.007 (0.012) | 16.4 |
| Education | -0.003 (0.003) | 47.4 | 0.001 (0.001) | 31.0 |
| Health, good | 0.033 (0.015)* | 16.2 | -0.019 (0.006)** | 0 |
| Health, poor | 0.049 (0.013)*** | 0 | -0.004 (0.008) | 0.3 |
| Hypertension | -0.007 (0.018) | 24.9 | -0.009 (0.011) | 44.6 |
| Physical activity, any | -0.02 (0.028) | 0 | -0.003 (0.029) | 26.2 |
| Physical activity, moderate | -0.025 (0.03) | 0 | -0.002 (0.029) | 21.0 |
| Physical activity, vigorous | -0.018 (0.031) | 0 | 0 (0.041) | 41.7 |
| Pulse pressure | <0.001 (0.001) | 0 | <0.001 (<0.001) | 4.4 |
| Peripheral vascular disease | 0.107 (0.048)* | 0 | 0.009 (0.021) | 3.9 |
| Systolic blood pressure | <0.001 (<0.001) | 0 | <0.001 (<0.001) | 0 |
| Smoke, ever | -0.004 (0.009) | 4.5 | -0.001 (0.008) | 6.8 |
| Smoking, current | 0.022 (0.022) | 14.0 | 0.035 (0.027) | 66.3 |
| Smoking, past | -0.01 (0.012) | 14.7 | -0.004 (0.008) | 2.7 |
| Stroke | -0.028 (0.027) | 6.6 | 0.004 (0.016) | 5.8 |

*P < .05, **P < .01, ***P < .001.
