# Supplementary material for: Determinants of cognitive performance and decline in 20 diverse ethno-regional groups: A COSMIC collaboration cohort study
Source: PLoS Med. 2019 Jul 23;16(7):e1002853. doi: 10.1371/journal.pmed.1002853 (PMC6650056; doi:10.1371/journal.pmed.1002853)
Supplement: S27 Table — (DOCX) [file pmed.1002853.s028.docx]

|  | **With BMI** | | | | **With current depresion** | | | |
| --- | --- | --- | --- | --- | --- | --- | --- | --- |
|  | **Global cognition** | | **MMSE** | | **Global cognition** | | **MMSE** | |
|  | **B (SE)** | **I^2^ (%)** | **B (SE)** | **I^2^ (%)** | **B (SE)** | **I^2^ (%)** | **B (SE)** | **I^2^ (%)** |
| Age | -0.06 (0.012)*** | 79.8 | -0.049 (0.006)*** | 52.4 | -0.057 (0.011)*** | 80.2 | -0.048 (0.006)*** | 36.1 |
| Alcohol 1 drink/week | 0.076 (0.078) | 0 | 0.12 (0.103) | 14.0 | 0.077 (0.076) | 0 | 0.186 (0.081)* | 0 |
| Alcohol 1+ drinks/week | 0.104 (0.056) | 21.4 | 0.077 (0.055) | 33.3 | 0.112 (0.054)* | 18.4 | 0.086 (0.056) | 19.5 |
| Alcohol 2+ drinks/week | 0.121 (0.077) | 45.9 | 0.045 (0.083) | 40.6 | 0.135 (0.074) | 43.8 | 0.048 (0.058) | 19.8 |
| Body mass index | 0.001 (0.007) | 35.8 | -0.001 (0.01) | 35.9 |  |  |  |  |
| Cholesterol, high | 0.042 (0.057) | 33.9 | -0.059 (0.055) | 32.4 | 0.006 (0.042) | 13.5 | 0.013 (0.061) | 38.7 |
| Cardiovascular disease | 0 (0.041) | 0 | 0.157 (0.098) | 17.4 | -0.055 (0.058) | 24.3 | 0.08 (0.061) | 25.7 |
| Depression |  |  |  |  | -0.039 (0.082) | 59.1 | -0.065 (0.064) | 34.7 |
| Diabetes | -0.022 (0.064) | 20.0 | -0.079 (0.12) | 39.1 | -0.048 (0.045) | 0 | -0.11 (0.081) | 44.8 |
| Education | 0.048 (0.008)*** | 49.2 | -0.003 (0.007) | 65.2 | 0.049 (0.006)*** | 35.0 | 0.095 (0.01)*** | 73.6 |
| Hypertension | -0.033 (0.038) | 0 | 0.05 (0.052) | 4.0 | -0.02 (0.036) | 0 | 0.008 (0.049) | 16.3 |
| Sex (male) | -0.437 (0.07)*** | 63.3 | 0.018 (0.076) | 72.6 | -0.415 (0.068)*** | 67.9 | -0.063 (0.086) | 78.0 |
| Smoke, ever | 0.003 (0.054) | 25.7 | -0.054 (0.051) | 0 | 0.011 (0.068) | 47.1 | -0.04 (0.047) | 12.6 |
| Smoking, current | -0.19 (0.075)* | 0 | -0.131 (0.181) | 0 | -0.182 (0.075)* | 0 | -0.218 (0.08)** | 0 |
| Smoking, past | 0.054 (0.07) | 44.6 | -0.064 (0.055) | 0 | 0.065 (0.074) | 49.3 | -0.012 (0.042) | 0 |
| Stroke | -0.053 (0.121) | 30.9 | -0.159 (0.232) | 0 | -0.077 (0.099) | 19.8 | -0.225 (0.088)* | 9.5 |

*P < .05, **P < .01, ***P < .001.
