# Supplementary material for: Determinants of cognitive performance and decline in 20 diverse ethno-regional groups: A COSMIC collaboration cohort study
Source: PLoS Med. 2019 Jul 23;16(7):e1002853. doi: 10.1371/journal.pmed.1002853 (PMC6650056; doi:10.1371/journal.pmed.1002853)
Supplement: S28 Table — (DOCX) [file pmed.1002853.s029.docx]

|  | **Model 1** | | | | **Model 2** | | | |
| --- | --- | --- | --- | --- | --- | --- | --- | --- |
|  | **Global cognition** | | **MMSE** | | **Global cognition** | | **MMSE** | |
|  | **B (SE)** | **I^2^ (%)** | **B (SE)** | **I^2^ (%)** | **B (SE)** | **I^2^ (%)** | **B (SE)** | **I^2^ (%)** |
| Alcohol, 1 drink/week | 0.064 (0.13) | 69.1 | -0.023 (0.03) | 0 | -0.001 (0.115) | 60.9 | -0.03 (0.032) | 0 |
| Alcohol, 2+ drinks/week | -0.002 (0.045) | 39.6 | -0.035 (0.019) | 0 | -0.029 (0.054) | 49.4 | -0.039 (0.02) | 0 |
| Alcohol, any | 0.002 (0.06) | 55.3 | -0.026 (0.015) | 0 | -0.032 (0.057) | 51.0 | -0.039 (0.019)* | 0 |
| *APOE*4* | -0.028 (0.043) | 25.6 | -0.031 (0.017) | 3.6 | -0.053 (0.055) | 41.6 | -0.035 (0.03) | 30.0 |
| Body mass index |  |  |  |  | 0.003 (0.003) | 0 | 0.002 (0.003) | 39.0 |
| Cholesterol, high | 0.05 (0.024)* | 0 | 0.024 (0.016) | 6.7 | 0.036 (0.025) | 0 | 0.011 (0.025) | 22.8 |
| Cardiovascular disease | -0.004 (0.028) | 0 | -0.003 (0.034) | 49.1 | 0.02 (0.03) | 0 | 0.019 (0.044) | 54.6 |
| Depression |  |  |  |  | -0.044 (0.068) | 38.8 | -0.003 (0.035) | 32.5 |
| Diabetes | 0.023 (0.037) | 0 | 0.023 (0.037) | 44.1 | 0.03 (0.04) | 0 | 0.05 (0.042) | 36.0 |
| Hypertension | -0.039 (0.069) | 62.6 | -0.012 (0.014) | 0 | -0.025 (0.071) | 56.3 | -0.014 (0.018) | 0 |
| Physical Activity, any |  |  |  |  | -0.028 (0.032) | 0.5 | -0.014 (0.025) | 8.3 |
| Physical Activity, moderate |  |  |  |  | -0.034 (0.034) | 0 | -0.006 (0.032) | 22.4 |
| Physical activity, vigorous |  |  |  |  | -0.031 (0.063) | 36.3 | -0.016 (0.035) | 22.4 |
| Smoke, ever | -0.006 (0.032) | 16.3 | 0.042 (0.026) | 31.9 | 0.02 (0.03) | 9.9 | 0.047 (0.026) | 33.3 |
| Smoking, current | 0.14 (0.056)* | 0 | 0.098 (0.064) | 50.0 | 0.14 (0.056)* | 0 | 0.095 (0.062) | 47.5 |
| Smoking, past | 0.012 (0.025) | 0 | 0.043 (0.027) | 31.0 | 0.007 (0.038) | 22.8 | 0.042 (0.027) | 31.8 |
| Stroke | -0.052 (0.06) | 0 | -0.071 (0.062) | 54.4 | -0.058 (0.07) | 5.7 | -0.06 (0.077) | 52.3 |

*P < .05, **P < .01, ***P < .001.
