# Supplementary material for: Determinants of cognitive performance and decline in 20 diverse ethno-regional groups: A COSMIC collaboration cohort study
Source: PLoS Med. 2019 Jul 23;16(7):e1002853. doi: 10.1371/journal.pmed.1002853 (PMC6650056; doi:10.1371/journal.pmed.1002853)
Supplement: S29 Table — (DOCX) [file pmed.1002853.s030.docx]

|  | **With BMI** | | | | **With current depresion** | | | |
| --- | --- | --- | --- | --- | --- | --- | --- | --- |
|  | **Global cognition** | | **MMSE** | | **Global cognition** | | **MMSE** | |
|  | **B (SE)** | **I^2^ (%)** | **B (SE)** | **I^2^ (%)** | **B (SE)** | **I^2^ (%)** | **B (SE)** | **I^2^ (%)** |
| Age | -0.031 (0.005)*** | 7.4 | -0.049 (0.006)*** | 22.0 | -0.032 (0.006)*** | 39.5 | -0.052 (0.005)*** | 17.9 |
| Alcohol 1 drink/week | -0.028 (0.107) | 14.6 | 0.12 (0.103) | 5.9 | -0.022 (0.109) | 18.0 | 0.118 (0.087) | 0 |
| Alcohol 1+ drinks/week | -0.013 (0.05) | 0 | 0.077 (0.055) | 0.7 | -0.014 (0.048) | 0 | 0.067 (0.056) | 3.4 |
| Alcohol 2+ drinks/week | -0.004 (0.053) | 0 | 0.045 (0.083) | 26.1 | -0.003 (0.052) | 0 | 0.039 (0.08) | 26.8 |
| Body mass index | 0.003 (0.007) | 28.9 | -0.001 (0.01) | 43.5 |  |  |  |  |
| Cholesterol, high | 0.012 (0.047) | 0 | -0.059 (0.055) | 4.6 | 0.022 (0.046) | 0 | -0.076 (0.052) | 2.4 |
| Cardiovascular disease | -0.001 (0.058) | 0 | 0.157 (0.098) | 35.5 | -0.023 (0.055) | 0 | 0.195 (0.061)** | 0 |
| Depression |  |  |  |  | 0.001 (0.055) | 0 | -0.087 (0.089) | 31.5 |
| Diabetes | -0.128 (0.12) | 43.6 | -0.079 (0.12) | 38.6 | -0.134 (0.097) | 32.5 | -0.181 (0.126) | 49.5 |
| Education | 0.003 (0.009) | 50.8 | -0.003 (0.007) | 24.1 | -0.001 (0.009) | 54.0 | 0 (0.008) | 37.6 |
| Hypertension | 0.107 (0.091) | 46.2 | 0.05 (0.052) | 0 | 0.105 (0.073) | 34.1 | 0.053 (0.059) | 11.6 |
| Sex (male) | 0.16 (0.086) | 62.5 | 0.018 (0.076) | 51.3 | 0.119 (0.083) | 63.3 | 0.003 (0.07) | 46.1 |
| Smoke, ever | -0.065 (0.066) | 25.6 | -0.054 (0.051) | 0 | -0.026 (0.085) | 46.6 | -0.024 (0.055) | 6.4 |
| Smoking, current | -0.051 (0.198) | 58.2 | -0.131 (0.181) | 46.0 | -0.057 (0.196) | 57.4 | -0.133 (0.179) | 45.3 |
| Smoking, past | -0.054 (0.067) | 25.4 | -0.064 (0.055) | 0 | -0.051 (0.072) | 31.1 | -0.058 (0.054) | 0 |
| Stroke | -0.009 (0.149) | 23.3 | -0.159 (0.232) | 58.2 | -0.004 (0.101) | 0 | -0.092 (0.199) | 53.4 |

*P < .05, **P < .01, ***P < .001.
