# Supplementary material for: Determinants of cognitive performance and decline in 20 diverse ethno-regional groups: A COSMIC collaboration cohort study
Source: PLoS Med. 2019 Jul 23;16(7):e1002853. doi: 10.1371/journal.pmed.1002853 (PMC6650056; doi:10.1371/journal.pmed.1002853)
Supplement: S30 Table — (DOCX) [file pmed.1002853.s031.docx]

|  | **With BMI** | | | | **With current depresion** | | | |
| --- | --- | --- | --- | --- | --- | --- | --- | --- |
|  | **Global cognition** | | **MMSE** | | **Global cognition** | | **MMSE** | |
|  | **B (SE)** | **I^2^ (%)** | **B (SE)** | **I^2^ (%)** | **B (SE)** | **I^2^ (%)** | **B (SE)** | **I^2^ (%)** |
| Alcohol, 1 drink/week | 0.002 (0.038) | 14.1 | -0.032 (0.031) | 0 | 0.013 (0.041) | 18.3 | -0.025 (0.03) | 0 |
| Alcohol, 2+ drinks/week | -0.028 (0.018) | 0 | -0.037 (0.019) | 0 | -0.024 (0.018) | 0 | -0.028 (0.019) | 0 |
| Alcohol, any | -0.012 (0.015) | 0 | -0.023 (0.015) | 0 | -0.007 (0.015) | 0 | -0.02 (0.015) | 0 |
| Body mass index | 0.001 (0.002) | 1.4 | 0.003 (0.002) | 23.5 |  |  |  |  |
| Cholesterol, high | 0.014 (0.015) | 0 | 0.018 (0.015) | 5.3 | 0.019 (0.014) | 0 | 0.019 (0.017) | 12.8 |
| Cardiovascular disease | 0.023 (0.019) | 0 | 0.002 (0.039) | 59.2 | 0.019 (0.018) | 0 | -0.001 (0.036) | 55.5 |
| Depression, current |  |  |  |  | 0.011 (0.022) | 21.2 | 0.003 (0.026) | 33.6 |
| Diabetes | 0.065 (0.036) | 39.5 | 0.027 (0.037) | 43.7 | 0.057 (0.03) | 25.4 | 0.012 (0.04) | 52.9 |
| Hypertension | -0.017 (0.023) | 35.4 | -0.011 (0.014) | 0 | -0.023 (0.024) | 39.0 | -0.012 (0.016) | 9.5 |
| Smoke, ever | 0.013 (0.035) | 58.2 | 0.04 (0.023) | 21.4 | 0.014 (0.036) | 59.5 | 0.034 (0.028) | 39.0 |
| Smoking, current | 0.079 (0.069) | 59.0 | 0.095 (0.057) | 42.4 | 0.082 (0.07) | 59.5 | 0.094 (0.058) | 43.8 |
| Smoking, past | 0.002 (0.039) | 63.8 | 0.041 (0.018)* | 0 | 0.003 (0.039) | 64.4 | 0.039 (0.02) | 10.9 |
| Stroke | -0.069 (0.049) | 36.0 | -0.07 (0.064) | 57.5 | -0.064 (0.037) | 13.8 | -0.048 (0.054) | 47.6 |

*P < .05, **P < .01, ***P < .001.
