# Supplementary material for: Determinants of cognitive performance and decline in 20 diverse ethno-regional groups: A COSMIC collaboration cohort study
Source: PLoS Med. 2019 Jul 23;16(7):e1002853. doi: 10.1371/journal.pmed.1002853 (PMC6650056; doi:10.1371/journal.pmed.1002853)
Supplement: S31 Table — (DOCX) [file pmed.1002853.s032.docx]

|  | **Moderator** | | | | **Asian** | | | | **White** | | | |
| --- | --- | --- | --- | --- | --- | --- | --- | --- | --- | --- | --- | --- |
|  | **Global cognition** | | **MMSE** | | **Global cognition** | | **MMSE** | | **Global cognition** | | **MMSE** | |
|  | **B (SE)** | **I^2^ (%)** | **B (SE)** | **I^2^ (%)** | **B (SE)** | **I^2^ (%)** | **B (SE)** | **I^2^ (%)** | **B (SE)** | **I^2^ (%)** | **B (SE)** | **I^2^ (%)** |
| Age-squared | 0.001 (<0.001)* | 0 | <0.001 (<0.001) | 39.122 | <0.001 (<0.001) | 22.3 | -0.001 (0.001) | 81.857 | -0.001 (<0.001)** | 0 | -0.001 (<0.001)*** | 10.002 |
| Alcohol, 1 drink/week | 0.262 (0.26) | 0 | 0.225 (0.272) | 16.5 | 0.281 (0.544) | 62.1 | 0.391 (0.24) | 0 | 0.086 (0.092) | 0 | 0.174 (0.105) | 26.9 |
| Alcohol, 2+ drinks/week | -0.063 (0.145) | 0 | -0.022 (0.146) | 13.7 | 0.028 (0.136) | 0 | 0.138 (0.194) | 25.3 | 0.091 (0.048) | 0 | 0.098 (0.04)* | 15.5 |
| Alcohol, any | -0.223 (0.164) | 0 | -0.179 (0.18) | 15.9 | 0.188 (1.99) | 4.9 | 0.001 (0.301) | 33.6 | 0.094 (0.051) | 0 | 0.085 (0.055) | 18.4 |
| Anxiety | -0.305 (0.224) | 0 | -0.443 (0.253) | 24.6 | -0.646 (0.475) | 65.2 | -0.682 (0.453) | 39.9 | -0.121 (0.079) | 0 | -0.042 (0.084) | 23.5 |
| *APOE*4* | -0.223 (0.171) | 7.6 | 0.343 (0.146)* | 3.8 | -0.375 (0.156)* | 0 | 0.277 (0.138)* | 0 | -0.155 (0.064)* | 13.3 | -0.066 (0.046) | 5.7 |
| Atrial fibrillation | -2.99 (2.583) | 71.5 | -0.34 (0.457) | 0 | -4.057 (1.688)* | 0 | -0.029 (0.393) | 0 | -1.067 (1.227) | 71.5 | 0.311 (0.232) | 0 |
| Body mass index | -0.005 (0.004) | 0 | 0 (0.003) | 0 | -0.007 (0.004) | 0 | 0 (0.003) | 0 | -0.002 (0.001)* | 0 | <0.001 (<0.001) | 0 |
| Body mass index-squared | -0.024 (0.019) | 0 | 0 (0.015) | 0 | -0.024 (0.019) | 0 | -0.002 (0.014) | 0 | 0 (0.006) | 0 | -0.002 (0.003) | 0 |
| Cholesterol, high | -0.009 (0.115) | 0 | -0.023 (0.099) | 0 | -0.035 (0.105) | 0 | -0.025 (0.114) | 26.7 | -0.026 (0.048) | 0 | -0.01 (0.044) | 0 |
| Cardiovascular disease | -0.324 (0.182) | 49.8 | -0.233 (0.126) | 17.5 | -0.377 (0.338) | 81.6 | -0.121 (0.229) | 65.5 | 0.012 (0.056) | 15.0 | <0.001 (<0.001) | 0 |
| Diastolic blood pressure | 0.006 (0.008) | 14.6 | 0.002 (0.006) | 0 | 0.01 (0.007) | 0 | 0.002 (0.005) | 0 | 0.004 (0.003) | 18.1 | 0 (0.002) | 0 |
| Depression, current | -0.041 (0.246) | 70.3 | 0.081 (0.112) | 0 | -0.311 (0.153)* | 51.5 | -0.03 (0.143) | 26.7 | -0.275 (0.145) | 73.3 | -0.138 (0.034)*** | 0 |
| Depression, history | -0.024 (0.214) | 0 | 0.487 (0.232)* | 3.4 | -0.059 (0.204) | 0 | -12.413 (17.18)^a^ | 71.9 | -0.035 (0.065) | 0 | -0.021 (0.046) | 4.0 |
| Diabetes | -0.105 (0.133) | 0 | -0.011 (0.117) | 7.6 | -0.321 (0.116)** | 0 | -0.177 (0.106) | 0 | -0.216 (0.065)*** | 0 | -0.165 (0.044)*** | 13.0 |
| Education | 0.011 (0.053) | 82.6 | -0.035 (0.037) | 82.3 | 0.272 (0.031)*** | 74.4 | 0.185 (0.021)*** | 46.9 | 0.256 (0.034)*** | 84.6 | 0.219 (0.021)*** | 86.5 |
| Education-squared | 0.003 (0.003) | 59.9 | -0.001 (0.002) | 74.7 | 0 (0.001) | 51.8 | -0.006 (0.002)*** | 61.9 | -0.004 (0.002) | 68.2 | -0.005 (0.001)*** | 76.3 |
| Health, good | 0.167 (0.234) | 63.3 | 0.1 (0.103) | 0 | -0.124 (0.102) | 0 | 0.073 (0.136) | 39.5 | -0.213 (0.148) | 65.5 | -0.039 (0.038) | 0 |
| Health, poor | 0.023 (0.213) | 38.0 | 0.266 (0.122)* | 0 | -0.553 (0.13)*** | 0 | -0.019 (0.172) | 45.6 | -0.535 (0.135)*** | 44.2 | -0.277 (0.043)*** | 0 |
| Hypertension | 0.107 (0.101) | 0 | 0.077 (0.082) | 0 | -0.008 (0.089) | 0 | 0.048 (0.078) | 0 | -0.115 (0.047)* | 0 | -0.029 (0.024) | 0 |
| Physical activity, any | -0.07 (0.154) | 12.9 | 0.082 (0.129) | 12.8 | -0.095 (0.298) | 64.4 | 0.145 (0.101) | 0 | 0.099 (0.06) | 0 | 0.066 (0.072) | 18.7 |
| Physical activity, mod. | 0.035 (0.216) | 33.3 | 0.217 (0.199) | 20.1 | 0.107 (0.148) | 0 | 0.24 (0.161) | 0 | 0.072 (0.094) | 33.3 | 0.023 (0.078) | 20.1 |
| Physical activity, vig. | 0.022 (0.245) | 31.2 | 0.107 (0.197) | 0 | 0.191 (0.164) | 0 | 0.267 (0.185) | 0 | 0.169 (0.11) | 31.2 | 0.16 (0.067)* | 0 |
| Pulse pressure | -0.003 (0.006) | 20.3 | 0 (0.004) | 0 | -0.003 (0.006) | 0 | -0.002 (0.004) | 0 | 0 (0.003) | 25.4 | -0.002 (0.001) | 0 |
| Peripheral vascular dis. | -0.335 (0.685) | 8.2 | 0.936 (0.796) | 0 | -0.631 (0.668) | 0 | 0.928 (0.793) | 0 | -0.296 (0.126)* | 8.2 | -0.008 (0.076) | 0 |
| Systolic blood pressure | 0 (0.005) | 38.9 | 0.002 (0.003) | 0 | 0.002 (0.004) | 0 | 0 (0.003) | 0 | 0.002 (0.003) | 49.2 | -0.001 (0.001) | 0 |
| Sex (male) | 0.334 (0.085)*** | 54.8 | 0.236 (0.121) | 92.5 | 0.209 (0.095)* | 69.7 | 0.173 (0.117) | 87.0 | -0.147 (0.042)*** | 46.9 | -0.065 (0.062) | 93.0 |
| Smoke, ever | -0.316 (0.181) | 55.7 | -0.24 (0.118)* | 8.8 | -0.249 (0.259) | 65.6 | -0.211 (0.163) | 37.0 | 0.056 (0.072) | 52.3 | -0.004 (0.027) | 9.5 |
| Smoking, current | -0.245 (0.246) | 39.5 | -0.163 (0.175) | 30.4 | -0.379 (0.254) | 46.9 | -0.204 (0.227) | 42.6 | -0.134 (0.117) | 33.7 | -0.082 (0.059) | 33.0 |
| Smoking, past | -0.248 (0.233) | 48.9 | -0.328 (0.171) | 0 | -0.147 (0.185) | 0 | -0.291 (0.215) | 22.1 | 0.104 (0.078) | 53.9 | 0.013 (0.026) | 0.0 |
| Stroke | -0.104 (0.299) | 45.0 | -0.014 (0.222) | 42.9 | -0.437 (0.259) | 31.8 | -0.287 (0.175) | 0 | -0.333 (0.15)* | 48.4 | -0.285 (0.089)** | 50.6 |

*P < .05, **P < .01, ***P < .001.

^a^ The extremely large pooled B and SE for Asian people led to these results being discounted.
