# Supplementary material for: Determinants of cognitive performance and decline in 20 diverse ethno-regional groups: A COSMIC collaboration cohort study
Source: PLoS Med. 2019 Jul 23;16(7):e1002853. doi: 10.1371/journal.pmed.1002853 (PMC6650056; doi:10.1371/journal.pmed.1002853)
Supplement: S32 Table — (DOCX) [file pmed.1002853.s033.docx]

|  | **Moderator** | | | | **Asian** | | | | **White** | | | |
| --- | --- | --- | --- | --- | --- | --- | --- | --- | --- | --- | --- | --- |
|  | **Global cognition** | | **MMSE** | | **Global cognition** | | **MMSE** | | **Global cognition** | | **MMSE** | |
|  | **B (SE)** | **I^2^ (%)** | **B (SE)** | **I^2^ (%)** | **B (SE)** | **I^2^ (%)** | **B (SE)** | **I^2^ (%)** | **B (SE)** | **I^2^ (%)** | **B (SE)** | **I^2^ (%)** |
| Alcohol, 1 drink/week | 0.343 (0.413) | 0 | 0.618 (0.488) | 0 | 0.764 (1.029) | 8.3 | 0.686 (0.48) | 0 | 0.118 (0.106) | 0 | 0.068 (0.085) | 0 |
| Alcohol, 2+ drinks/week | 0.271 (0.4) | 8.5 | 0.331 (0.463) | 0 | 0.393 (0.391) | 0 | 0.451 (0.461) | 0 | 0.122 (0.066) | 11.6 | 0.119 (0.049)* | 0 |
| Alcohol, any | -1.166 (1.782) | 15.6 | -2.287 (1.557) | 0 | -1.046 (1.779) | 0 | -2.136 (1.556) | 0 | 0.12 (0.072) | 18.3 | 0.151 (0.054)** | 0.1 |
| Anxiety | 0.572 (0.315) | 2.3 | 0.487 (0.344) | 0 | -3.214 (5.533) | 68.6 | -4.746 (6.409) | 54.1 | 0.198 (0.119) | 2.9 | 0.093 (0.072) | 0 |
| *APOE*4* | 0.282 (0.443) | 34.1 | 0.754 (0.438) | 0 | -1.595 (1.792) | 55.7 | 0.614 (0.435) | 0 | -0.315 (0.098)** | 40.0 | -0.14 (0.051)** | 0 |
| Atrial fibrillation | -46.279 (37.323)^a^ | 72.2 | 0.266 (2.125) | 0 | -57.907 (27.819)^a^ | 0 | 0.064 (2.091) | 0 | -11.628 (15.536) | 72.2 | -0.202 (0.376) | 0 |
| Body mass index | -0.063 (0.046) | 25.9 | 0.002 (0.019) | 0 | -0.063 (0.046) | 0 | 0.001 (0.019) | 0 | -0.006 (0.008) | 97.4 | 0 (0.001) | 0 |
| Body mass index-squared | -0.278 (0.21) | 16.8 | 0.126 (0.095) | 18.3 | -0.278 (0.209) | 0 | 0.112 (0.113) | 6.0 | 0 (0.01) | 19.0 | -0.002 (0.007) | 21.8 |
| Cholesterol, high | 0.139 (0.267) | 0 | -0.218 (0.279) | 13.8 | 0.202 (0.259) | 0 | -0.222 (0.261) | 0 | 0.063 (0.065) | 0 | -0.014 (0.078) | 21.8 |
| Cardiovascular disease | -0.16 (0.273) | 0 | 0.01 (0.313) | 0.7 | 0.741 (1.379) | 32.8 | 0.212 (1.702) | 77.4 | 0.1 (0.063) | 0 | 0.127 (0.047)** | 0 |
| Diastolic blood pressure | 0.064 (0.078) | 0 | -0.03 (0.019) | 0 | 0.068 (0.078) | 0 | -0.027 (0.019) | 0 | 0.005 (0.009) | 80.8 | 0.004 (0.002) | 0 |
| Depression, current | 0.29 (0.229) | 0.4 | -0.341 (0.235) | 0.3 | 0.174 (0.215) | 0 | -0.357 (0.227) | 0 | -0.115 (0.075) | 0.1 | -0.016 (0.06) | 0 |
| Depression, history | 1.238 (2.497) | 0 | 6.663 (2.832)* | 1.4 | 1.292 (2.496) | 0 | -141.242 (200.53)^a^ | 70.4 | 0.054 (0.08) | 0 | -0.015 (0.057) | 4.0 |
| Diabetes | -0.28 (0.312) | 22.9 | -0.658 (0.27)* | 0 | -0.412 (0.246) | 0 | -0.79 (0.262)** | 0 | -0.129 (0.119) | 28.6 | -0.132 (0.064)* | 0 |
| Education | 0.003 (0.024) | 26.2 | -0.021 (0.029) | 49.9 | 0.001 (0.018) | 0 | -0.088 (0.067) | 73.1 | -0.005 (0.011) | 32.8 | -0.004 (0.01) | 45.9 |
| Education-squared | -0.001 (0.003) | 22.3 | 0.001 (0.004) | 48.9 | 0.002 (0.006) | 82.0 | 0.001 (0.002) | 12.2 | 0.001 (0.002) | 0 | 0.001 (0.001) | 38.2 |
| Health, good | 0.003 (0.325) | 49.1 | 0.01 (0.256) | 12.5 | -0.046 (0.208) | 0 | 0.314 (0.528) | 22.7 | -0.073 (0.142) | 59.2 | 0.067 (0.056) | 17.0 |
| Health, poor | -0.039 (0.355) | 22.5 | -0.329 (0.35) | 30.2 | -0.249 (0.32) | 0.5 | -0.315 (0.315) | 0 | -0.213 (0.136) | 30.2 | 0.021 (0.09) | 39.7 |
| Hypertension | 0.088 (0.258) | 32.2 | 0.028 (0.206) | 0.4 | -0.056 (0.191) | 0 | 0.074 (0.203) | 0.1 | -0.15 (0.099) | 38.9 | 0.046 (0.039) | 0 |
| Initial Level | 0.56 (1.364) | 80.0 | 0.703 (0.798) | 82.4 | -0.083 (0.148) | 0 | -0.098 (0.148) | 0 | 5.5 (0.65)*** | 0 | 4.64 (0.39)*** | 0 |
| Physical activity, any | -1.729 (1.473) | 6.2 | -0.235 (0.716) | 53.9 | -1.709 (1.468) | 0 | -0.136 (0.677) | 0 | 0.02 (0.097) | 7.4 | 0.113 (0.136) | 58.6 |
| Physical activity, moderate | -0.491 (1.74) | 0 | 1.027 (1.953) | 52.9 | -0.467 (1.737) | 0 | 1.135 (1.937) | 0 | 0.024 (0.092) | 0 | 0.108 (0.136) | 52.9 |
| Physical activity, vigorous | -2.936 (1.938) | 26.5 | 0.934 (2.232) | 49.1 | -2.916 (1.926) | 0 | 1.052 (2.218) | 0 | 0.02 (0.142) | 26.5 | 0.118 (0.14) | 49.1 |
| Pulse pressure | -0.009 (0.065) | 0 | 0.008 (0.015) | 0 | -0.01 (0.065) | 0 | 0.012 (0.022) | 28.5 | -0.001 (0.002) | 0 | 0 (0.002) | 28.0 |
| Peripheral vascular disease | -10.48 (7.829) | 49.0 | 7.085 (9.461) | 26.4 | -10.684 (7.818) | 0 | 6.997 (9.457) | 0 | -0.204 (0.269) | 49.0 | -0.088 (0.166) | 26.4 |
| Systolic blood pressure | 0.01 (0.046) | 0 | -0.006 (0.011) | 0 | 0.01 (0.046) | 0 | 0 (0.016) | 29.2 | 0.001 (0.002) | 0 | 0.001 (0.001) | 16.6 |
| Sex (male) | -0.008 (0.104) | 0.1 | 0.12 (0.117) | 24.9 | -0.058 (0.098) | 1.2 | 0.081 (0.138) | 27.9 | -0.05 (0.04) | 3.1 | -0.069 (0.035) | 29.9 |
| Smoke, ever | 0.128 (0.303) | 0 | -0.076 (0.304) | 5.2 | 0.013 (0.299) | 0 | -0.206 (0.389) | 13.5 | -0.116 (0.051)* | 0 | -0.008 (0.04) | 6.9 |
| Smoking, current | 0.112 (0.372) | 19.2 | 0.288 (0.406) | 43.7 | 0.023 (0.303) | 0 | 0.178 (0.342) | 0 | -0.078 (0.139) | 24.0 | -0.118 (0.101) | 50.4 |
| Smoking, past | -0.056 (1.973) | 0 | -1.919 (1.248) | 0 | -0.174 (1.972) | 0 | -1.917 (1.247) | 0 | -0.118 (0.053)* | 0 | 0.002 (0.039) | 0 |
| Stroke | 0.104 (0.429) | 8.4 | -0.361 (0.454) | 19.6 | -0.315 (0.38) | 0 | -1.338 (1.422) | 44.7 | -0.418 (0.15)** | 11.3 | -0.168 (0.11) | 23.7 |
| Time^b^ | 0.226 (0.706) | 98.24 | 0.227 (0.574) | 98.87 | -0.733 (0.557) | 50.44 | -0.359 (0.519) | 84.45 | -1.022 (0.352)** | 98.68 | -0.58 (0.279)* | 99.12 |
| Time-squared^b^ | 0.074 (0.069) | 87.55 | 0.017 (0.129) | 99.16 | -0.114 (0.168) | 62.82 | -0.085 (0.097) | 41.72 | -0.058 (0.023)* | 89.92 | -0.096 (0.057) | 99.42 |

*P < .05, **P < .01, ***P < .001.

^a^ The extremely large pooled B and SE for Asian people led to these results being discounted.

^b^ Coefficients from model that did not include risk factors.
