# Supplementary material for: Determinants of cognitive performance and decline in 20 diverse ethno-regional groups: A COSMIC collaboration cohort study
Source: PLoS Med. 2019 Jul 23;16(7):e1002853. doi: 10.1371/journal.pmed.1002853 (PMC6650056; doi:10.1371/journal.pmed.1002853)
Supplement: S33 Table — (DOCX) [file pmed.1002853.s034.docx]

|  | **Moderator** | | | | **Asian** | | | | **White** | | | |
| --- | --- | --- | --- | --- | --- | --- | --- | --- | --- | --- | --- | --- |
|  | **Global cognition** | | **MMSE** | | **Global cognition** | | **MMSE** | | **Global cognition** | | **MMSE** | |
|  | **B (SE)** | **I^2^ (%)** | **B (SE)** | **I^2^ (%)** | **B (SE)** | **I^2^ (%)** | **B (SE)** | **I^2^ (%)** | **B (SE)** | **I^2^ (%)** | **B (SE)** | **I^2^ (%)** |
| Alcohol, 1 drink/week | 0.228 (0.204) | 18.7 | -0.191 (0.212) | 4.2 | 0.222 (0.203) | 0 | -0.199 (0.211) | 0 | -0.007 (0.015) | 20.5 | -0.008 (0.012) | 5.8 |
| Alcohol, 2+ drinks/week | -0.159 (0.424) | 0 | -0.675 (0.424) | 0 | -0.167 (0.424) | 0 | -0.685 (0.424) | 0 | -0.005 (0.016) | 25.9 | -0.012 (0.016) | 28.7 |
| Alcohol, any | 0.312 (0.243) | 59.0 | -0.059 (0.241) | 4.6 | 0.321 (0.228) | 0 | -0.048 (0.24) | 0 | 0.015 (0.048) | 67.3 | 0.011 (0.022) | 7.2 |
| Anxiety | 0.352 (0.261) | 58.5 | 0.159 (0.179) | 0 | -0.698 (1.312) | 67.8 | -1.015 (1.458) | 50.4 | -0.165 (0.107) | 61.9 | -0.025 (0.025) | 0 |
| *APOE*4* | -0.069 (0.195) | 0 | 0.344 (0.2) | 0 | -0.438 (0.479) | 55.5 | 0.317 (0.2) | 0 | -0.015 (0.011) | 0 | -0.028 (0.011)* | 0 |
| Atrial fibrillation | -11.95 (9.537) | 70.9 | 0.273 (0.767) | 0 | -14.586 (7.479) | 0 | 0.251 (0.748) | 0 | -2.636 (3.706) | 70.9 | -0.022 (0.17) | 0 |
| Body mass index | -0.014 (0.011) | 13.0 | -0.001 (0.006) | 14.9 | -0.014 (0.011) | 0 | -0.002 (0.006) | 0 | -0.001 (0.003) | 98.5 | <0.001 (0.001) | 18.0 |
| Body mass index-squared | -0.066 (0.052) | 0 | 0.036 (0.031) | 8.4 | -0.062 (0.052) | 0 | 0.032 (0.037) | 11.5 | 0.005 (0.002)** | 0 | 0.001 (0.002) | 10.3 |
| Cholesterol, high | 0.132 (0.14) | 0 | -0.296 (0.132)* | 0 | 0.181 (0.139) | 0 | -0.307 (0.13)* | 0 | 0.05 (0.023)* | 0 | -0.011 (0.02) | 0 |
| Cardiovascular disease | 0.138 (0.155) | 48.7 | -0.097 (0.149) | 30.7 | 0.303 (0.328) | 27.9 | 0 (0.31) | 41.8 | 0.009 (0.027) | 56.0 | -0.022 (0.013) | 13.8 |
| Diastolic blood pressure | 0.015 (0.019) | 2.3 | 0.002 (0.012) | 0 | 0.014 (0.019) | 0 | 0.002 (0.012) | 0 | -0.001 (0.001) | 0.4 | 0 (0.001) | 0 |
| Depression, current | 0.088 (0.122) | 0.2 | -0.152 (0.129) | 2.5 | 0.089 (0.12) | 0 | 0.13 (0.46) | 54.8 | 0.001 (0.026) | 2.4 | 0.004 (0.017) | 3.3 |
| Depression, history | 0.35 (0.61) | 43.6 | 1.534 (0.688)* | 52.2 | 0.352 (0.602) | 0 | -32.67 (46.575) | 70.1 | 0.002 (0.062) | 43.6 | 0.005 (0.029) | 57.2 |
| Diabetes | -0.091 (0.138) | 2.1 | -0.296 (0.137)* | 10.2 | -0.047 (0.136) | 0 | -0.256 (0.155) | 5.4 | 0.044 (0.02)* | 2.9 | 0.025 (0.016) | 11.7 |
| Education | 0.027 (0.012)* | 47.8 | -0.024 (0.011)* | 0 | 0.016 (0.024) | 39.0 | -0.044 (0.028) | 65.5 | -0.004 (0.003) | 54.8 | -0.001 (0.001) | 0 |
| Education-squared | -0.106 (0.116) | 0 | 0.141 (0.12) | 0 | -0.073 (0.116) | 0 | 0.135 (0.135) | 4.5 | 0.032 (0.015)* | 16.8 | -0.018 (0.01) | 4.6 |
| Health, good | -0.172 (0.157) | 10.2 | -0.225 (0.152) | 33.4 | -0.128 (0.155) | 0 | -0.23 (0.151) | 0.4 | 0.043 (0.02)* | 13.9 | -0.004 (0.02) | 42.9 |
| Health, poor | -0.181 (0.107) | 17.6 | -0.049 (0.105) | 17.0 | -0.183 (0.104) | 0 | 0.063 (0.206) | 45.1 | -0.001 (0.016) | 12.1 | -0.016 (0.009) | 0 |
| Hypertension | -0.354 (0.354) | 0 | 0.078 (0.225) | 35.9 | -0.377 (0.353) | 0 | 0.062 (0.218) | 0 | -0.024 (0.03) | 0 | -0.015 (0.038) | 40.8 |
| Initial Level | -0.073 (0.414) | 0 | 0.161 (0.472) | 54.0 | -0.098 (0.413) | 0 | 0.17 (0.463) | 0 | -0.025 (0.032) | 0 | 0.009 (0.049) | 54.0 |
| Physical activity, any | -0.67 (0.459) | 0 | 0.248 (0.536) | 41.6 | -0.694 (0.458) | 0 | 0.211 (0.53) | 0 | -0.024 (0.033) | 0 | -0.037 (0.045) | 41.6 |
| Physical activity, moderate | -0.003 (0.016) | 0 | 0.014 (0.009) | 0 | -0.003 (0.016) | 0 | 0.014 (0.009) | 0 | 0 (0.001) | 0 | <0.001 (<0.001) | 6.2 |
| Physical activity, vigorous | -2.636 (1.843) | 0 | 1.719 (2.237) | 2.2 | -2.527 (1.842) | 0 | 1.726 (2.237) | 0 | 0.109 (0.049)* | 0 | 0.007 (0.038) | 2.2 |
| Pulse pressure | 0.002 (0.012) | 0 | 0.009 (0.007) | 0 | 0.002 (0.012) | 0 | 0.009 (0.007) | 0 | <0.001 (<0.001) | 0 | <0.001 (<0.001) | 42.8 |
| Peripheral vascular disease | 0.01 (0.153) | 10.9 | 0.05 (0.149) | 0 | 0.004 (0.153) | 0 | -0.035 (0.214) | 24.9 | -0.009 (0.018) | 43.3 | -0.007 (0.008) | 0 |
| Systolic blood pressure | -0.011 (0.166) | 21.8 | 0.08 (0.173) | 63.8 | 0.023 (0.181) | 2.3 | 0.11 (0.161) | 0 | 0.021 (0.028) | 21.0 | 0.007 (0.015) | 14.7 |
| Sex (male) | 0.056 (0.473) | 19.5 | -0.396 (0.361) | 0 | 0.045 (0.473) | 0 | -0.405 (0.361) | 0 | -0.013 (0.016) | 35.0 | -0.009 (0.008) | 0 |
| Smoke, ever | -0.074 (0.212) | 4.7 | -0.068 (0.204) | 0.5 | -0.099 (0.209) | 0 | -0.408 (0.566) | 60.6 | -0.026 (0.026) | 0 | -0.017 (0.021) | 0 |
| Smoking, current | 0.228 (0.204) | 18.7 | -0.191 (0.212) | 4.2 | 0.222 (0.203) | 0 | -0.199 (0.211) | 0 | -0.007 (0.015) | 20.5 | -0.008 (0.012) | 5.8 |
| Smoking, past | -0.159 (0.424) | 0 | -0.675 (0.424) | 0 | -0.167 (0.424) | 0 | -0.685 (0.424) | 0 | -0.005 (0.016) | 25.9 | -0.012 (0.016) | 28.7 |
| Stroke | 0.312 (0.243) | 59.0 | -0.059 (0.241) | 4.6 | 0.321 (0.228) | 0 | -0.048 (0.24) | 0 | 0.015 (0.048) | 67.3 | 0.011 (0.022) | 7.2 |

*P < .05, **P < .01, ***P < .001.
