# Supplementary material for: Determinants of cognitive performance and decline in 20 diverse ethno-regional groups: A COSMIC collaboration cohort study
Source: PLoS Med. 2019 Jul 23;16(7):e1002853. doi: 10.1371/journal.pmed.1002853 (PMC6650056; doi:10.1371/journal.pmed.1002853)
Supplement: S1 Text — (DOCX) [file pmed.1002853.s035.docx]

# Prospective analysis plan

# for Lipnicki et al., Determinants of cognitive performance and decline in twenty diverse ethno-regional groups: a COSMIC collaboration cohort study.

Prepared November 11, 2017.

# Data preparation

All analyses were performed with those diagnosed with dementia at baseline removed.

## Standardisation of cognitive outcome variables (DVs)

1. Scores on each of the **five cognitive outcome variables** (MMSE, Memory, Language, Processing Speed and Executive Functions were standardised using the following steps:
   - Firstly, within each study, raw MMSE and domain scores, pooled across all waves, were transformed to have a Gaussian (or Normal) distribution, calculated so that the transformed value has the same percentile value as the original value in the original distribution. (In SPSS they are described simply as “Normal scores”, but are produced under the “Ranks Cases” procedure). Transformed scores outliers were then Winsorized to values plus or minus 3 standard deviations from the mean scores
   - To obtain standardised scores, a regression model was fit for each outcome variable, within each study, using the baseline (i.e., wave 1) sample. If a particular study did not have wave 1 data for an outcome variable, the first wave where data was collected for that variable was treated as baseline (NB: this was only the case for the PATH study, where processing speed and executive functions were measured at wave 2 onwards. Wave 2 data was treated as baseline data for this study in order to build the baseline regression model)
   - The dependent variable in the regression models were scores at baseline on each of the five outcome variables, with age (at baseline), sex, and education, and all interactions between these variables as predictors
   - Each regression model and standard error of the estimate (SEM) for each study/variable combination was saved (see Figure below for saved standard errors from regression models produced for each study/outcome variable combination)


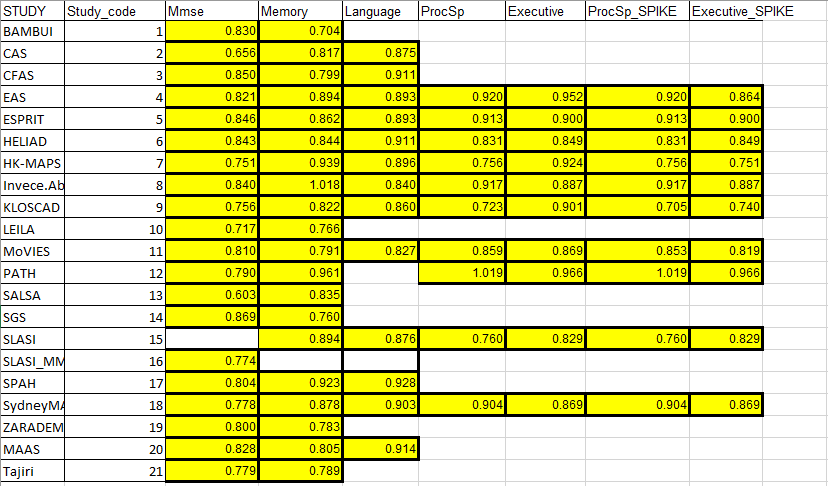


- - The regression models were then used to estimate predicted mean values on each outcome variable (within each study), substituting the mean value of age at baseline (M = 72.7 years), years of education (M = 9.0), and sex (M = 0.42, representing the proportion of males across studies at baseline).
  - Standardised scores for all outcome variables for each study, at each wave, were then calculated by subtracting the predicted values (obtained above) from the normalised scores at each wave and dividing this difference by the relevant SEM (i.e., from the model based with the same study and variable combination).
  - A global cognition score was also obtained for participants’ that had results for 3 or more of the outcome variables (excluding MMSE). Scores on the available outcome measures were averaged across participants in each wave, within each study and then normalized (within study and wave). These scores were converted to standardised scores using the same process described above. Namely, a regression model was fit using the averaged score on wave 1 as the dependent variable, and age (at baseline), sex, and education, and all interactions between these variables as predictors. The regression model and standard error of the estimate (SEM) for each study was saved. Predicted mean global cognition scores were obtained by substituting the mean value of age at baseline (M = 72.7 years), years of education (M = 9.0), and sex (M = 0.42) into the regression model.
  - Standardised global cognition scores were then calculated by subtracting the predicted mean value (obtained from the regression equation) from participants’ normalized averaged scores at each wave, and we divided this difference by the relevant SEM (i.e., from the model based with the same study and variable combination).

# LMMs for the effects of risk factors on level of cognition and decline

## Associations of age at baseline, sex and education with cognitive performance/decline

1. Following data preparation, we fit separate linear mixed models to examine the associations between age at baseline, sex, education with cognitive performance/decline on each of the outcome variables. This model did not include risk factors. In this model was also treated as data exploration to determine the selection of model terms to include in subsequent linear mixed models where risk factors were included.
2. The linear mixed models were fit for each study/outcome variable combination and included the following fixed effects terms^[[1]](#footnote-1)^:
   - Time in study (TIS; ***centred*** at approx. mean = 3.1 years)
   - TIS squared^[[2]](#footnote-2)^ (TIS^2^)
   - Age at baseline (AgeBL; ***centred*** at approx. mean 72.7 years)
   - AgeBL-squared (AgeBL^2^)
   - Education (Educ; ***centred*** at approx. mean = 9.0 years)
   - Sex (Sex, ***centred*** at the approx. mean of 0.42)
   - AgeBL*Sex
   - AgeBL*Educ
   - AgeBL*TIS
   - Educ*TIS
   - Sex*TIS
3. A random intercept and random effect for TIS was included in the analysis, with an unstructured covariance between the random intercept and slope

The above linear mixed model, is expressed mathematically in the equation below:

Y*_ti_* = [γ_00_ + γ_10_(*TIS_ti_*) + γ_20_(*TIS^2^_ti_*)
+ γ_01_(*AGEBL_i_*) + γ_02_(*AGEBL*^2^*_i_*) + γ_03_(*SEX_i_*) + γ_04_(*EDUCATION_i_*) + γ_05_(*AGEBL_i_*)(*SEX_i_*) + γ_06_(*AGEBL_i_*)(*EDUCATION_i_*)
+ γ_11_(*TIS_ti_*)(*AGEBL_i_*) + γ_12_(*TIS_ti_*)(*SEX_i_*) + γ_13_(*TIS_ti_*) (*EDUCATION_i_*)]

+ [ζ _0_*_i_*  + ζ _1_*_i_*(*TIS_ti_*) + *ε*_t_*_i_*]

(1)

Random effects

Fixed effects

Where Y*_ti_* is the score for individual *i* at time *t*
γ_00_ is the fixed effect for the intercept, or the average outcome score when all predictors are 0. Since Time in Study (TIS) was centred at the mean of 3.12 years, it is the mean level of performance on the outcome at 3.12 years, and when all other predictors are 0;
γ_10_ is the fixed effect for time in study (TIS), indicating the rate of change in the outcome per year (i.e., the slope)
γ_01,02…0n_ is a fixed effect for predictor *n*, indicating the change in the level of the outcome variable (at the mean time in study) with a 1-unit increase on that predictor
γ_11,12…1n_ is a fixed effect for predictor *n,* indicating the change in the slope with a 1-unit increase on that predictor

γ_20_ is a fixed effect of the change in the slope with every additional year

ζ _0i_ is the deviation of individual *i*’s intercept/level of performance around the average intercept, γ_00_.

ζ _1i_ is the deviation of individual *i'*s rate of change, around the average rate of change, γ_10._

ε_t_*_i_* = represents the portion of individual i's outcome that is unpredicted at occasion *t*

AGEBL*_i_* is the age at baseline for participant i

EDUCATION*_i_* is education in years for participant i

TISt*_i_* is the time in study in years for participant i at time t

1. We then re-ran this model, but this time centring time at 0 (baseline) to investigate the possible influence of baseline performance on decline – specifically, by examining the covariance between the random intercept and slope. In a linear mixed model where time is centred at zero (baseline), the covariance term quantifies the relationship between initial status (i.e., performance at baseline) and the rate of change. Having both a positive covariance term and a negative fixed effect for time implies that lower performance at baseline is associated with a faster rate of decline.

## Associations between putative risk and protective factors and cognitive performance/decline: Partially adjusted multivariable models

1. Linear Mixed Models were then fit in *R* examining the association between a range of risk factors on both the level of cognition (at the average time in study) and cognitive decline. The following risk factors were examined in separate linear mixed models:

| RISK FACTOR (RF) | Definition |
| --- | --- |
| ***Continuous**** |  |
| Body mass index (BMI) | Body mass index centred at the approx. grand mean |
| BMI^2^ | Squared BMI centred |
| Systolic Blood Pressure (SBP) | SBP centred at the approx. grand mean |
| Diastolic Blood Pressure (DBP) | DBP centred at the approx. grand mean |
| Pulse Pressure (PP) | Pulse Pressure (SBP-DBP) centred at the approx. grand mean |
| ***Binary*** |  |
| Carriage of Apolipoprotein E4 (APOE4) | 0 = no APOE4 alleles, 1 = one or two APOE4 alleles |
| Current anxiety | Current anxiety, 0 = no, 1 = yes |
| Current Depression | Current depression, 0 = no, 1 = yes |
| History of Depression | History of depression, 0 = no, 1 = yes |
| Hypertension | Hypertension, 0 = no, 1 = yes |
| Diabetes | Diabetes, 0 = no, 1 = yes |
| High Cholesterol | High cholesterol, 0 = no, 1 = yes |
| Peripheral Vascular Disease | Peripheral Vascular Disease, 0 = no, 1 = yes |
| Atrial Fibrillation | Atrial Fibrillation, 0 = no, 1 = yes |
| Cardiovascular Disease | Cardiovascular Disease, 0 = no, 1 = yes |
| Any Alcohol use | Alcohol use, 0 = nil/minimal, 1 = one or more drinks per week |
| Any Physical Activity | Physical Activity, 0 = nil/minimal, 1 = moderate or vigorous activity at least once a week |
| Ever Smoking | Smoking, 0 = no, 1 = Past or Current |
| History of Stroke | Stroke or TIA, 0 = no, 1 = yes |
| ***Multi-Categorical*** |  |
| Body Mass Index multi-category | Body Mass Index,  0 = underweight (BMI < 17.5),  1 = Normal-I (17.5-22.5),  2 = Normal-II (22.5-25),  3 = Overweight (25-30),  4 = Obese IA (30-32.5),  5 = Obese IB (32.5+) |
| Self-rated health | Current Health, 0 to 2, ranging from best to worst |
| Physical Activity | Physical Activity, 0 = nil/minimal, 1 = moderate activity at least once a week; 2 = Vigorous activity at least once a week |
| Smoking | Smoking, 0 = no, 1 = Past, 2 = Current |
| Alcohol Use | Alcohol use, 0 = nil/minimal, 1 = one drinks per week, 2 = two or more drinks per week |
| Education Category | Education  0 = Less than 6 years  1 = 6 to <9 years  2 = 9 to < 12 years  3 = 12 years  4 = 13 to <15 years  5 = 15+ years |

*All continuous risk factors and variables were grand-mean centred, where the mean of that variable across all studies and waves was subtracted from participants’ raw score on that variable.

1. For categorical risk factors, an appropriate level was chosen as the reference category. For most categorical variables, the *absence*, or the *lowest level* of the risk factor was assigned as the reference category (i.e., Risk Factor value of 0). For the categorical version of BMI (BMI_SIX), the Normal-I category was used as the reference and for the categorical version of Education (EDUCATION_CAT), 12 years was treated as the reference category.
2. The following fixed effects were included in separate linear mixed models for each risk factor and outcome variable combination:
   - Time in study (TIS; ***centred*** at approx. mean = 3.1 years)
   - TIS squared (TIS^2^)
   - Age at baseline (AgeBL; ***centred*** at approx. mean 73.1 years)
   - AgeBL squared (AgeBL^2^)
   - Education (Educ; ***centred*** at approx. mean = 9.15 years)
   - Sex (Sex, ***centred*** at the approx. mean of 0.4)
   - AgeBL*Sex
   - AgeBL*Educ
   - AgeBL*TIS
   - Educ*TIS
   - Sex*TIS
   - Risk factor (RF)
   - RF*AgeBL
   - RF*AgeBL^2^
   - RF*Sex
   - RF*Educ
   - RF*TIS
   - RF*TIS^2^

This above linear mixed model is expressed mathematically in the equation (2) below:

Y*_ti_* = [γ_00_ + γ_10_(*TIS_ti_*) + γ_20_(*TIS^2^_ti_*)
+ γ_01_(*AGEBL_i_*) + γ_02_(*AGEBL*^2^*_i_*) + γ_03_(*SEX_i_*) + γ_04_(*EDUCATION_i_*) + γ_05_(*AGEBL_i_*)(*SEX_i_*) + γ_06_(*AGEBL_i_*)(*EDUCATION_i_*)
+ γ_11_(*TIS_ti_*)(*AGEBL_i_*) + γ_12_(*TIS_ti_*)(*SEX_i_*) + γ_13_(*TIS_ti_*) (*EDUCATION_i_*)
+ γ_07_(*RF_i_*) + γ_08_(*RF_i_*)(*AGEBL_i_*) + γ_09_(*RF_i_*) (*AGEBL^2^_i_*) + γ_010_(*RF_i_*)(*SEX _i_*) + γ_011_(*RF_i_*)(*EDUCATION_i_*)
+ γ_14_(*TIS_ti_*)(*RF_i_*)
+ γ_21_(*TIS^2^_ti_*)(*RF_i_*)]

+ [ζ _0_*_i_*  + ζ _1_*_i_*(*TIS_ti_*) + *ε*_t_*_i_*]

(2)

Random effects

Fixed effects

Where the meanings of symbols are equivalent to equation (1) above, in addition:

*RF_i_* represents the risk factor score for person *i*

1. We then fit several **multivariable** linear mixed models where multiple risk factors were combined in one model, with the aim of examining independent associations with cognitive performance and decline. Not all risk factors were included in these models as not all were available for every cohort, with the choice of risk factors used involving a compromise between the number of factors included versus the number of cohorts with data for all factors. At a minimum we wanted to include a maximum combination of risk factors that would retain at least 3 Asian studies in the analysis to perform meaningful ethnoregional comparisons (i.e., comparisons with a sufficiently large and diverse group of participants with Asian ethnicity). After in-depth data exploration of the available risk factors in each study, there were 3 specific combinations of risk factors that would retain at least 3 Asian studies, and these are shown in the table below

# Risk factor combinations to use in multivariable risk factor models

| **Covariate Combination** | **A**  **APOE +**  **HT DIAB CHOL CVD STROKE SMOKING ALC** | **B**  **BMI +  HT DIAB CHOL CVD STROKE SMOKING ALC** | **C**  **DEP +  HT DIAB CHOL CVD STROKE SMOKING ALC** |
| --- | --- | --- | --- |
| **HT** | ✓ | ✓ | ✓ |
| **DIAB** | ✓ | ✓ | ✓ |
| **CVD** | ✓ | ✓ | ✓ |
| **STROKE** | ✓ | ✓ | ✓ |
| **CHOL** | ✓ | ✓ | ✓ |
| **SMOKING** | ✓ | ✓ | ✓ |
| **ALCOHOL** | ✓ | ✓ | ✓ |
| **APOE4** | ✓ |  |  |
| **BMI** |  | ✓ |  |
| **DEP** |  |  | ✓ |
|  |  |  |  |
| **N included** | 13917 | 17270 | 18011 |
| **N excluded** | 33884 | 30531 | 29790 |
| **Percent of total included** | 29.1 | 36.1 | 37.7 |
| **No. of Cohorts included (TOTAL)** | 11 | 11 | 11 |
| **WHITE COHORTS** | 6 | 6 | 6 |
| **ASIAN COHORTS** | 3 | 3 | 3 |
| **NON-WHITE/NON-ASIAN** | 2 | 2 | 2 |

This multivariable linear mixed model is expressed mathematically in the equation (3) below:

Y*_ti_* = [γ_00_ + γ_10_(*TIS_ti_*) + γ_20_(*TIS^2^_ti_*)
+ γ_01_(*AGEBL_i_*) + γ_02_(*AGEBL*^2^*_i_*) + γ_03_(*SEX_i_*) + γ_04_(*EDUCATION_i_*) + γ_05_(*AGEBL_i_*)(*SEX_i_*) + γ_06_(*AGEBL_i_*)(*EDUCATION_i_*)
+ γ_11_(*TIS_ti_*)(*AGEBL_i_*) + γ_12_(*TIS_ti_*)(*SEX_i_*) + γ_13_(*TIS_ti_*) (*EDUCATION_i_*)

+ γ_07_(*RF_1i_*) + γ_08_(*RF_1i_*)(*AGEBL_i_*) + γ_09_(*RF_1i_*) (*AGEBL^2^_i_*) + γ_010_(*RF_1i_*)(*SEX _i_*)
+ γ_011_(*RF_1i_*)(*EDUCATION_i_*)
+ γ_14_(*TIS_ti_*)(*RF_1i_*)
+ γ_21_(*TIS^2^_ti_*)(*RF_1i_*)

+ γ_07_(*RF_2i_*) + γ_08_(*RF_2i_*)(*AGEBL_i_*) + γ_09_(*RF_2i_*) (*AGEBL^2^_i_*) + γ_010_(*RF_2i_*)(*SEX _i_*)
+ γ_011_(*RF_2i_*)(*EDUCATION_i_*)
+ γ_14_(*TIS_ti_*)(*RF_2i_*)
+ γ_21_(*TIS^2^_ti_*)(*RF_2i_*)

…

+ γ_07_(*RF_mi_*) + γ_08_(*RF_mi_*)(*AGEBL_i_*) + γ_09_(*RF_mi_*) (*AGEBL^2^_i_*) + γ_010_(*RF_mi_*)(*SEX _i_*)
+ γ_011_(*RF_mi_*)(*EDUCATION_i_*)
+ γ_14_(*TIS_ti_*)(*RF_mi_*)
+ γ_21_(*TIS^2^_ti_*)(*RF_mi_*)]

+ [ζ _0_*_i_*  + ζ _1_*_i_*(*TIS_ti_*) + *ε*_t_*_i_*]

(3)

Random effects

Fixed effects

Risk factor m

Risk factor 2

Risk factor 1

Where the meanings of symbols are equivalent to equation (1), in addition:

*RFm_i_* represents the score for person *i* on risk factor *m*

1. All 3 multivariable risk factor models in the above table were fit, although A was treated as the primary multivariable risk factor model. In each multivariable linear mixed model (i.e., A, B and C), each risk factor was included, as well as interactions between each factor and age, age squared, education, sex, time, and time squared. In fitting these models, categorical risk factors with more than 2 levels such as SMOKING and ALCOHOL were converted to binary variables, where the higher levels were collapsed and compared against the lowest level. This was done to allow for the inclusion of studies in the combination analysis which only had binary level data for these factors. Nonetheless, we ran the combination mixed models using both the binary and multi-level versions of these risk factors to better understand both the overall and dose-response relationship of a factor’s effects on cognitive performance and decline.
2. We then fit a final combination model that combined all the risk factors in Models A to C in the above table, as well as physical activity. This model used data from only nine cohorts (6 White, 1 Asian, 2 Latin American or North American Hispanic; total n = 11897). The combination model was fit in a manner comparable to step 12
3. For all combination models described in steps 11-14, we investigated whether baseline performance was related to rate of change for each of the outcome measures, using the same approach as described in step 5.
4. After all linear mixed models (models in equations (1), (2), and (3)) were fit, random effects IPD meta-analyses were performed to obtain pooled estimates across studies for all model terms (including lower and upper confidence limits) and the associated standard error, Z-value, significance level, and the measure of heterogeneity for the pooled effect (I^2^). The following pooled effect terms were of the most interest:
   - **Risk factor (RF):** this term enabled an examination of the effect of each risk factor on the “level” of cognition at the average time in study (since time in study was centred at 0). A negative pooled value for this term indicates that the presence of the risk factor is associated with lower cognition at the mean time in study (of approx. 3.1 years)
   - **RF*TIS:** this term enabled an examination of the effect of each risk factor on the rate of cognitive decline over time (at the point of the mean time in study of approx. 3.1 years). A negative pooled value for this term indicates that the presence of the risk factor is associated with a faster rate of cognitive decline.
   - **RF*TIS^2^:** this term enabled an examination of how time in study, moderates the relationship between the risk factor and cognitive decline over time. A negative pooled value for this term indicates that as time in study increases, the risk factor is associated with even faster cognitive decline, or an even faster decline in the rate of improvement (depending on the direction of the RF*TIS term).

### Moderator analysis

1. After running each of the meta-analyses for the simple, individual risk factor, and combined risk factor models, we performed metaregression to compare effect sizes for each of the model terms between studies that were primarily White versus Asian (see Table 1 in main paper for studies classified as primarily Asian and White). In these analyses, a binary, study-level variable was included in each metaregression where studies were coded: 0 = White; 1 = Asian
2. The moderator analyses yielded two main coefficients of interest – the *constant* and *moderator* coefficient:
   - Constant: Represents the value of the model term (for example, the relationship between the risk factor and performance [RF], and/or cognitive decline [RF*TIS]) for the group coded as 0 (i.e., White studies).
   - Moderator: Represents the *change* or *difference* in the size of the model term for the comparison group (i.e., Asian studies). The significance of the moderator indicated that the specific model term differed significantly between White and Asian studies
   - To meaningfully interpret the significant moderator effects, all meta-analyses described above (for simple models, individual risk factors models, and combined models) were fit separately in the primarily White and Asian studies. For terms where the moderator was significant, we examined and compared the size, direction, and significance of these terms between the White and Asian pooled study results

1. Note that we initially fit a model that included the following three-way interactions in addition to the terms described in step 3 above, however we did not include these terms in the final model because the largest proportion of variance explained for either term, on any outcome variable, was less than 0.05%

   - - AgeBL * Sex * Education
     - AgeBL*Sex*Education^2^

   [↑](#footnote-ref-1)
2. Examination of the results of this model showed that TIS^2^ was a significant predictor for 14 of 20 studies for MMSE, 10 of 20 studies for Memory, 5 of 13 studies for Language, 6 of 8 studies for Processing Speed, and 5 of 8 studies for Executive Functions. Hence TIS^2^ was included in all subsequent linear mixed models where risk factors were examined. [↑](#footnote-ref-2)
