# Supplementary material for: Determinants of cognitive performance and decline in 20 diverse ethno-regional groups: A COSMIC collaboration cohort study
Source: PLoS Med. 2019 Jul 23;16(7):e1002853. doi: 10.1371/journal.pmed.1002853 (PMC6650056; doi:10.1371/journal.pmed.1002853)
Supplement: S2 Text — (DOCX) [file pmed.1002853.s036.docx]

**Results**

S24 Table shows the results of partially adjusted models for the effects of putative risk and protective factors on cognitive performance and decline.

Cognitive performance: Higher age, depression, stroke history, poor self-rated health and diabetes were significantly associated with lower global cognition composite and MMSE scores. Other factors associated with lower global cognition composite scores were *APOE*4* carriage, hypertension, peripheral vascular disease, anxiety and higher BMI (centred at 25.2 kg/m^2^). Vigorous physical activity and higher education were associated with better global cognition composite and MMSE scores. Alcohol consumption was also associated with better MMSE scores.

Cognitive decline: Higher age and stroke history were each associated with greater decline in global cognition composite and MMSE scores. *APOE*4* carriage, past smoking and poor self-rated health were also associated with greater decline in global cognition composite scores. Higher baseline MMSE scores were associated with greater decline, while consuming two or more drinks of alcohol a week and the presence of cardiovascular disease were both associated with less decline in MMSE scores.

*Significant quadratic effects:* Details of these analyses are shown in S26 Table and S27 Table. In summary, compared to *APOE*4* non-carriers, carriers showed an accelerating rate of decline in MMSE scores with time. A similar accelerating rate of decline in MMSE scores was found for individuals with good self-rated health compared to those with very good self-rated health. Conversely, compared to individuals without high cholesterol, a slower rate of decline in global cognition composite scores for those with high cholesterol became even slower with more time in study. Furthermore, with increasing time in study there was a significant slowing in the rate of cognitive decline in the presence of diabetes, peripheral vascular disease, good and poor self-rated health, and higher BMI.

**Discussion**

The factors showing associations with performance or decline in partially adjusted models have all been previously associated with poorer cognition, faster cognitive decline, or cognitive disorders in late life, including hypertension,^1^ BMI,^2^ peripheral vascular disease,^3^ anxiety,^4^ poor general health,^5^ stroke history,^6^ and past smoking.^7^ Alcohol consumption was associated with better performance and slower decline, as previously reported by many.^8^ However, a fully adjusted model quadratic effect indicated that the slower rate of decline associated with any alcohol consumption attenuated over time (S28 Table), which might involve the recently reported development of deleterious effects of alcohol on cognition with increasing age.^9^

**References**

1. Goshgarian C, Gorelick PB. Perspectives on the relation of blood pressure and cognition in the elderly. Trends Cardiovasc Med 2018.

2. Kivimaki M, Luukkonen R, Batty GD, et al. Body mass index and risk of dementia: Analysis of individual-level data from 1.3 million individuals. Alzheimers Dement 2018;14:601-9.

3. Guerchet M, Aboyans V, Nubukpo P, Lacroix P, Clement JP, Preux PM. Ankle-brachial index as a marker of cognitive impairment and dementia in general population. A systematic review. Atherosclerosis 2011;216:251-7.

4. Fung AWT, Lee JSW, Lee ATC, Lam LCW. Anxiety symptoms predicted decline in episodic memory in cognitively healthy older adults: A 3-year prospective study. Int J Geriatr Psychiatry 2018;33:748-54.

5. Sargent-Cox K, Cherbuin N, Sachdev P, Anstey KJ. Subjective health and memory predictors of mild cognitive disorders and cognitive decline in ageing: the Personality and Total Health (PATH) through Life Study. Dement Geriatr Cogn Disord 2011;31:45-52.

6. Tang EY, Amiesimaka O, Harrison SL, et al. Longitudinal Effect of Stroke on Cognition: A Systematic Review. J Am Heart Assoc 2018;7.

7. Mons U, Schottker B, Muller H, Kliegel M, Brenner H. History of lifetime smoking, smoking cessation and cognitive function in the elderly population. Eur J Epidemiol 2013;28:823-31.

8. Wardzala C, Murchison C, Loftis JM, et al. Sex differences in the association of alcohol with cognitive decline and brain pathology in a cohort of octogenarians. Psychopharmacology (Berl) 2018;235:761-70.

9. Piumatti G, Moore SC, Berridge DM, Sarkar C, Gallacher J. The relationship between alcohol use and long-term cognitive decline in middle and late life: a longitudinal analysis using UK Biobank. J Public Health (Oxf) 2018.
